# Supplementary material for: Aeromonas hydrophila CobQ is a new type of NAD+- and Zn2+-independent protein lysine deacetylase
Source: eLife. 2025 Feb 25;13:RP97511. doi: 10.7554/eLife.97511 (PMC11856932; doi:10.7554/eLife.97511)
Supplement: Figure 1—source data 1. [file elife-97511-fig1-data1.zip › Figure 1—source data 1.pdf]

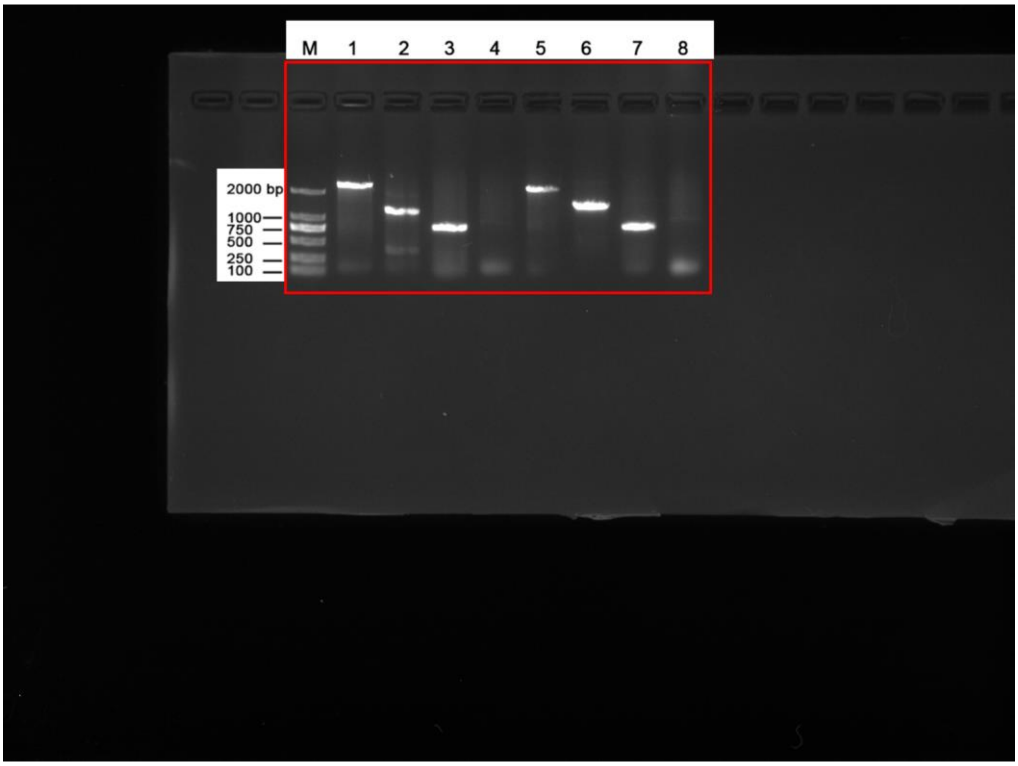

Figure 1, Source Data 1. Original PCR for Figure 3D. Construction of *ahcobB*- and *ahcobQ*-defective strains. M: Marker; Lanes 1 and 3: PCR products of WT using the P7/P8 primer pairs of *ahcobB* and *ahcobQ*, respectively; Lanes 2 and 6: PCR products of *ΔahcobB* and *ΔahcobQ*, respectively, using P7/P8 primer pairs; Lanes 5 and 7: PCR products of WT using P5/P6 primer pairs of *ahcobB* and *ahcobQ*, respectively; Lanes 4 and 8: PCR products of *ΔahcobB* and *ΔahcobQ*, respectively, using the P5/P6 primer pairs.
