# Supplementary figures and images for: Aeromonas hydrophila CobQ is a new type of NAD+- and Zn2+-independent protein lysine deacetylase
Source: eLife. 2025 Feb 25;13:RP97511. doi: 10.7554/eLife.97511 (PMC11856932; doi:10.7554/eLife.97511)

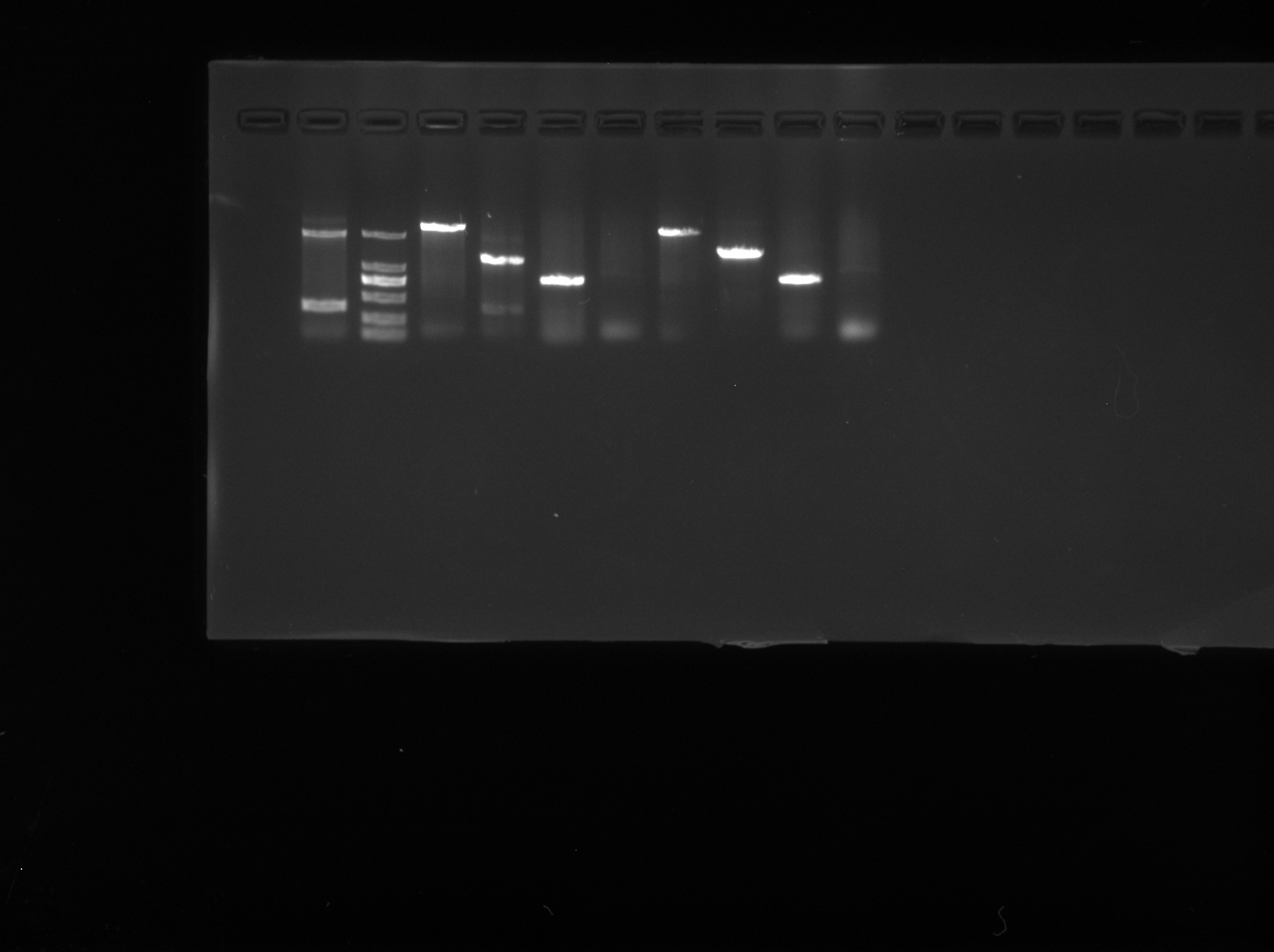

Supplement: Figure 1—source data 2. [file elife-97511-fig1-data2.zip › Figure 1-source data1.tif]

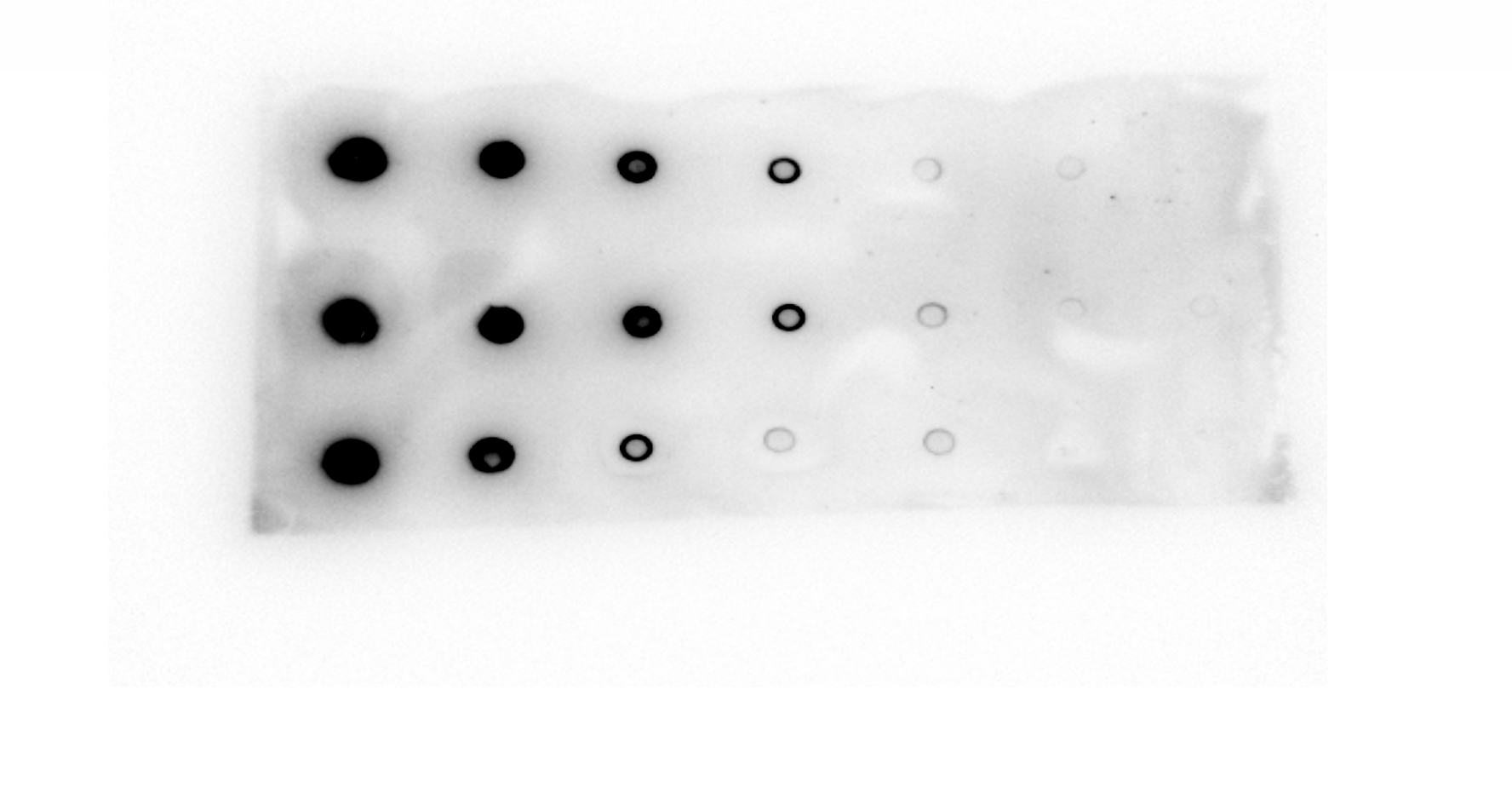

Supplement: Figure 2—source data 2. [file elife-97511-fig2-data2.zip › Figure2-source data2/dot blot of Figure2C.tif]

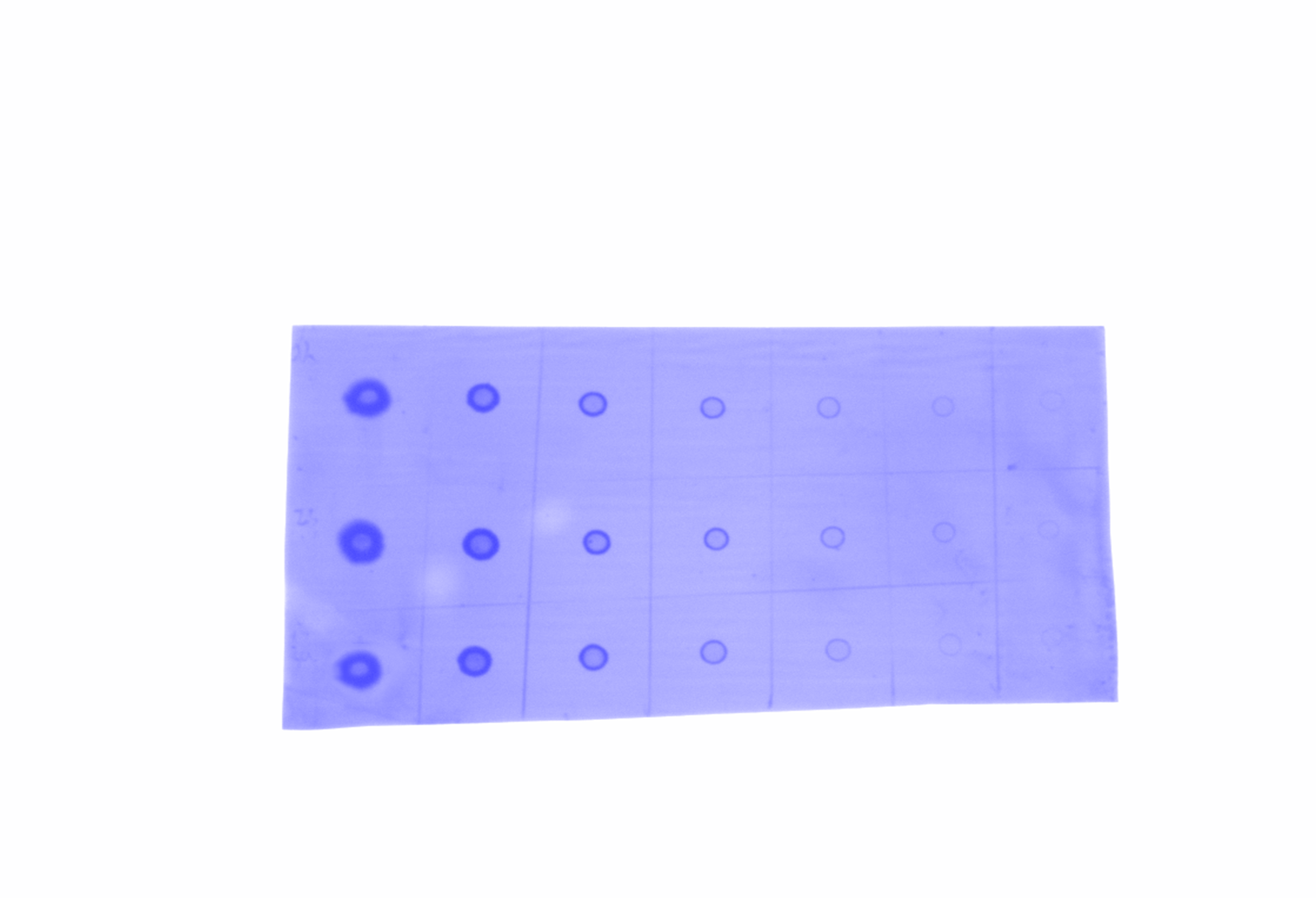

Supplement: Figure 2—source data 2. [file elife-97511-fig2-data2.zip › Figure2-source data2/loading control of Figure2C.tif]

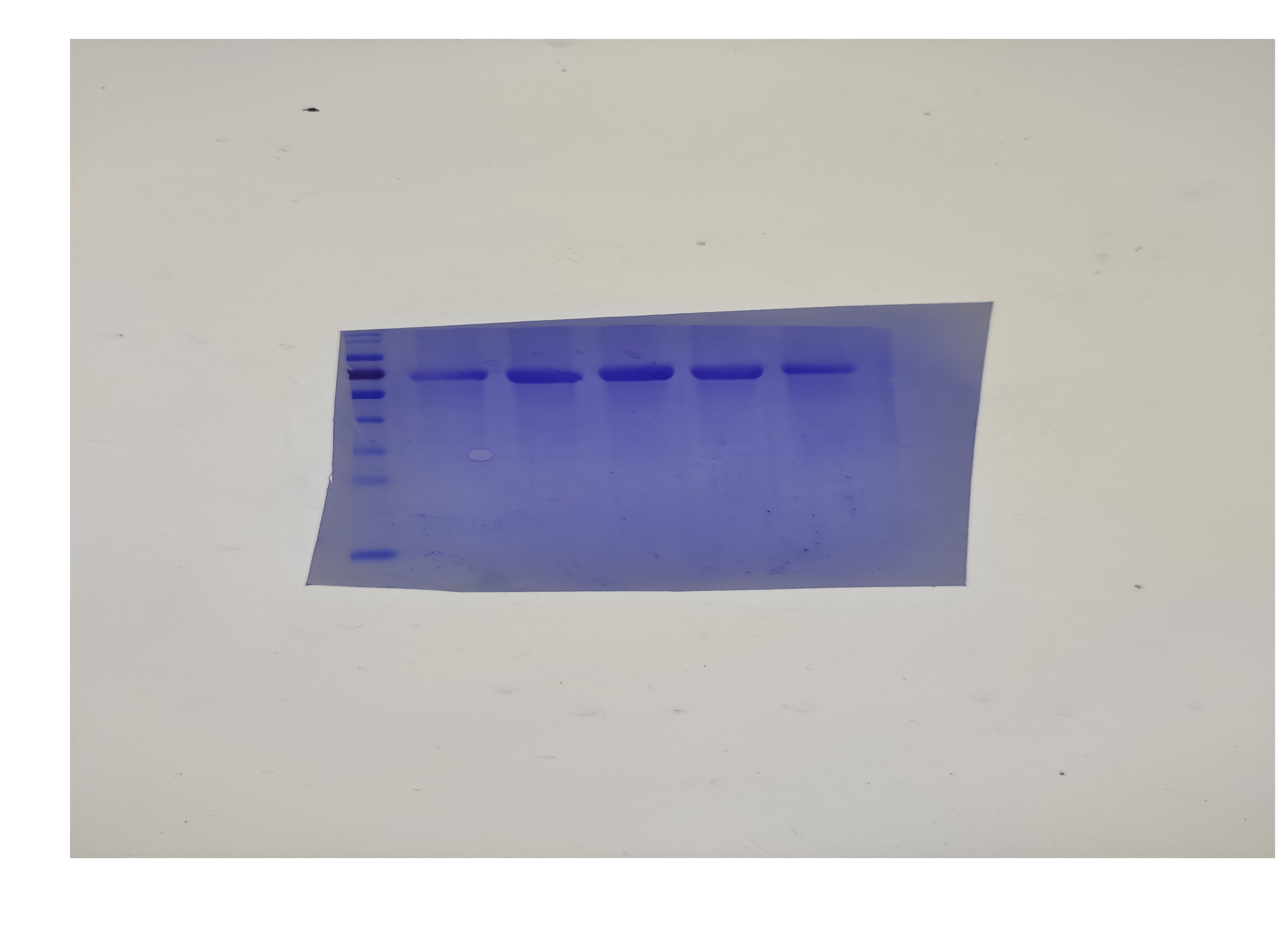

Supplement: Figure 2—source data 2. [file elife-97511-fig2-data2.zip › Figure2-source data2/loading control of Figure2E.tif]

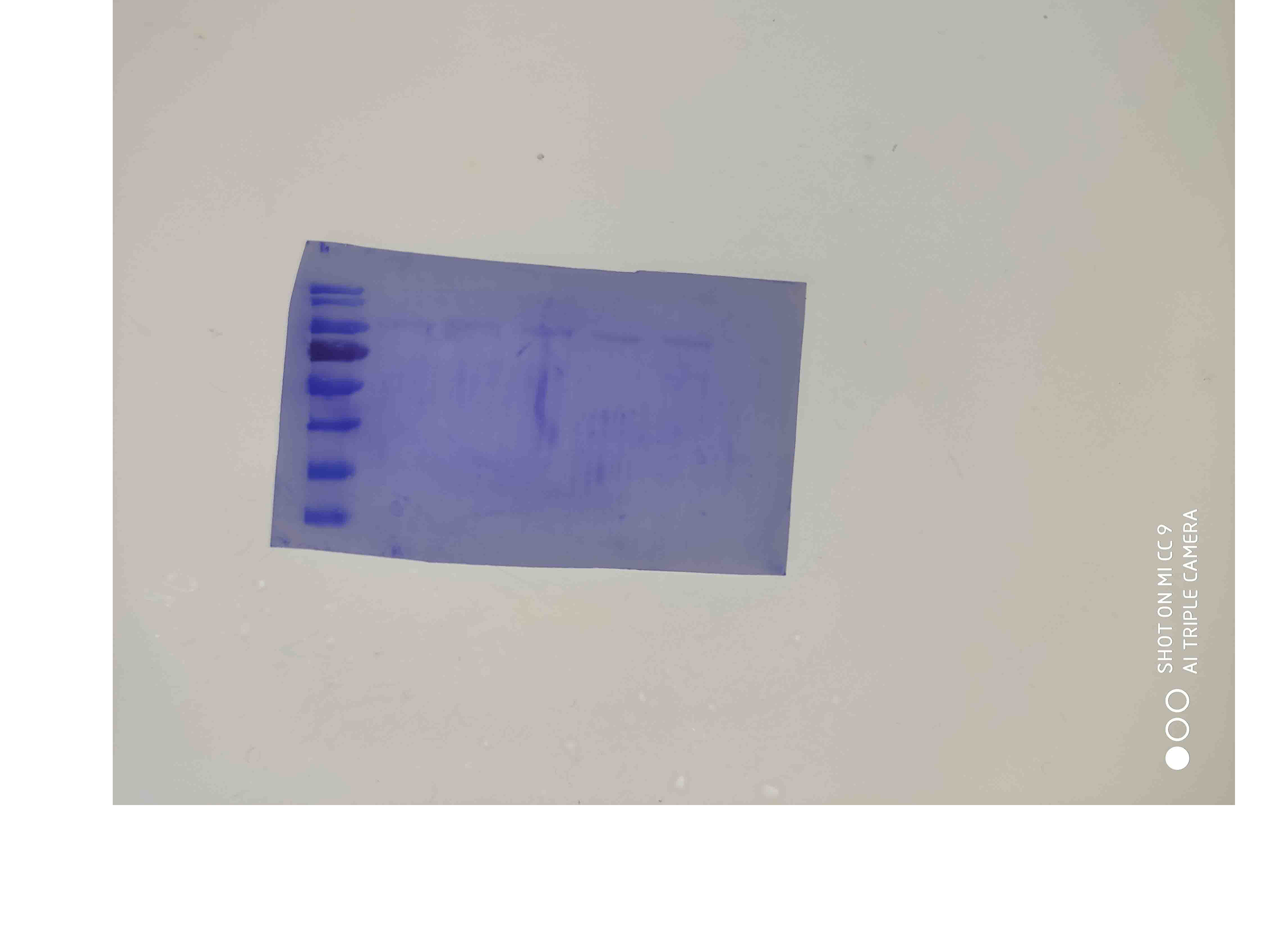

Supplement: Figure 2—source data 2. [file elife-97511-fig2-data2.zip › Figure2-source data2/loading control of Figure2F.tif]

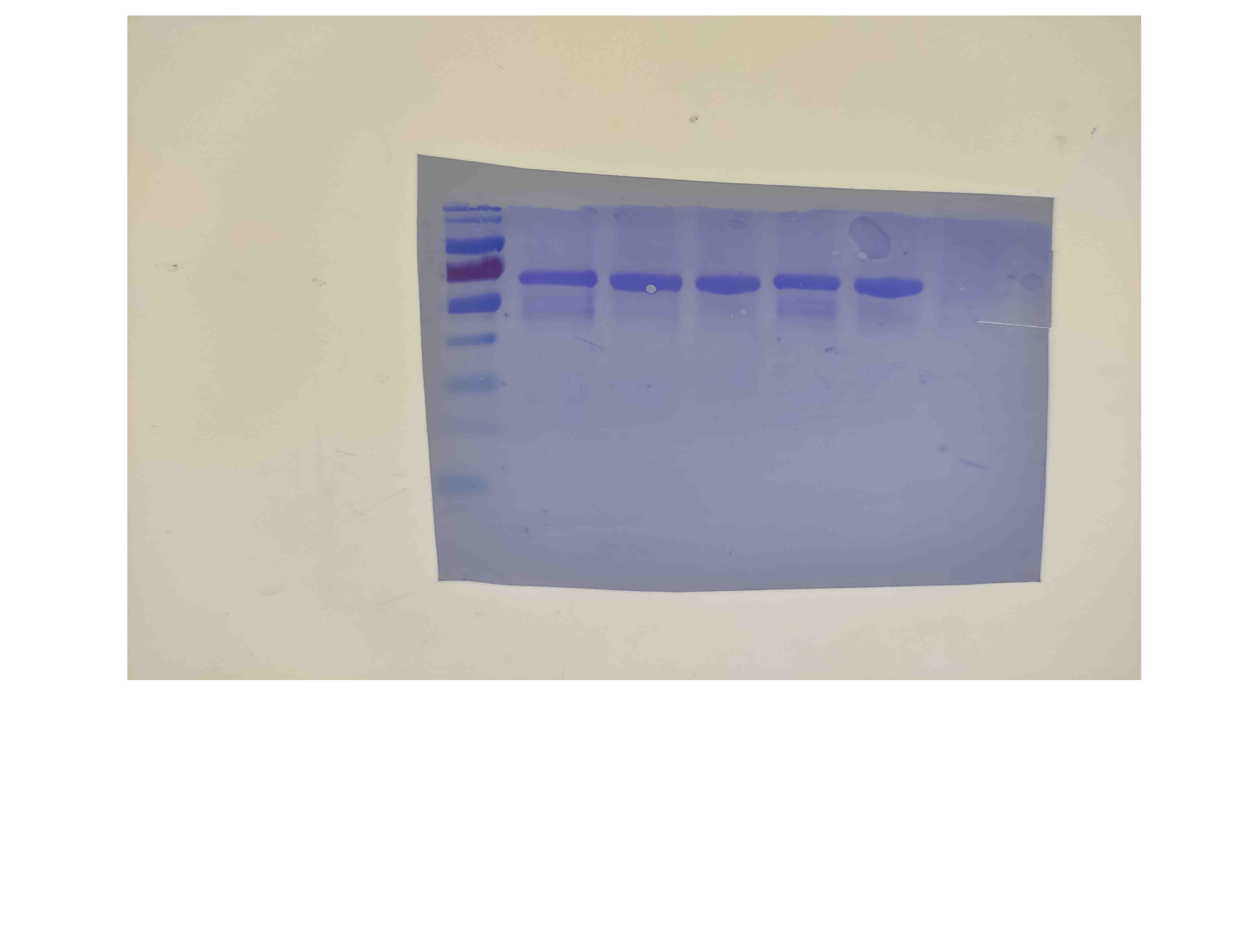

Supplement: Figure 2—source data 2. [file elife-97511-fig2-data2.zip › Figure2-source data2/loading control of Figure2G.tif]

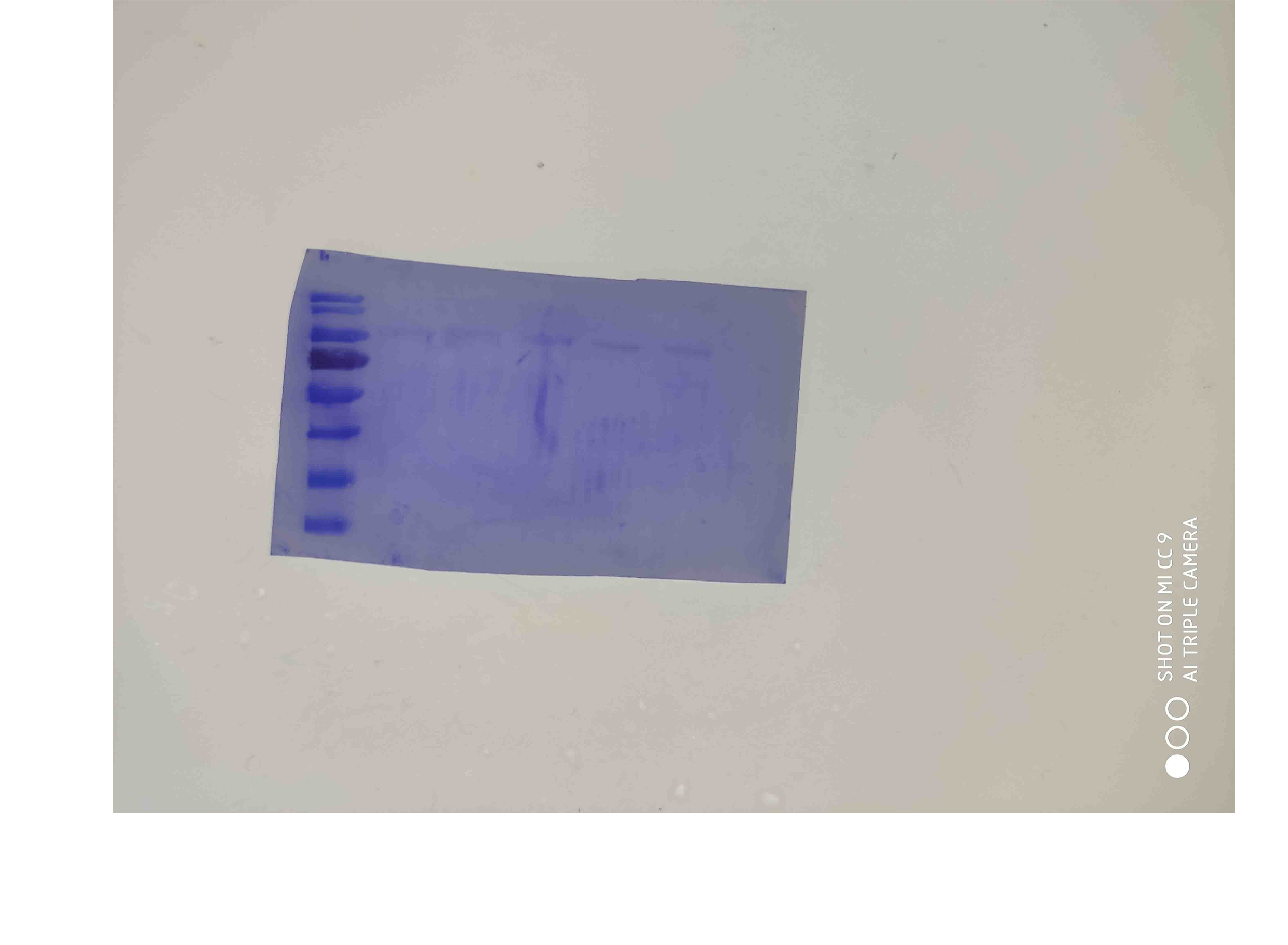

Supplement: Figure 2—source data 2. [file elife-97511-fig2-data2.zip › Figure2-source data2/loading control of Figure2H.tif]

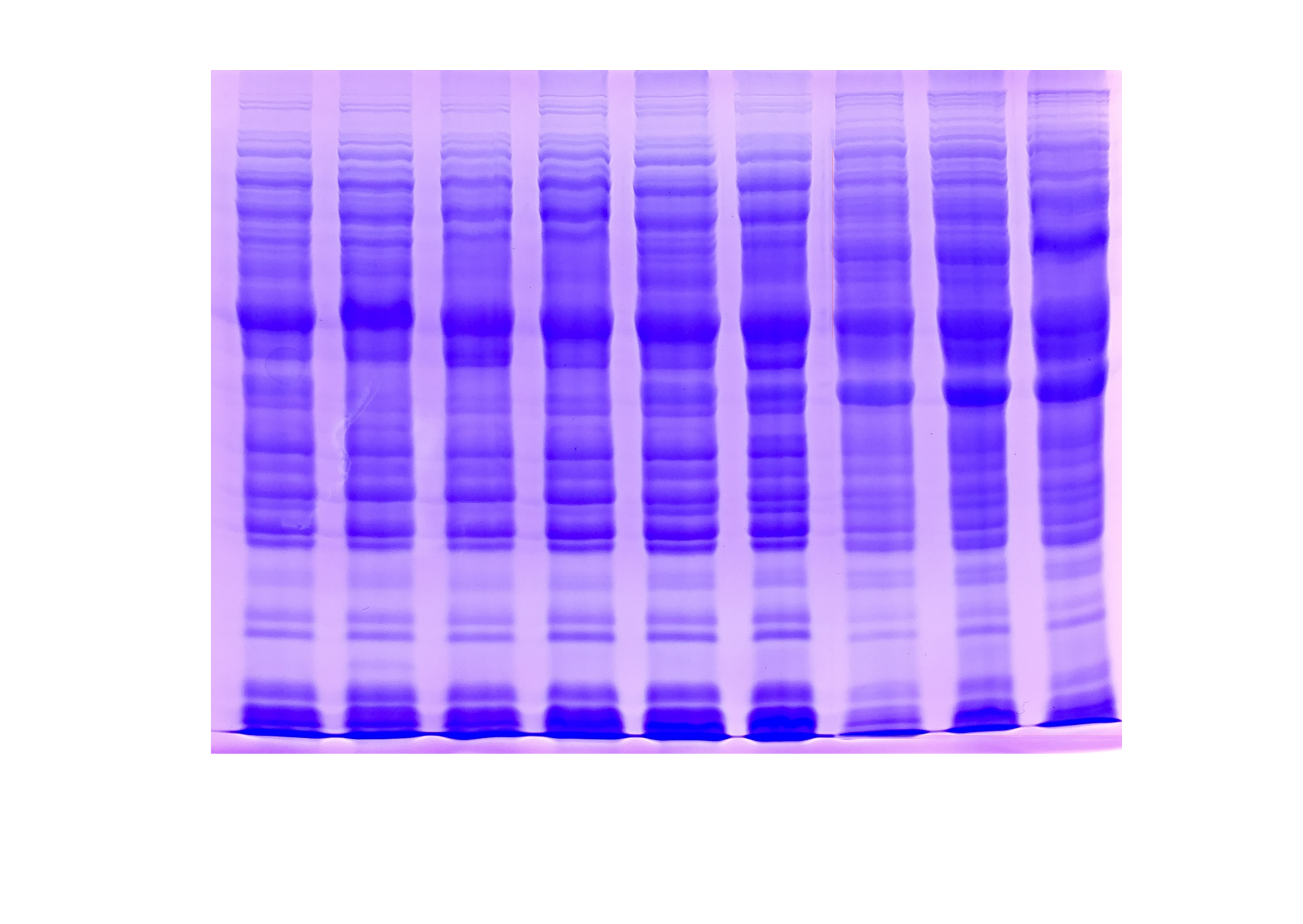

Supplement: Figure 2—source data 2. [file elife-97511-fig2-data2.zip › Figure2-source data2/SDS-PAGE of Figure2D.tif]

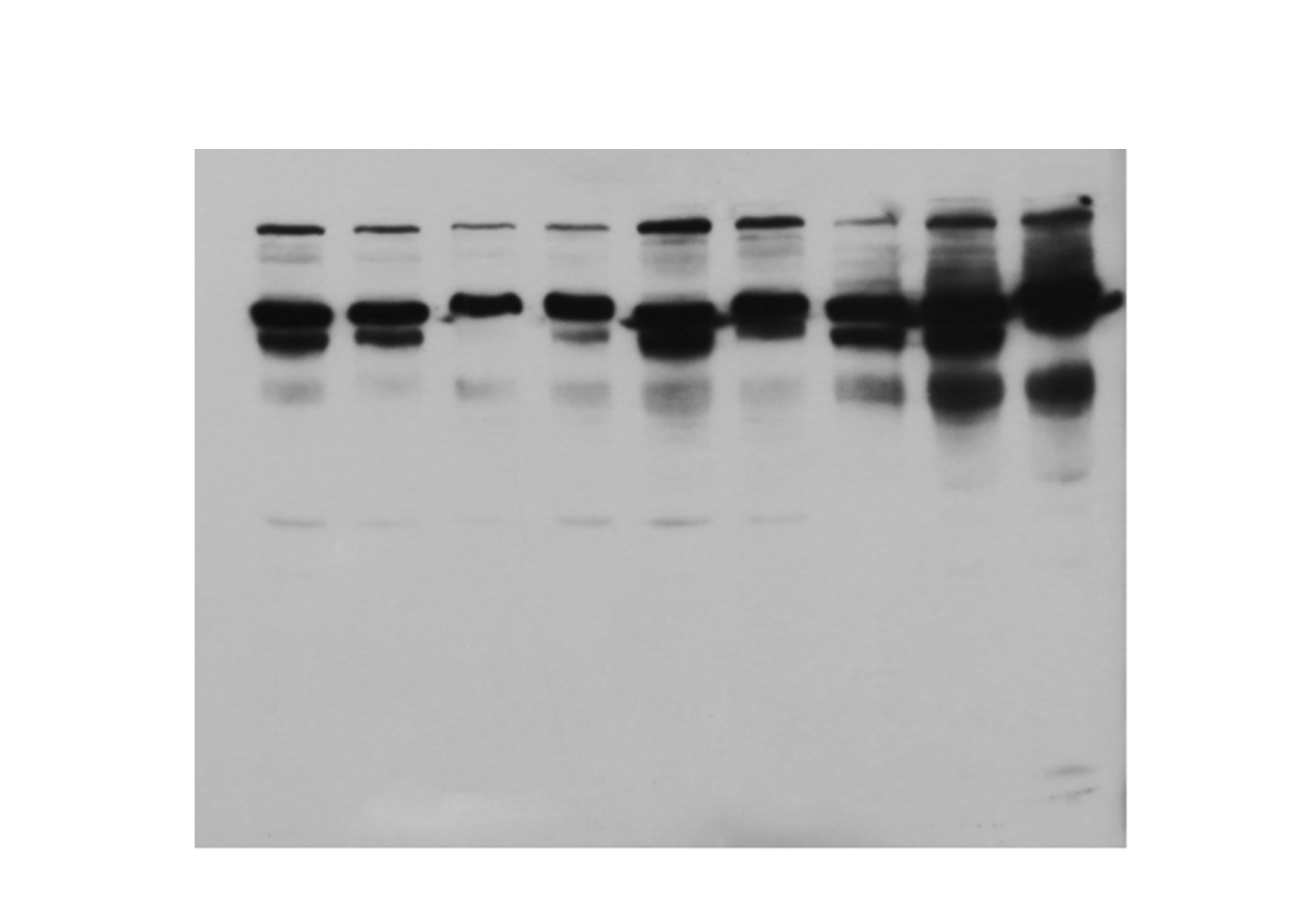

Supplement: Figure 2—source data 2. [file elife-97511-fig2-data2.zip › Figure2-source data2/WB of Figure2D.tif]

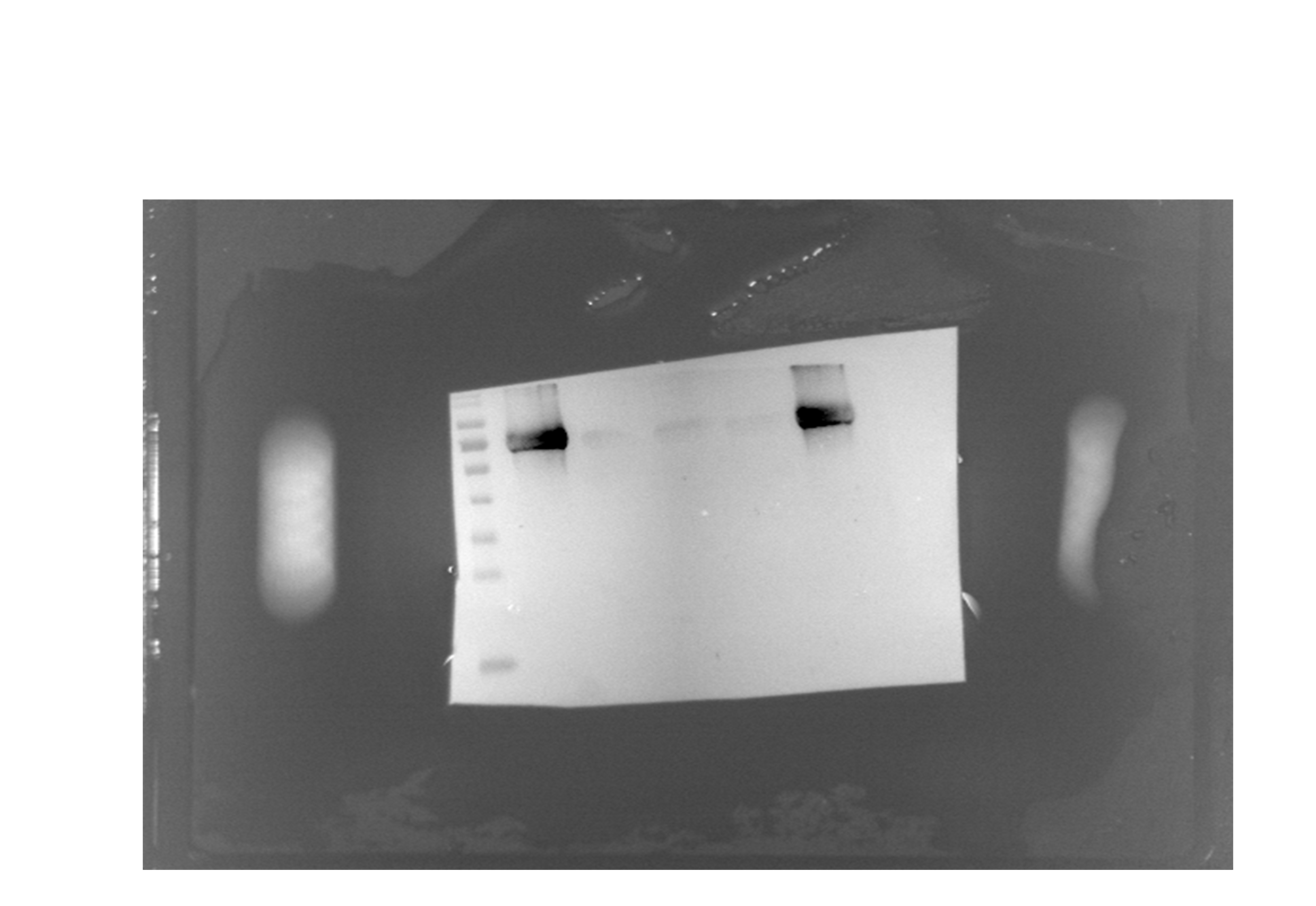

Supplement: Figure 2—source data 2. [file elife-97511-fig2-data2.zip › Figure2-source data2/WB of Figure2E.tif]

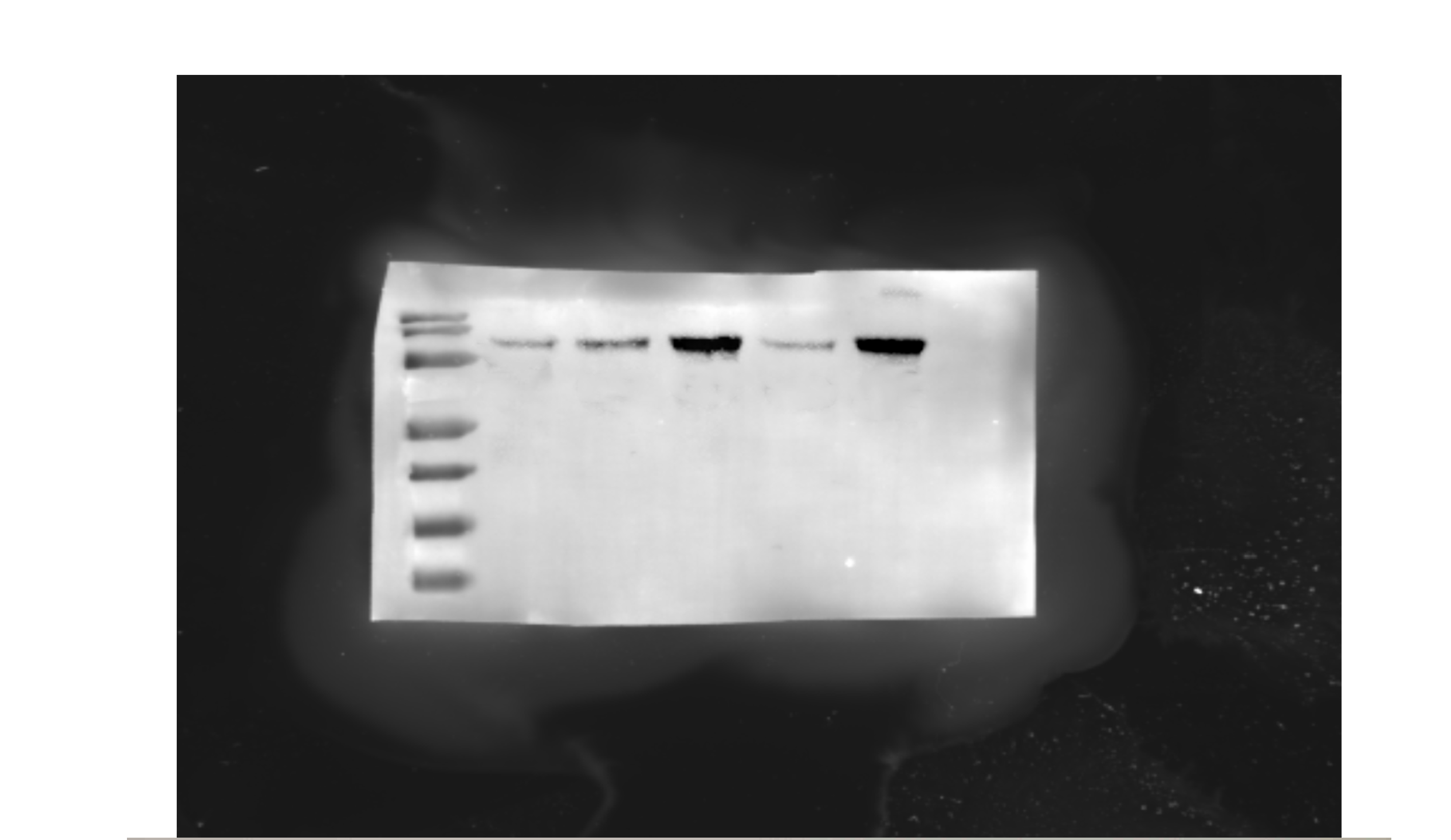

Supplement: Figure 2—source data 2. [file elife-97511-fig2-data2.zip › Figure2-source data2/WB of Figure2F.tif]

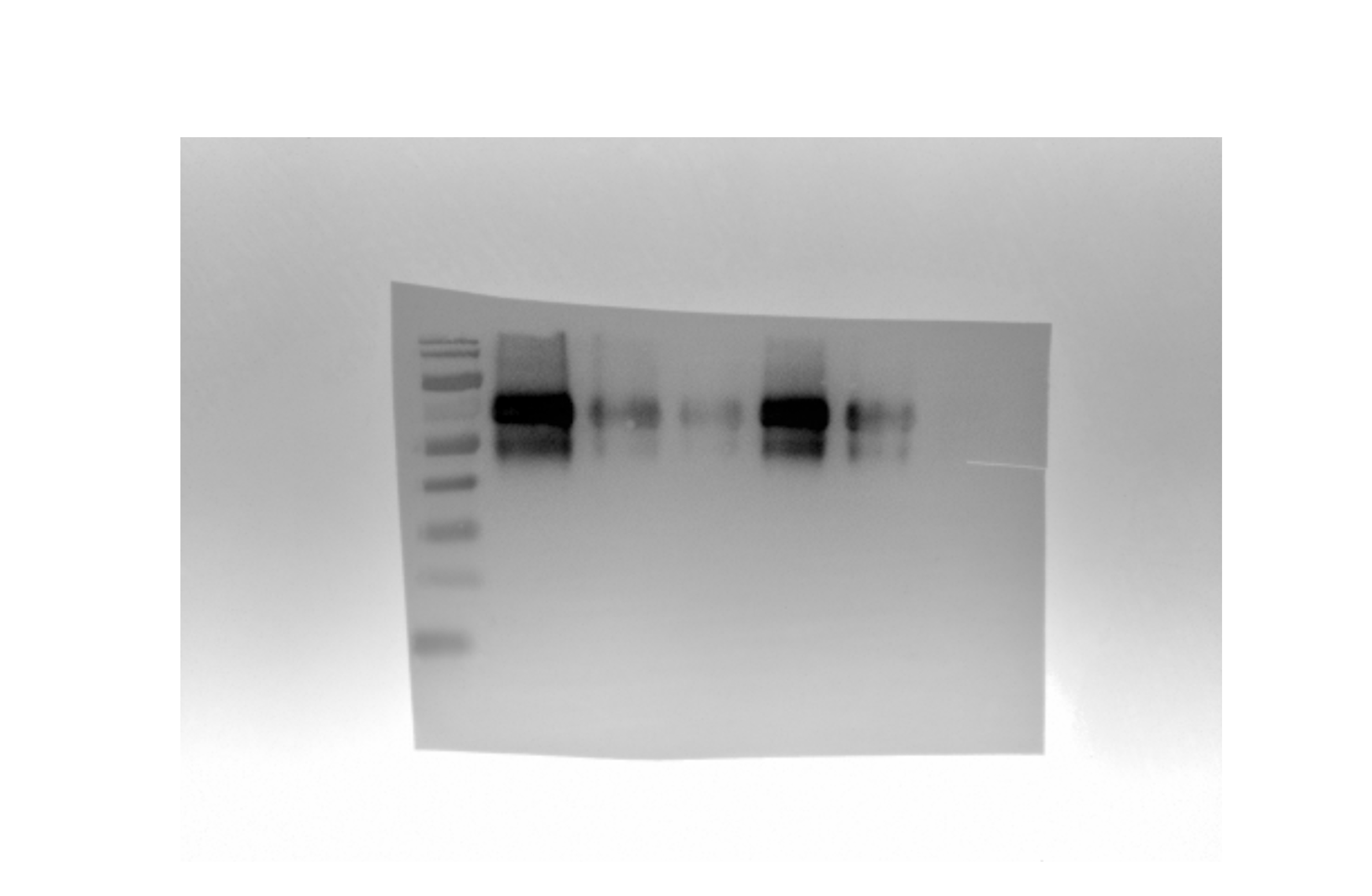

Supplement: Figure 2—source data 2. [file elife-97511-fig2-data2.zip › Figure2-source data2/WB of Figure2G.tif]

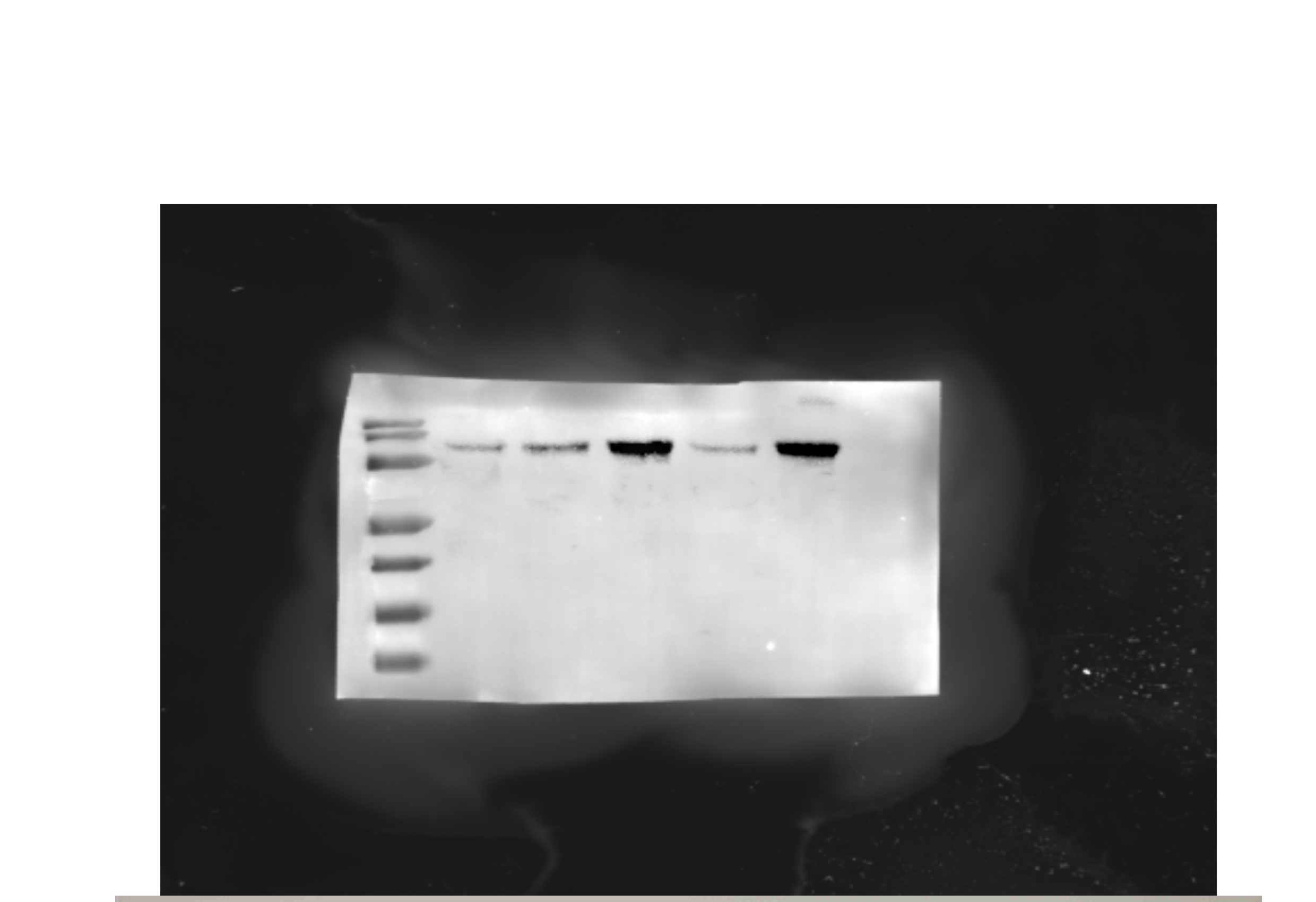

Supplement: Figure 2—source data 2. [file elife-97511-fig2-data2.zip › Figure2-source data2/WB of Figure2H.tif]

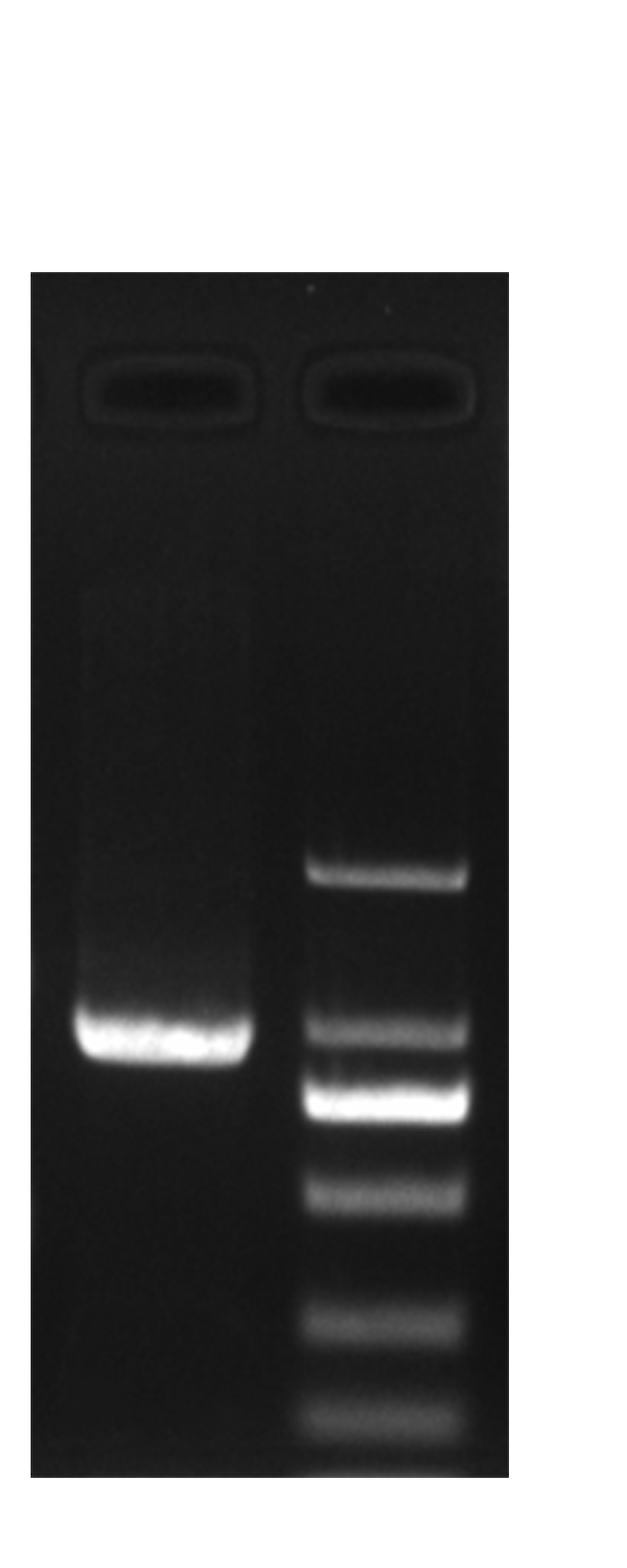

Supplement: Figure 2—figure supplement 2—source data 2. [file elife-97511-fig2-figsupp2-data2.zip › Figure 2-figure supplement 2-source data 2/PCR of ahacuC.tif]

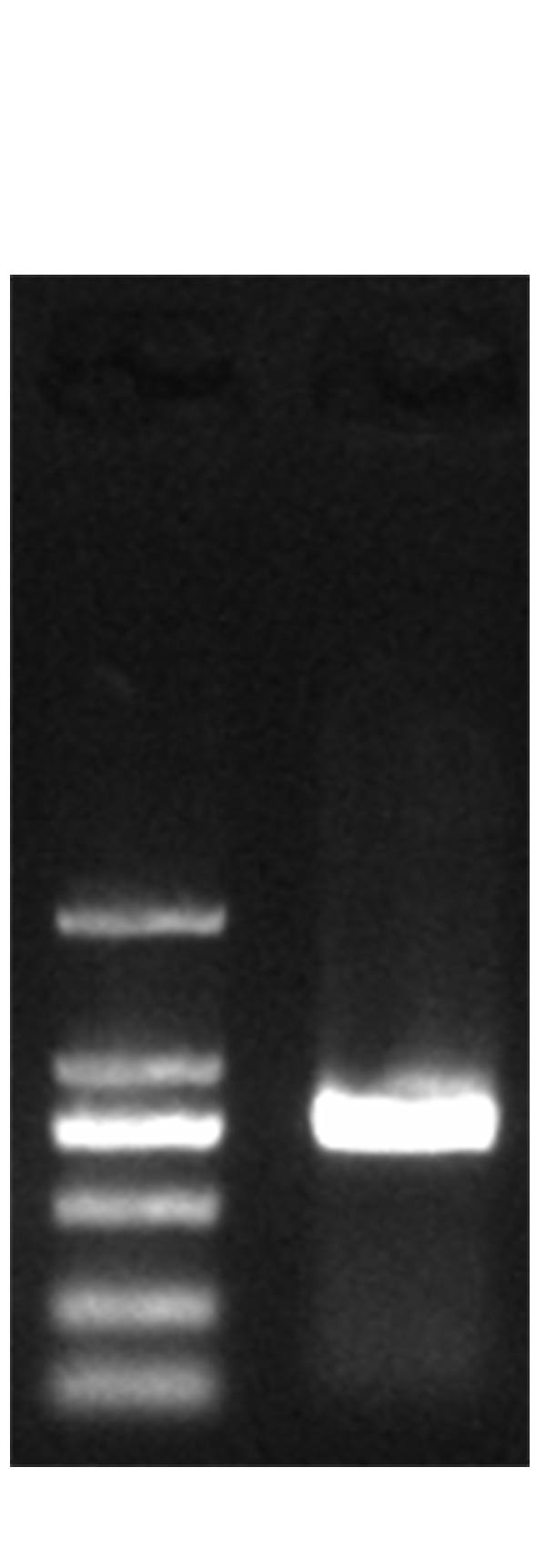

Supplement: Figure 2—figure supplement 2—source data 2. [file elife-97511-fig2-figsupp2-data2.zip › Figure 2-figure supplement 2-source data 2/PCR of ahcobB.tif]

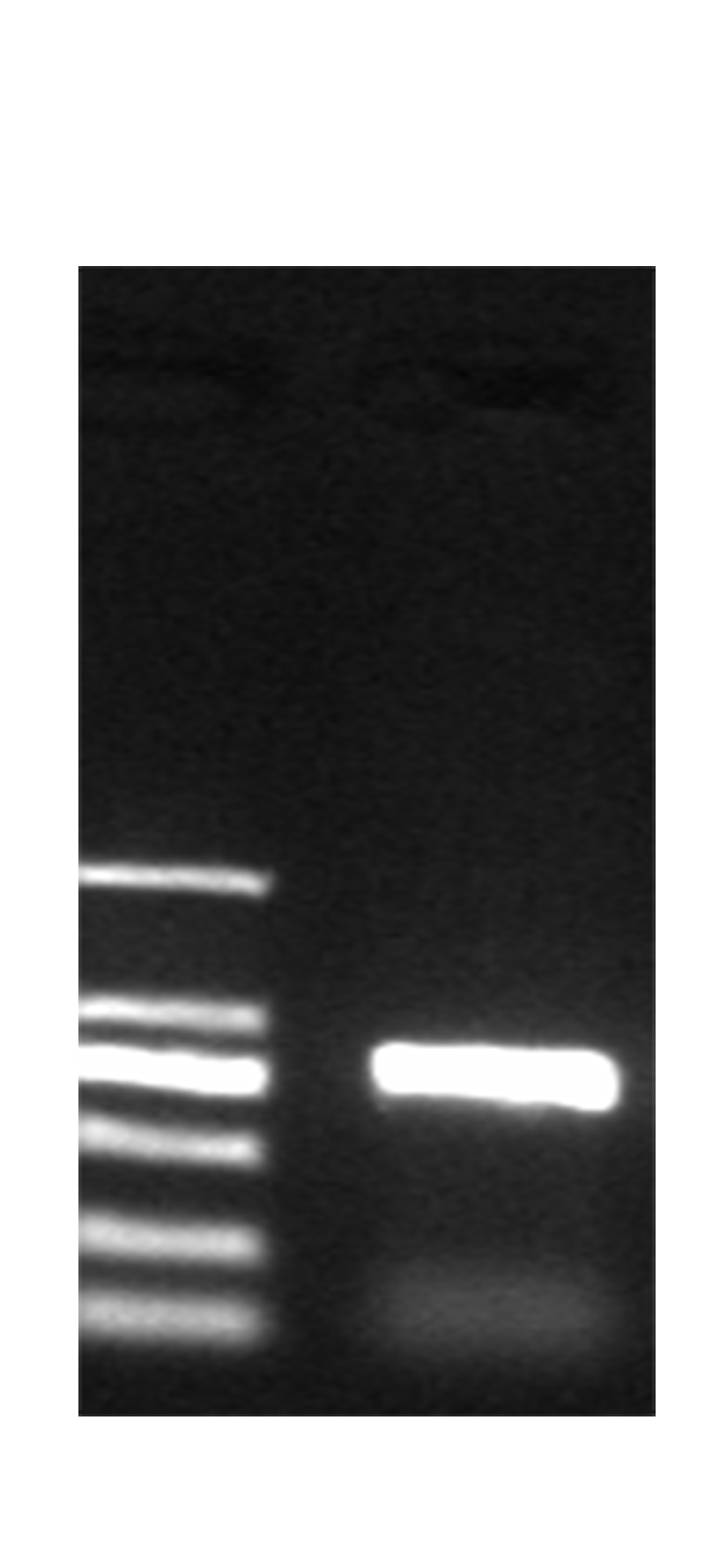

Supplement: Figure 2—figure supplement 2—source data 2. [file elife-97511-fig2-figsupp2-data2.zip › Figure 2-figure supplement 2-source data 2/PCR of ahcobQ.tif]

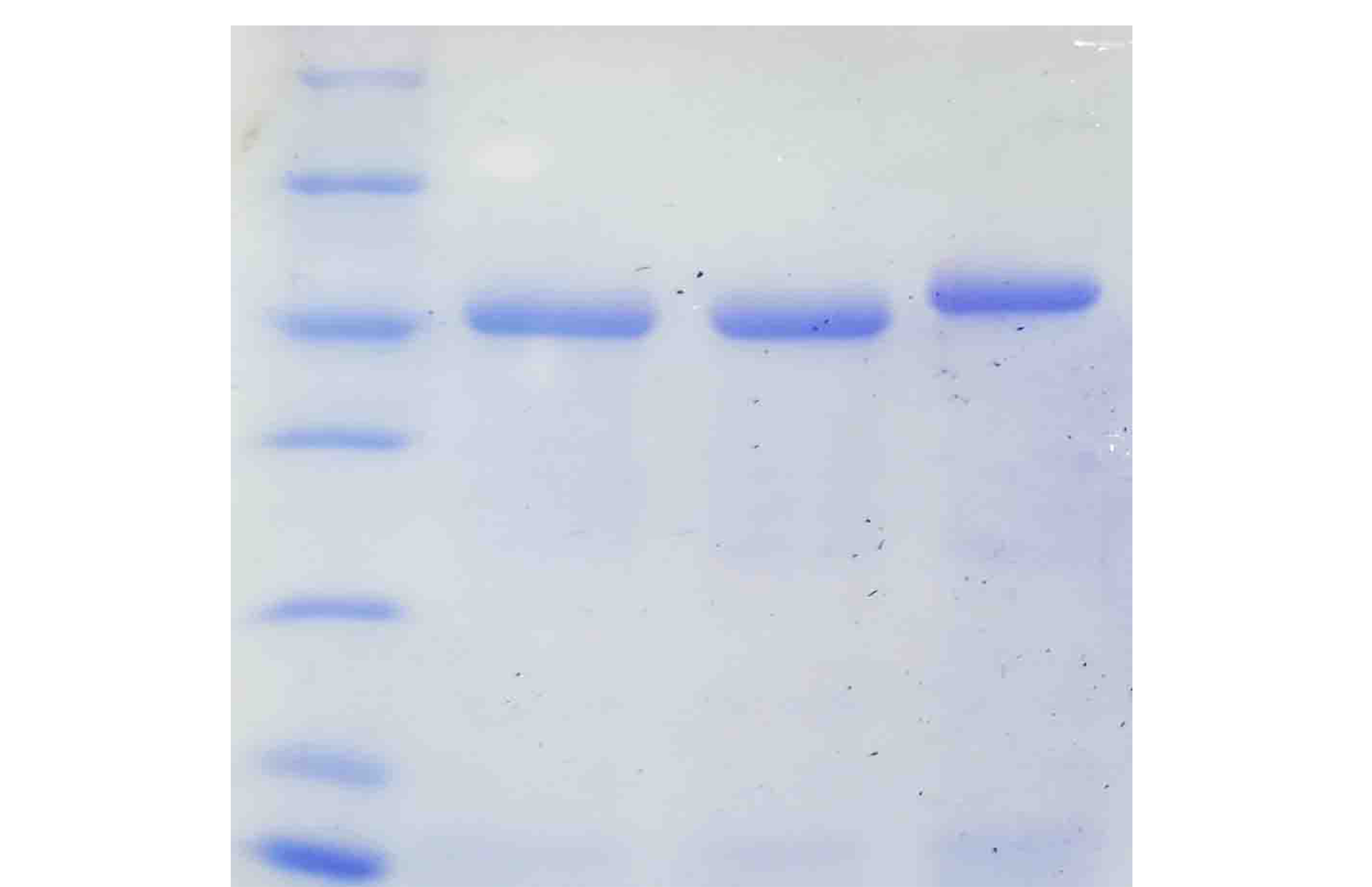

Supplement: Figure 2—figure supplement 2—source data 2. [file elife-97511-fig2-figsupp2-data2.zip › Figure 2-figure supplement 2-source data 2/SDS-PAGE of Figure 2–figure supplement 2B.tif]

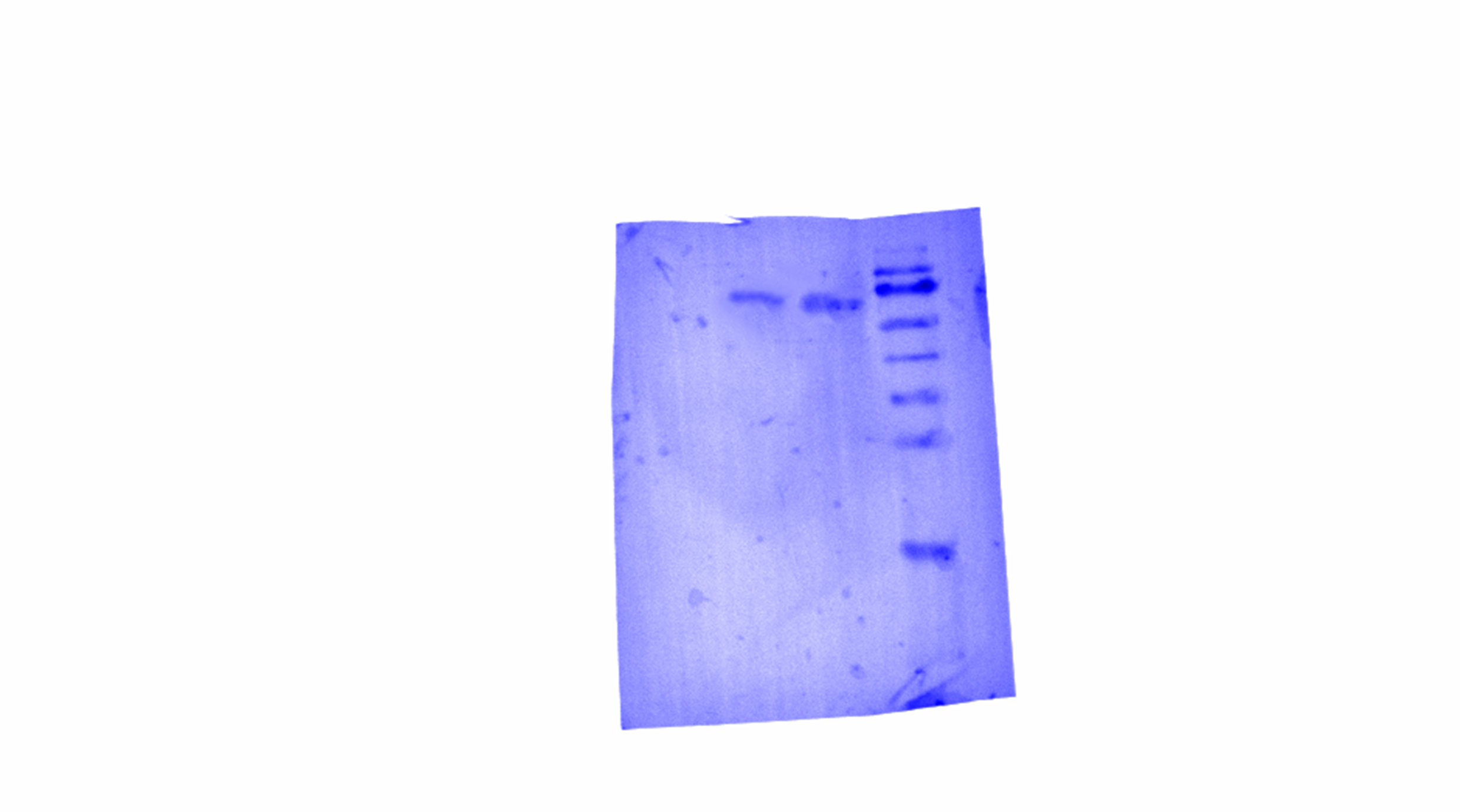

Supplement: Figure 2—figure supplement 3—source data 2. [file elife-97511-fig2-figsupp3-data2.zip › Figure 2-figure supplement 3-source data 2/loading control of Figure 2–figure supplement 3.tif]

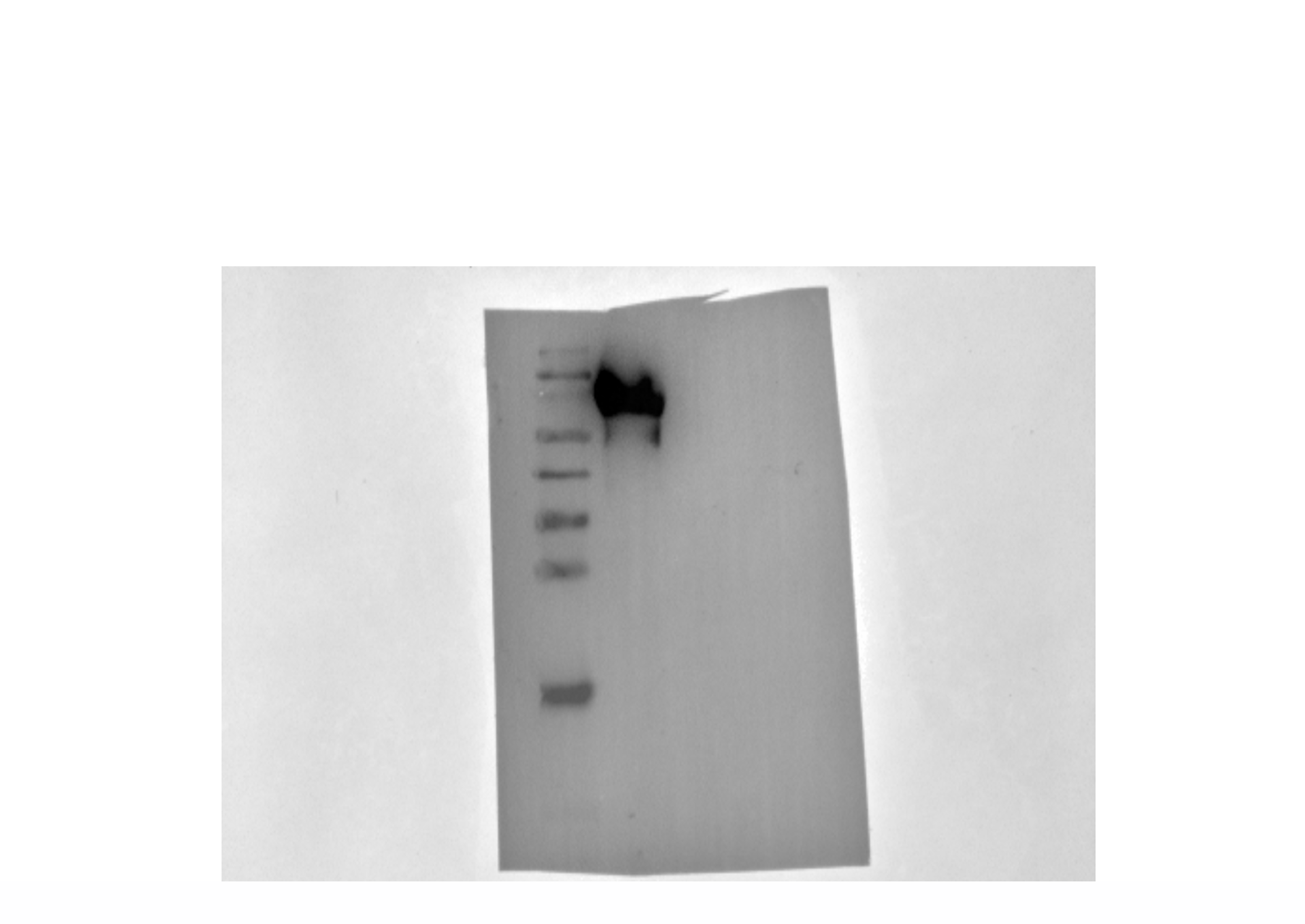

Supplement: Figure 2—figure supplement 3—source data 2. [file elife-97511-fig2-figsupp3-data2.zip › Figure 2-figure supplement 3-source data 2/WB of Figure 2–figure supplement 3.tif]

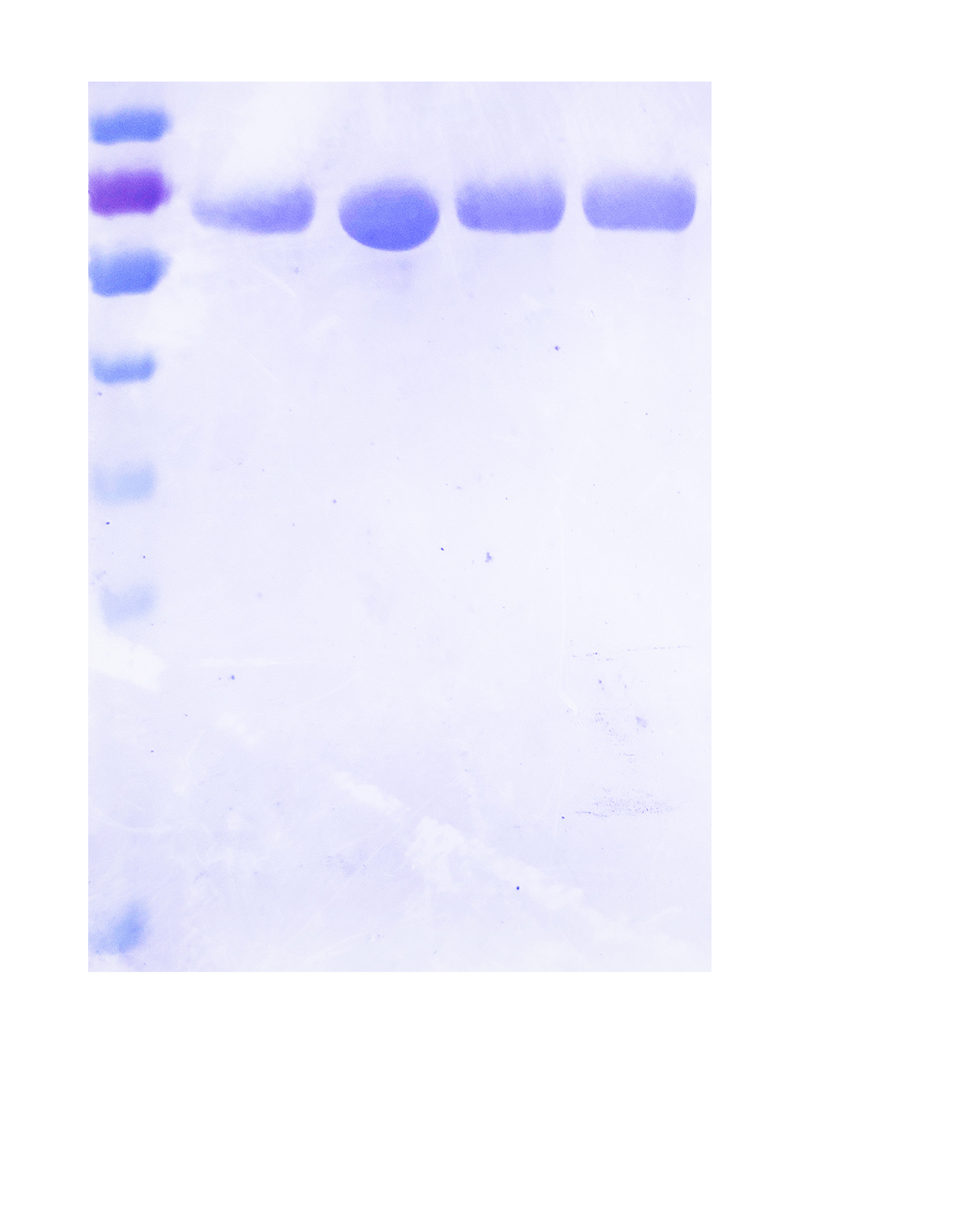

Supplement: Figure 2—figure supplement 4—source data 2. [file elife-97511-fig2-figsupp4-data2.zip › Figure 2-figure supplement 4-source data 2/loading control of GST.tif]

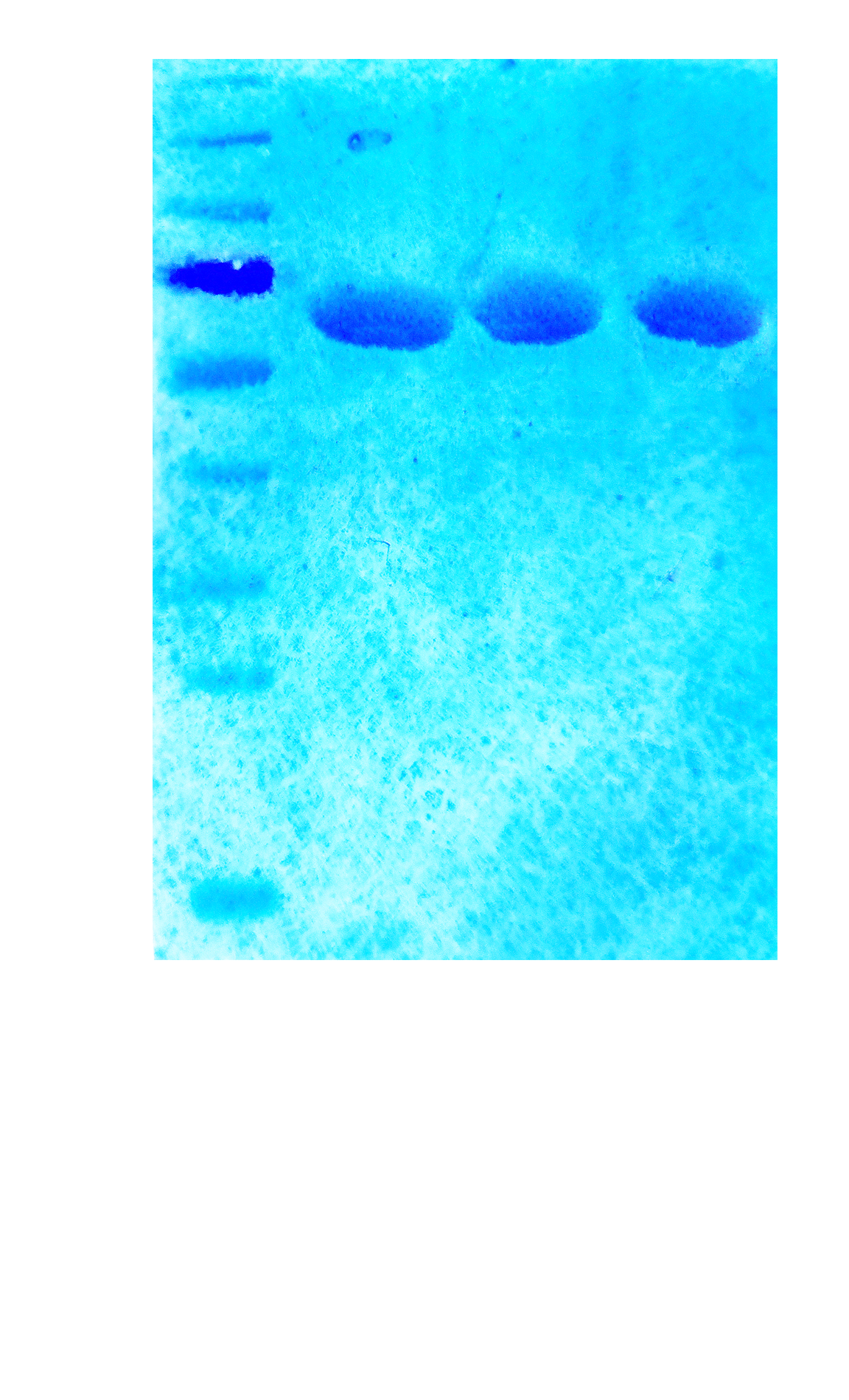

Supplement: Figure 2—figure supplement 4—source data 2. [file elife-97511-fig2-figsupp4-data2.zip › Figure 2-figure supplement 4-source data 2/loading control of HIS.tif]

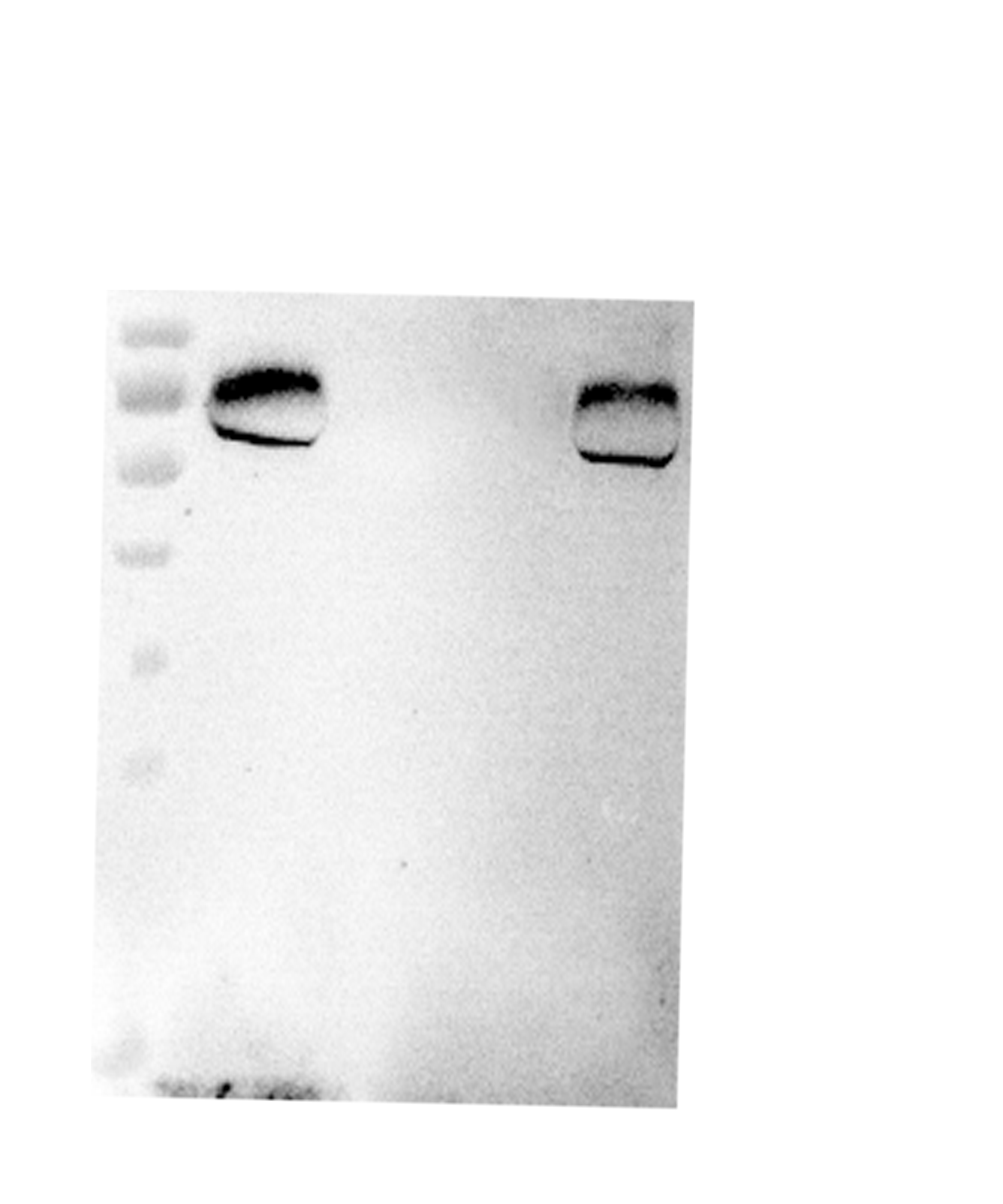

Supplement: Figure 2—figure supplement 4—source data 2. [file elife-97511-fig2-figsupp4-data2.zip › Figure 2-figure supplement 4-source data 2/WB of GST.tif]

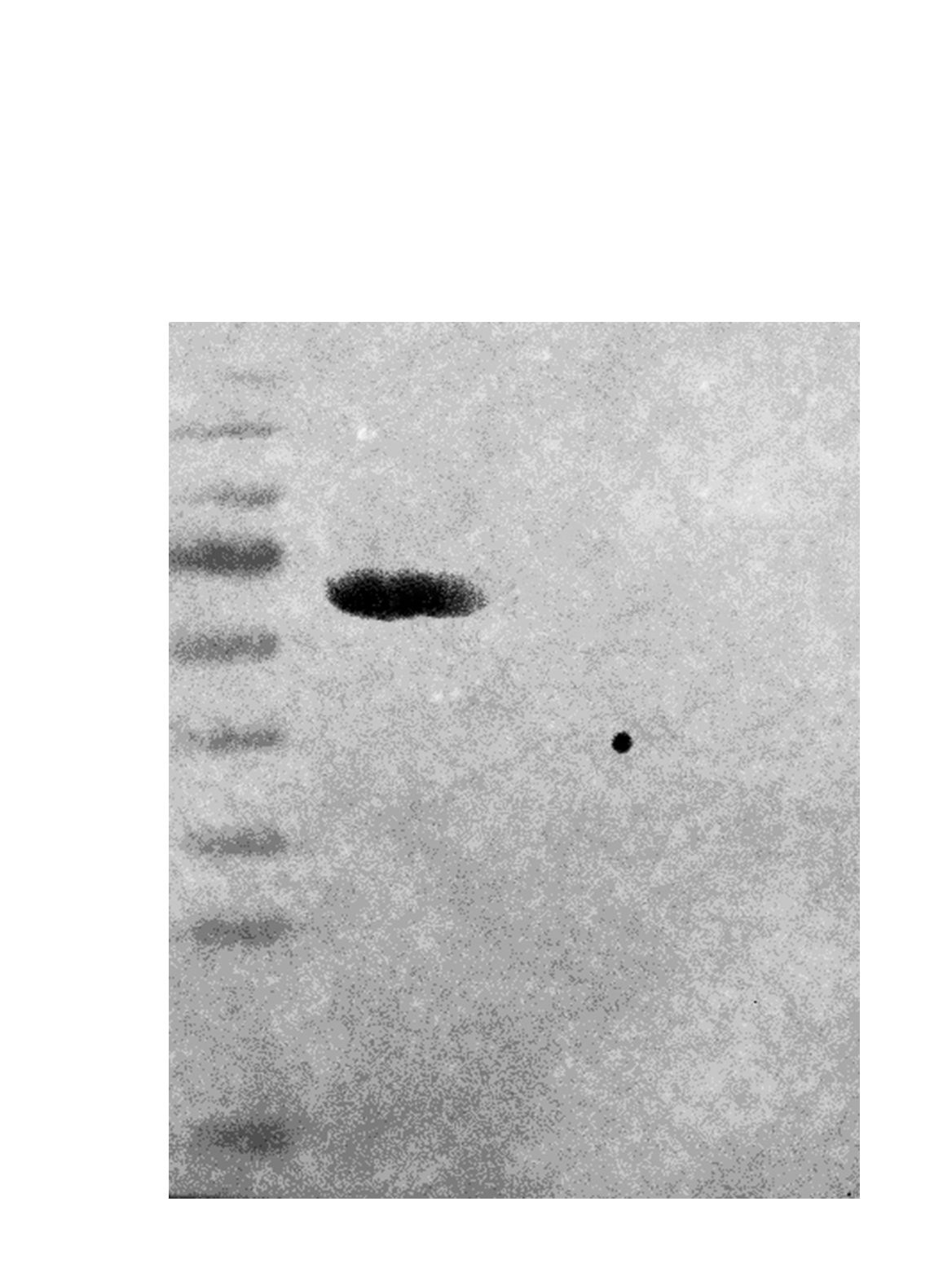

Supplement: Figure 2—figure supplement 4—source data 2. [file elife-97511-fig2-figsupp4-data2.zip › Figure 2-figure supplement 4-source data 2/WB of HIS.tif]

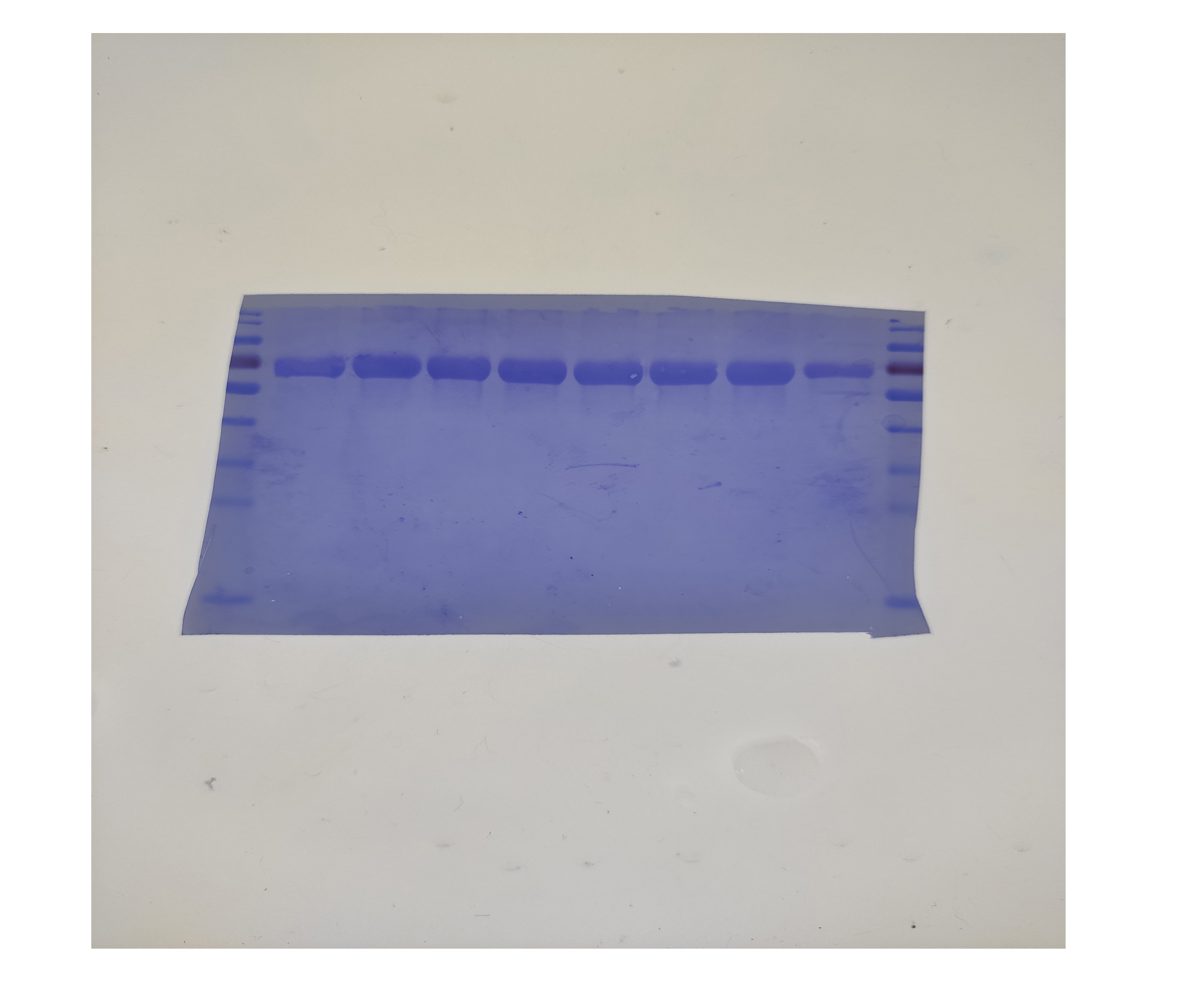

Supplement: Figure 2—figure supplement 5—source data 2. [file elife-97511-fig2-figsupp5-data2.zip › Figure 2-figure supplement 5-source data 2/loading control of Figure 2–figure supplement 5.tif]

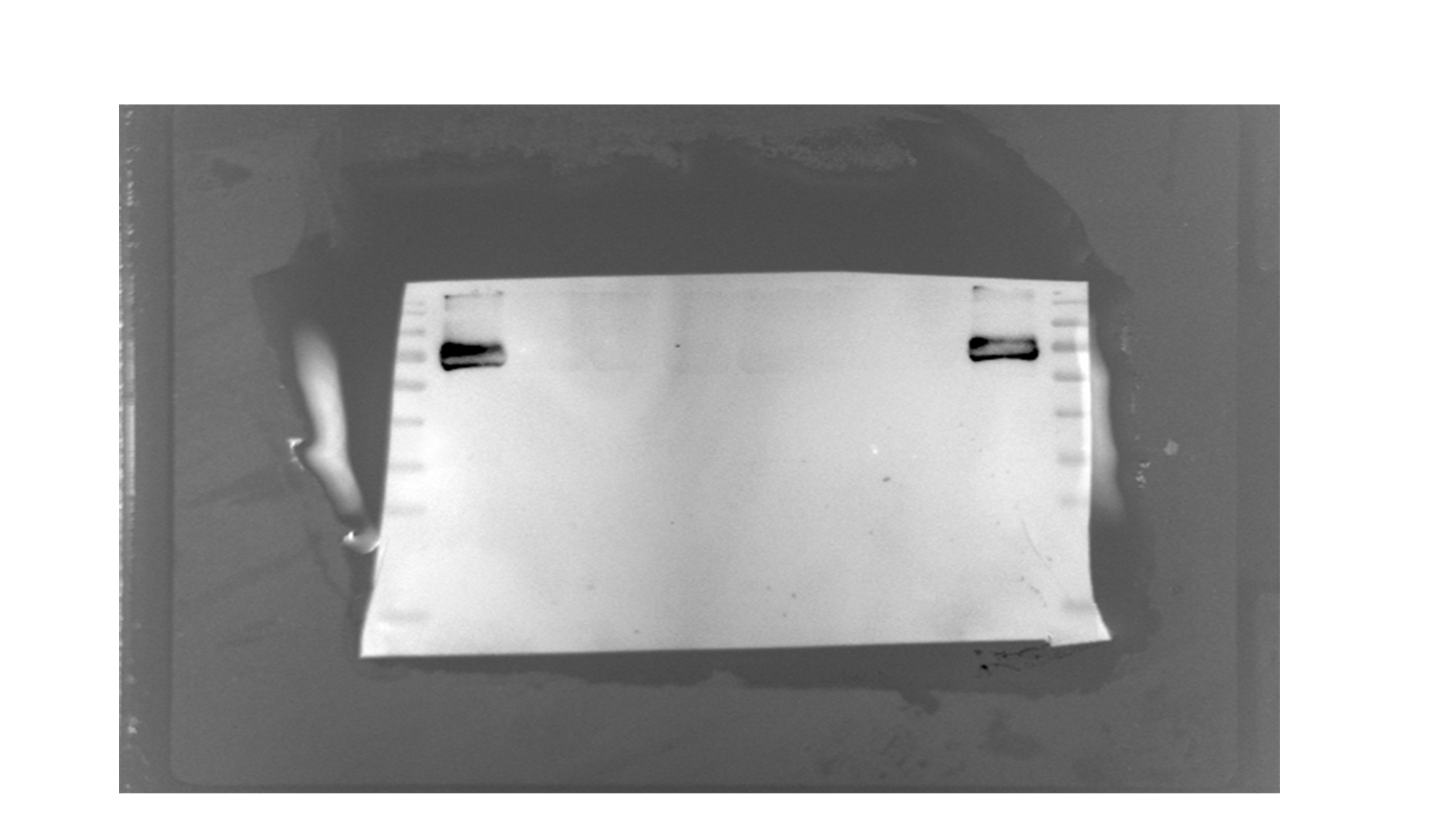

Supplement: Figure 2—figure supplement 5—source data 2. [file elife-97511-fig2-figsupp5-data2.zip › Figure 2-figure supplement 5-source data 2/WB of Figure 2–figure supplement 5.tif]

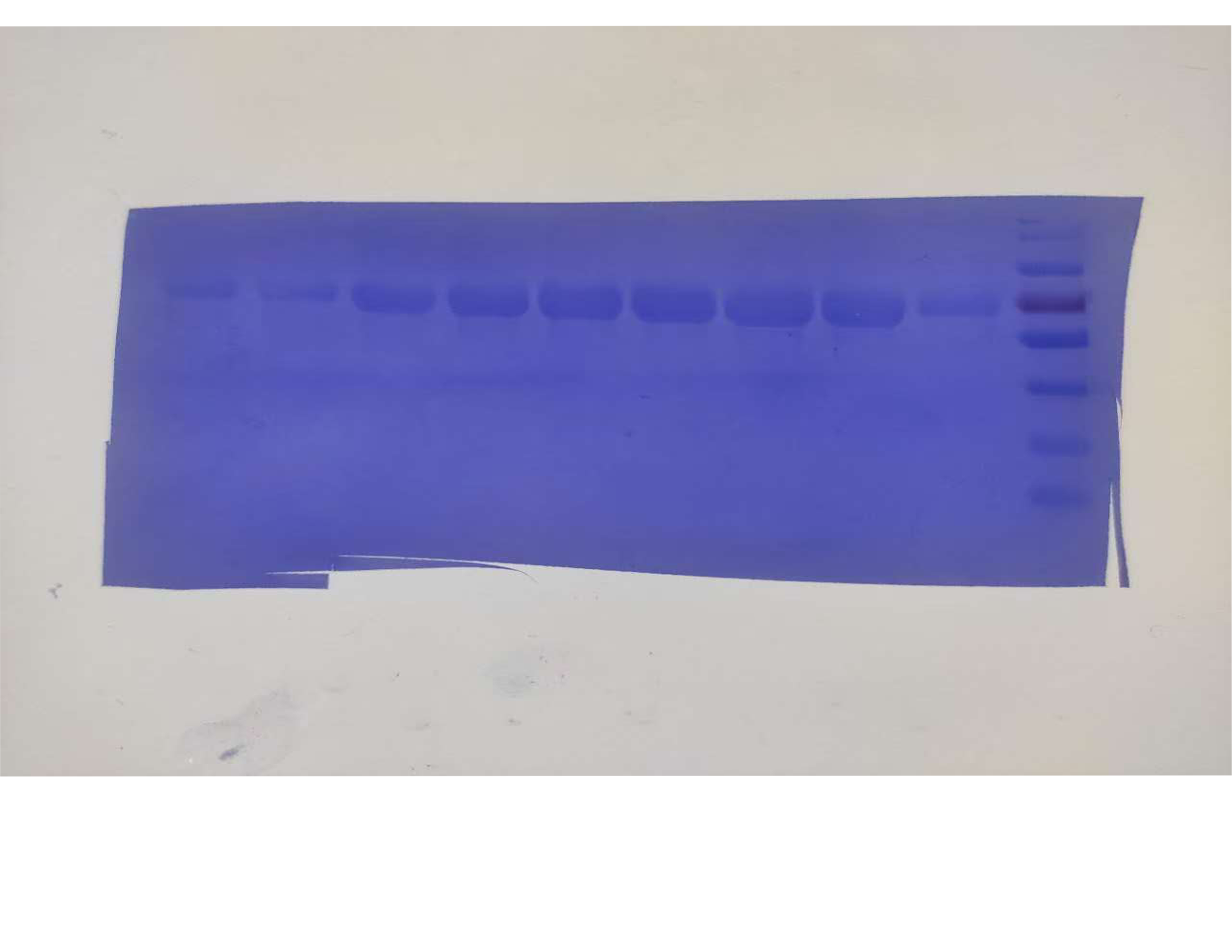

Supplement: Figure 4—source data 2. [file elife-97511-fig4-data2.zip › Figure4-source data2/loading conrtol of Figure4A.tif]

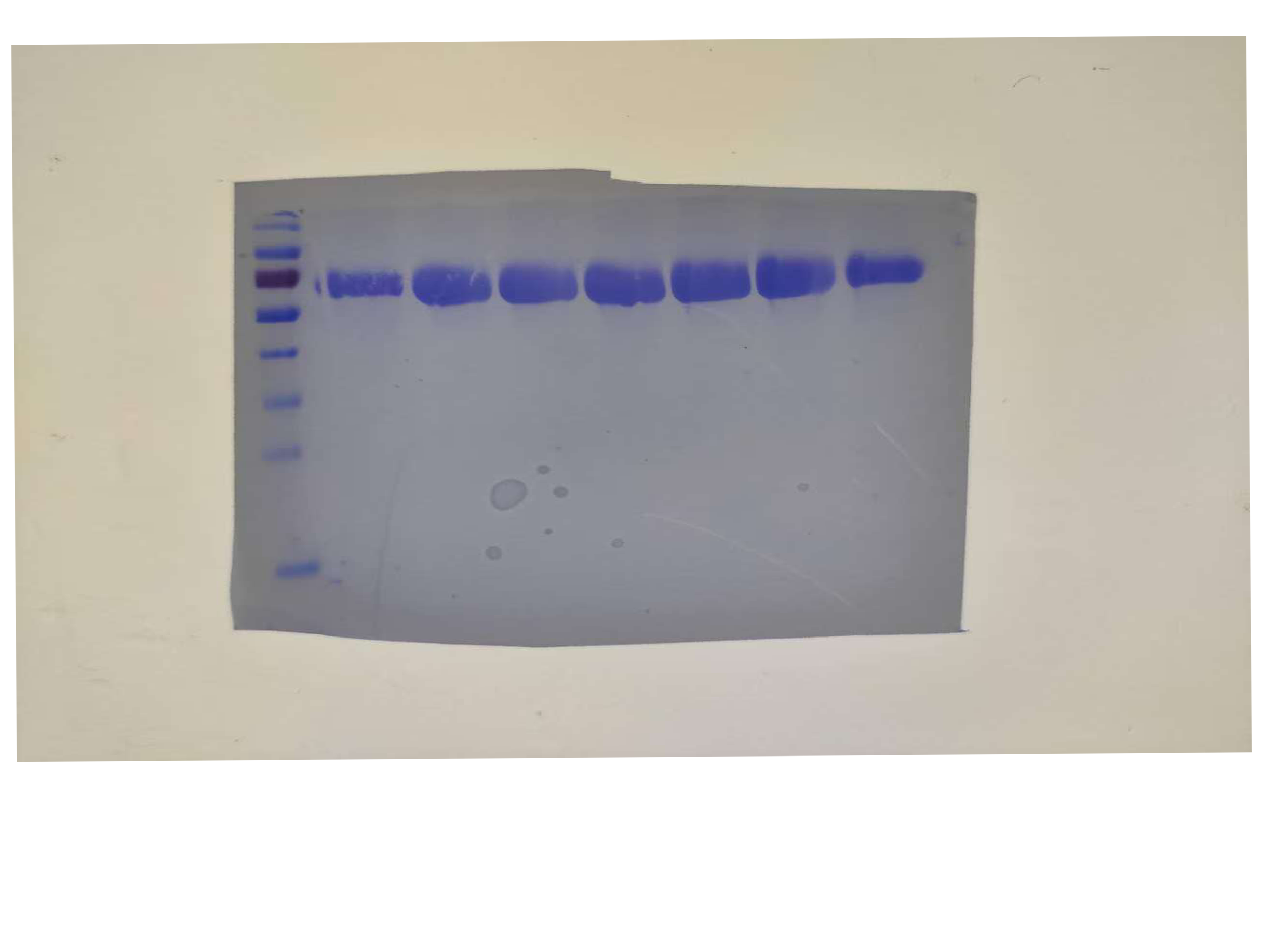

Supplement: Figure 4—source data 2. [file elife-97511-fig4-data2.zip › Figure4-source data2/loading conrtol of Figure4C.tif]

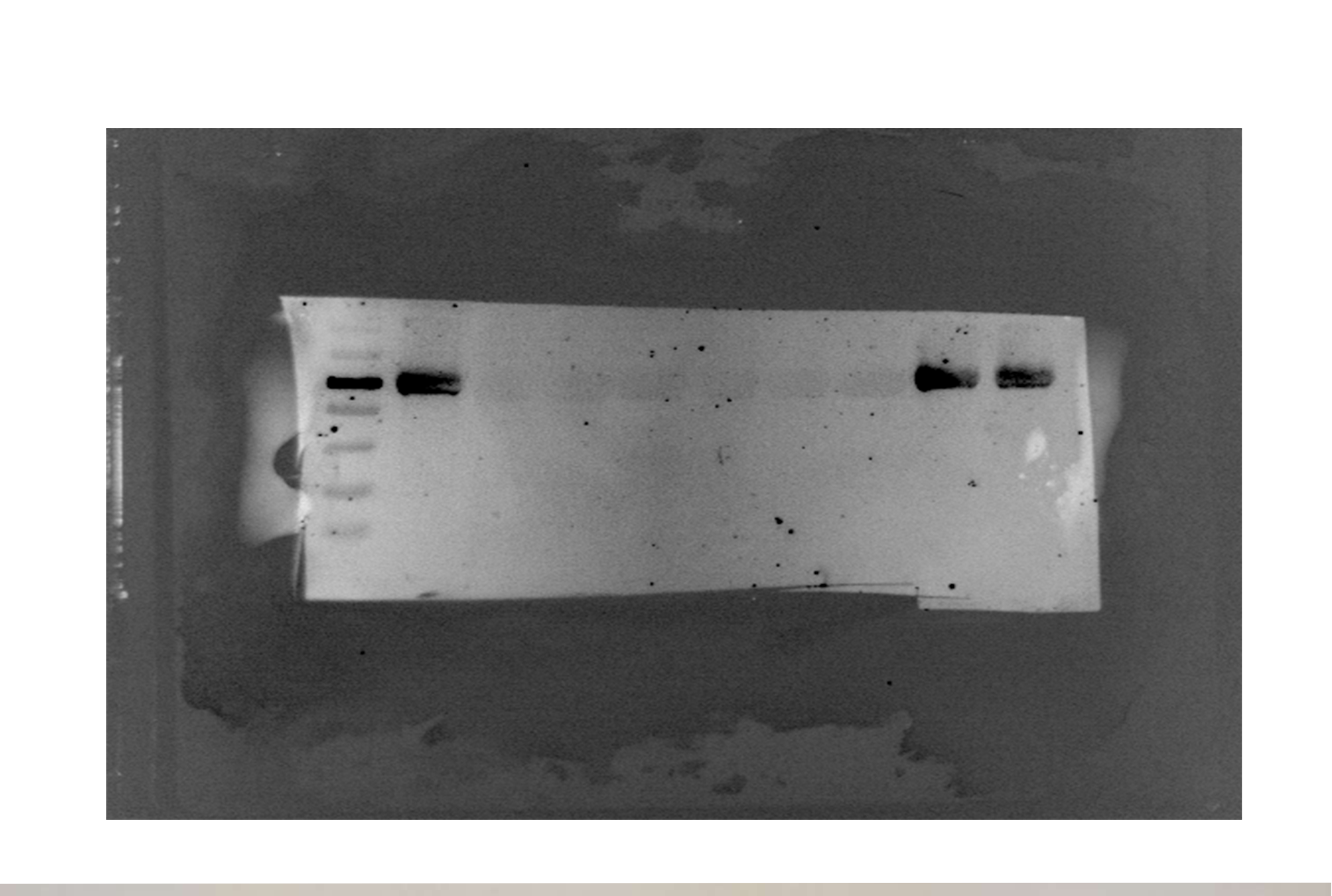

Supplement: Figure 4—source data 2. [file elife-97511-fig4-data2.zip › Figure4-source data2/WB of Figure4A.tif]

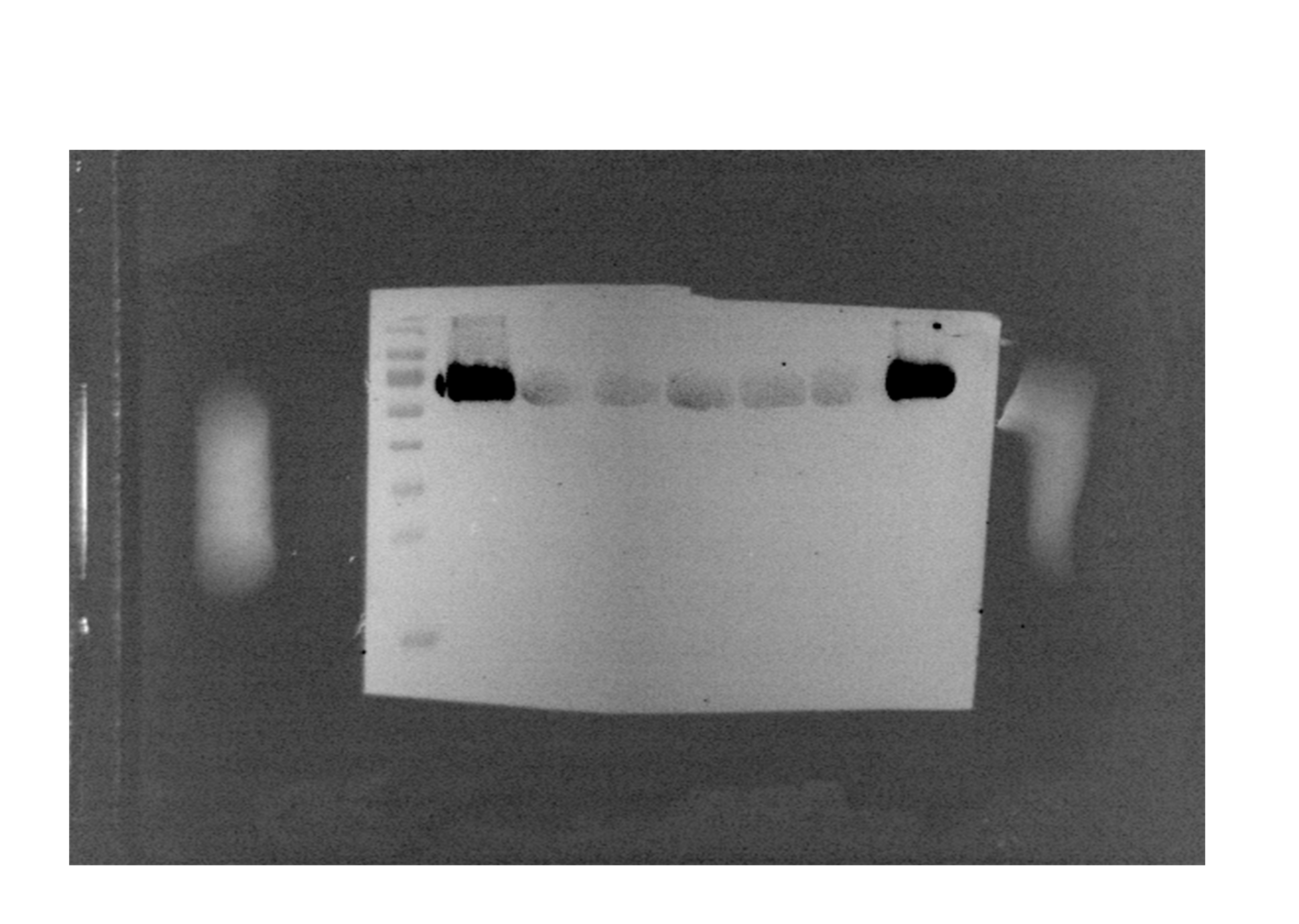

Supplement: Figure 4—source data 2. [file elife-97511-fig4-data2.zip › Figure4-source data2/WB of Figure4C.tif]

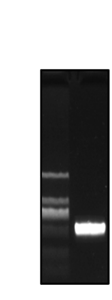

Supplement: Figure 4—figure supplement 1—source data 2. [file elife-97511-fig4-figsupp1-data2.zip › Figure 4–figure supplement 1-source data 2/PCR of lane 1.tif]

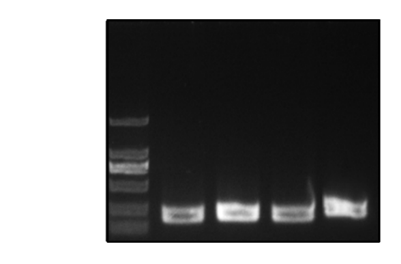

Supplement: Figure 4—figure supplement 1—source data 2. [file elife-97511-fig4-figsupp1-data2.zip › Figure 4–figure supplement 1-source data 2/PCR of lane 10-13.tif]

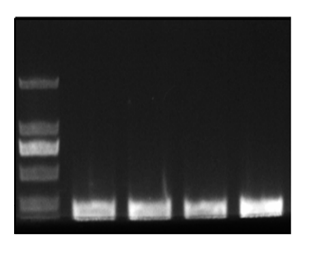

Supplement: Figure 4—figure supplement 1—source data 2. [file elife-97511-fig4-figsupp1-data2.zip › Figure 4–figure supplement 1-source data 2/PCR of lane 14-17.tif]

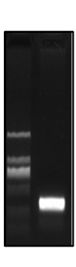

Supplement: Figure 4—figure supplement 1—source data 2. [file elife-97511-fig4-figsupp1-data2.zip › Figure 4–figure supplement 1-source data 2/PCR of lane 2.tif]

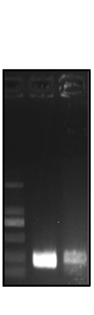

Supplement: Figure 4—figure supplement 1—source data 2. [file elife-97511-fig4-figsupp1-data2.zip › Figure 4–figure supplement 1-source data 2/PCR of lane 3,4.tif]

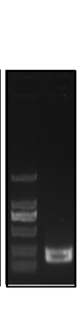

Supplement: Figure 4—figure supplement 1—source data 2. [file elife-97511-fig4-figsupp1-data2.zip › Figure 4–figure supplement 1-source data 2/PCR of lane 5.tif]

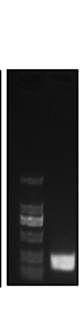

Supplement: Figure 4—figure supplement 1—source data 2. [file elife-97511-fig4-figsupp1-data2.zip › Figure 4–figure supplement 1-source data 2/PCR of lane 6.tif]

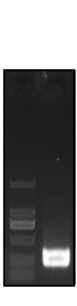

Supplement: Figure 4—figure supplement 1—source data 2. [file elife-97511-fig4-figsupp1-data2.zip › Figure 4–figure supplement 1-source data 2/PCR of lane 7.tif]

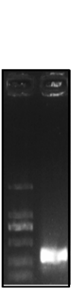

Supplement: Figure 4—figure supplement 1—source data 2. [file elife-97511-fig4-figsupp1-data2.zip › Figure 4–figure supplement 1-source data 2/PCR of lane 8.tif]

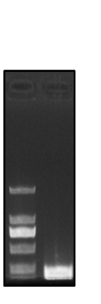

Supplement: Figure 4—figure supplement 1—source data 2. [file elife-97511-fig4-figsupp1-data2.zip › Figure 4–figure supplement 1-source data 2/PCR of lane 9.tif]

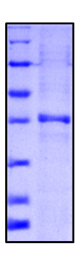

Supplement: Figure 4—figure supplement 1—source data 2. [file elife-97511-fig4-figsupp1-data2.zip › Figure 4–figure supplement 1-source data 2/SDS-PSGE of lane 1.tif]

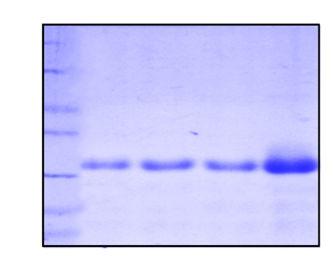

Supplement: Figure 4—figure supplement 1—source data 2. [file elife-97511-fig4-figsupp1-data2.zip › Figure 4–figure supplement 1-source data 2/SDS-PSGE of lane 10-13.tif]

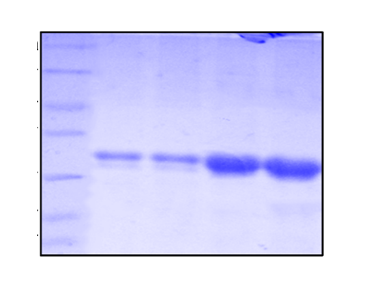

Supplement: Figure 4—figure supplement 1—source data 2. [file elife-97511-fig4-figsupp1-data2.zip › Figure 4–figure supplement 1-source data 2/SDS-PSGE of lane 14-17.tif]

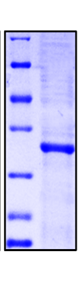

Supplement: Figure 4—figure supplement 1—source data 2. [file elife-97511-fig4-figsupp1-data2.zip › Figure 4–figure supplement 1-source data 2/SDS-PSGE of lane 2.tif]

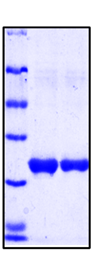

Supplement: Figure 4—figure supplement 1—source data 2. [file elife-97511-fig4-figsupp1-data2.zip › Figure 4–figure supplement 1-source data 2/SDS-PSGE of lane 3,4.tif]

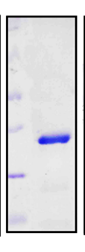

Supplement: Figure 4—figure supplement 1—source data 2. [file elife-97511-fig4-figsupp1-data2.zip › Figure 4–figure supplement 1-source data 2/SDS-PSGE of lane 5.tif]

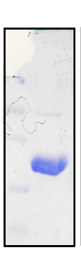

Supplement: Figure 4—figure supplement 1—source data 2. [file elife-97511-fig4-figsupp1-data2.zip › Figure 4–figure supplement 1-source data 2/SDS-PSGE of lane 6.tif]

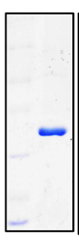

Supplement: Figure 4—figure supplement 1—source data 2. [file elife-97511-fig4-figsupp1-data2.zip › Figure 4–figure supplement 1-source data 2/SDS-PSGE of lane 7.tif]

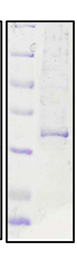

Supplement: Figure 4—figure supplement 1—source data 2. [file elife-97511-fig4-figsupp1-data2.zip › Figure 4–figure supplement 1-source data 2/SDS-PSGE of lane 8.tif]

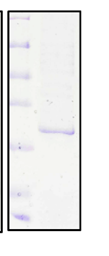

Supplement: Figure 4—figure supplement 1—source data 2. [file elife-97511-fig4-figsupp1-data2.zip › Figure 4–figure supplement 1-source data 2/SDS-PSGE of lane 9.tif]

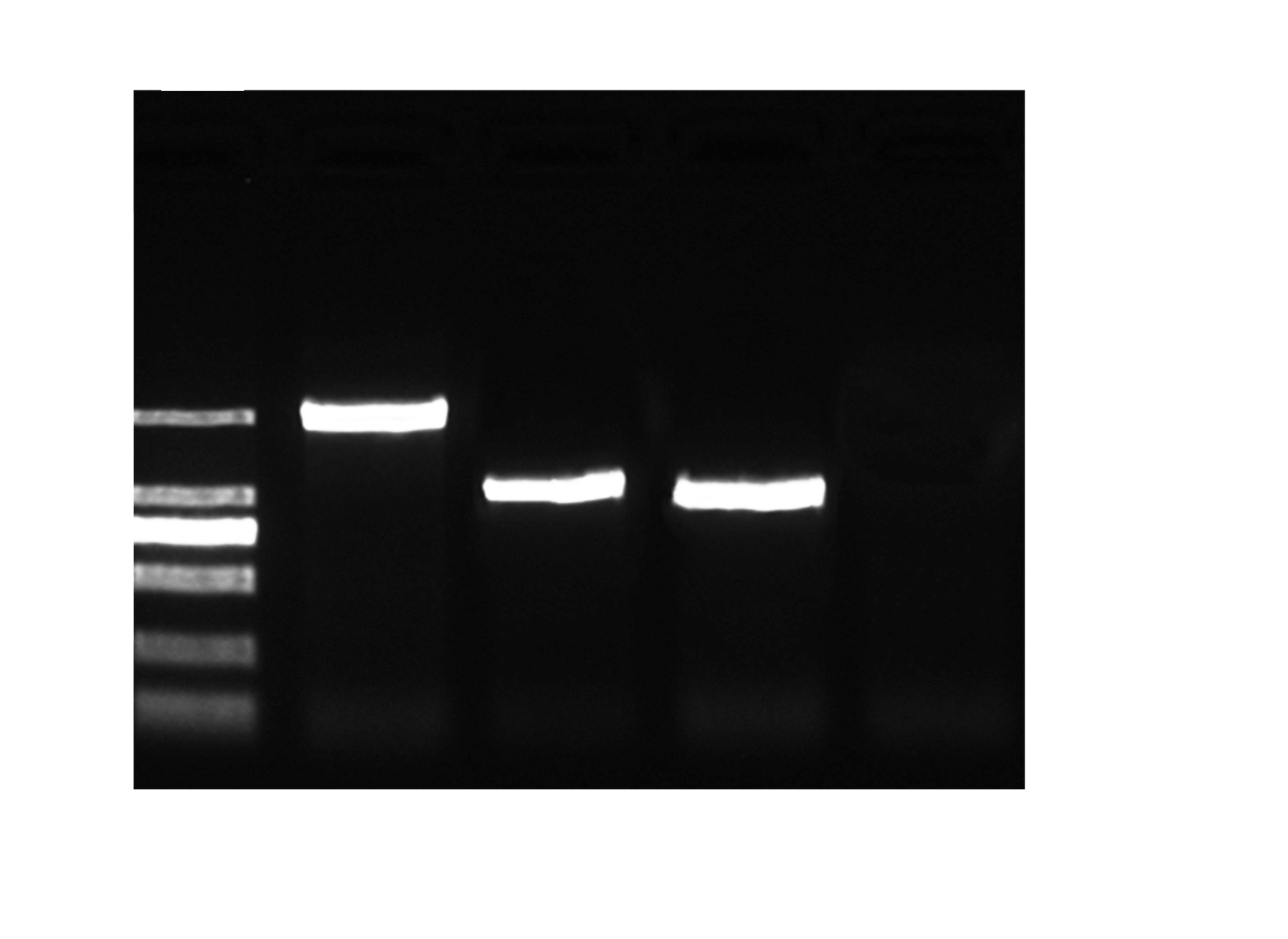

Supplement: Figure 6—figure supplement 1—source data 2. [file elife-97511-fig6-figsupp1-data2.zip › Figure 6–figure supplement 1-source data 2/Figure 6–figure supplement 1-source data 2.tif]

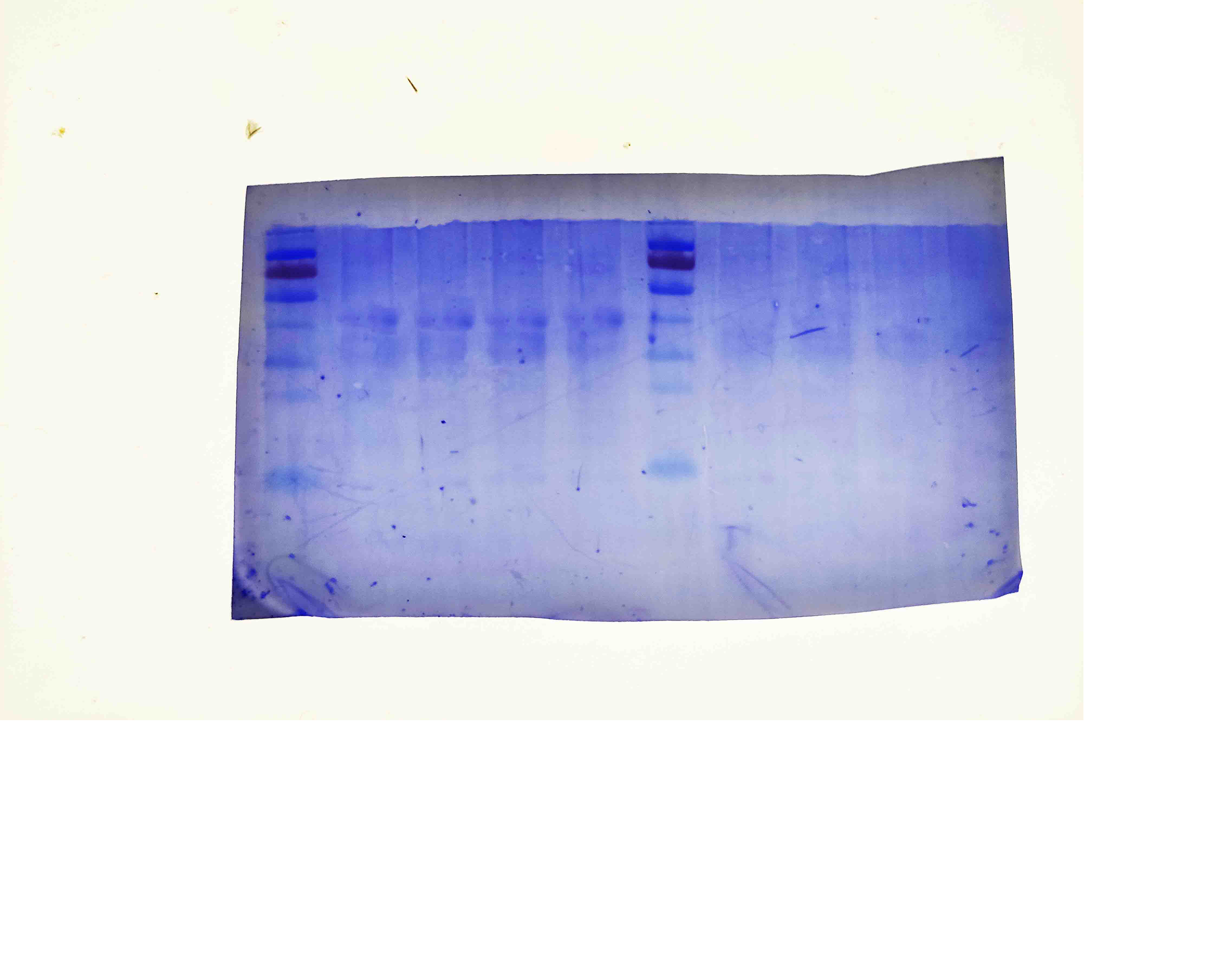

Supplement: Figure 7—source data 2. [file elife-97511-fig7-data2.zip › Figure7-source data2/loading control of ArcA-2.tif]

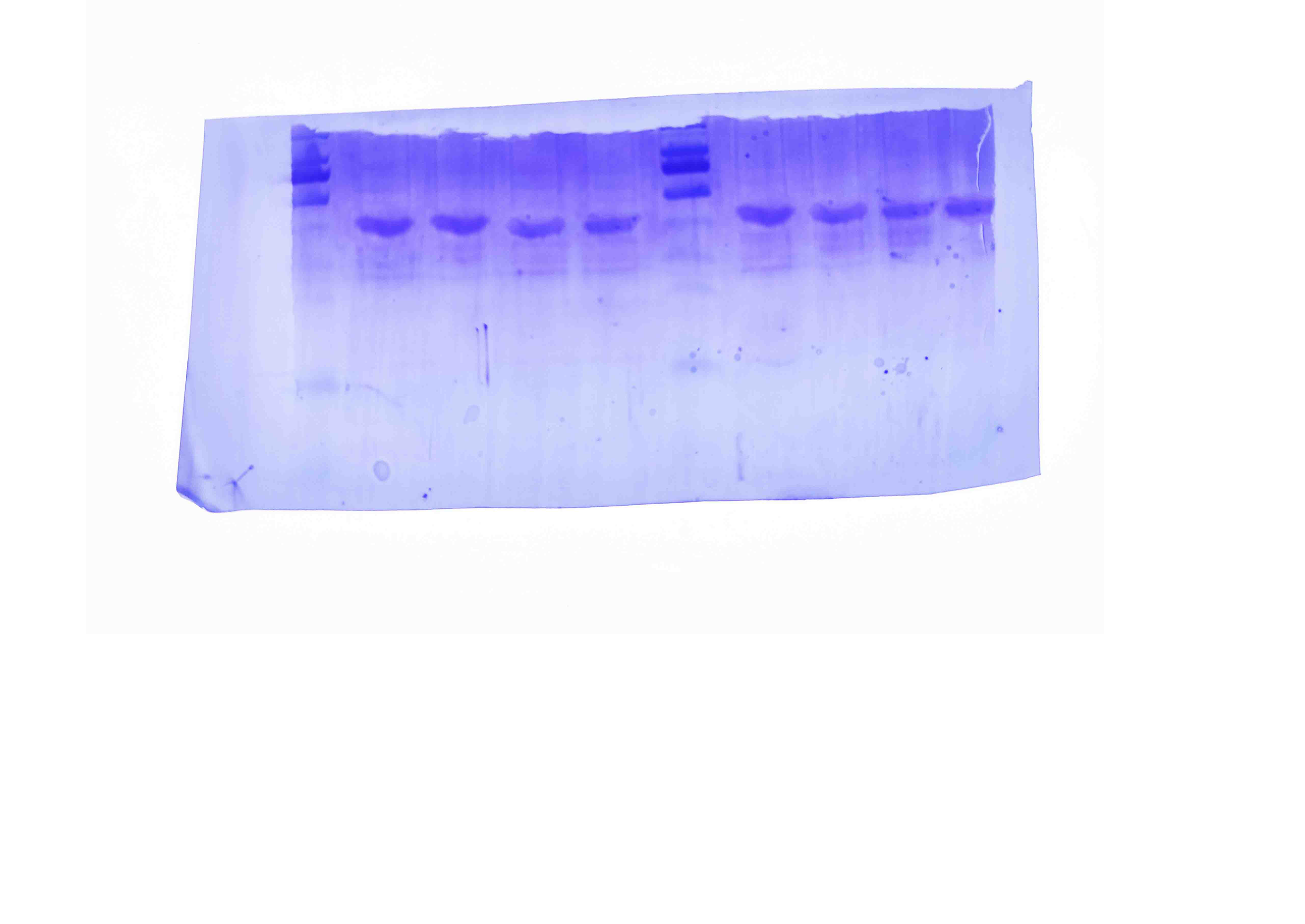

Supplement: Figure 7—source data 2. [file elife-97511-fig7-data2.zip › Figure7-source data2/loading control of ENO.tif]

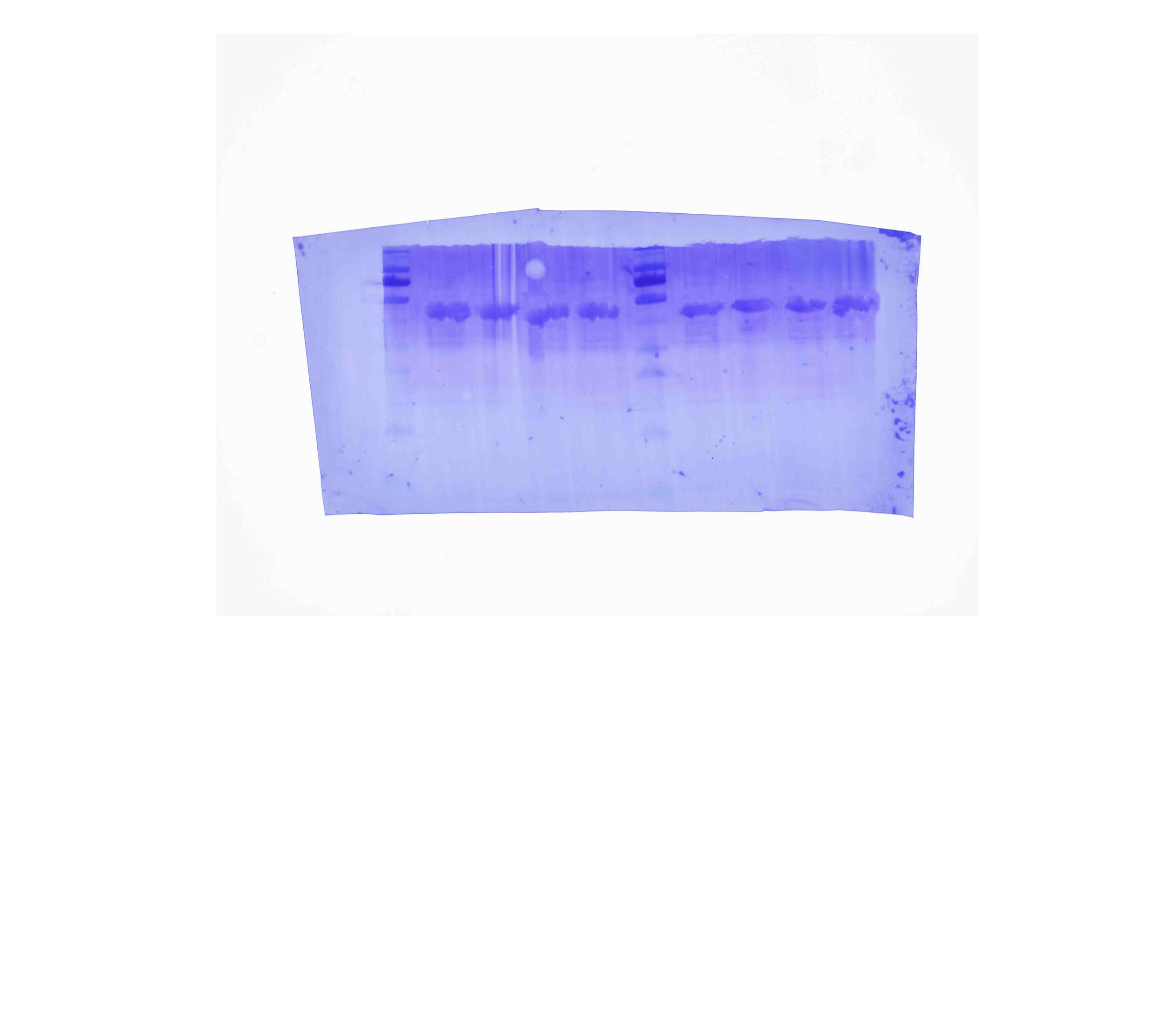

Supplement: Figure 7—source data 2. [file elife-97511-fig7-data2.zip › Figure7-source data2/loading control of SUN.tif]

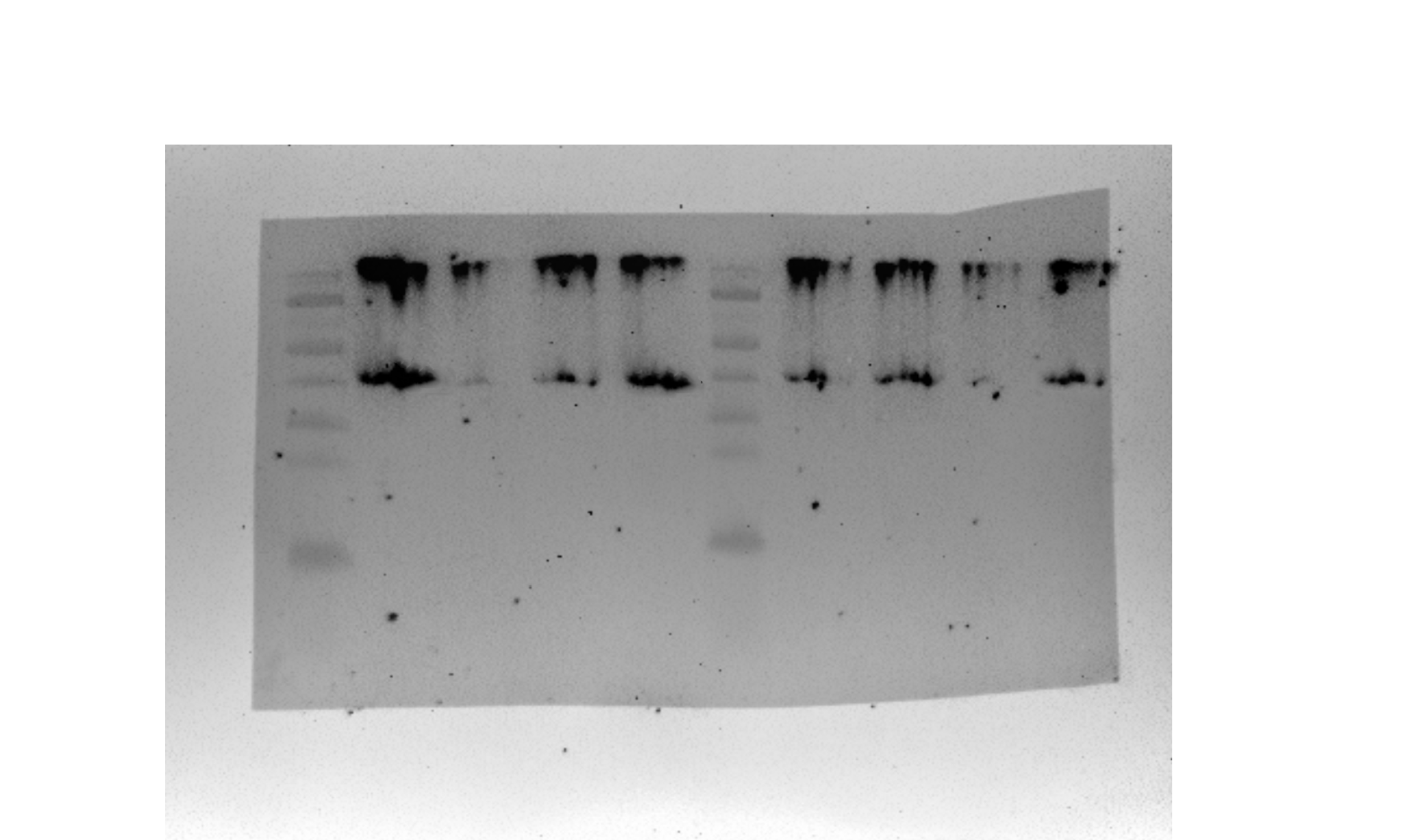

Supplement: Figure 7—source data 2. [file elife-97511-fig7-data2.zip › Figure7-source data2/WB of ArcA-2.tif]

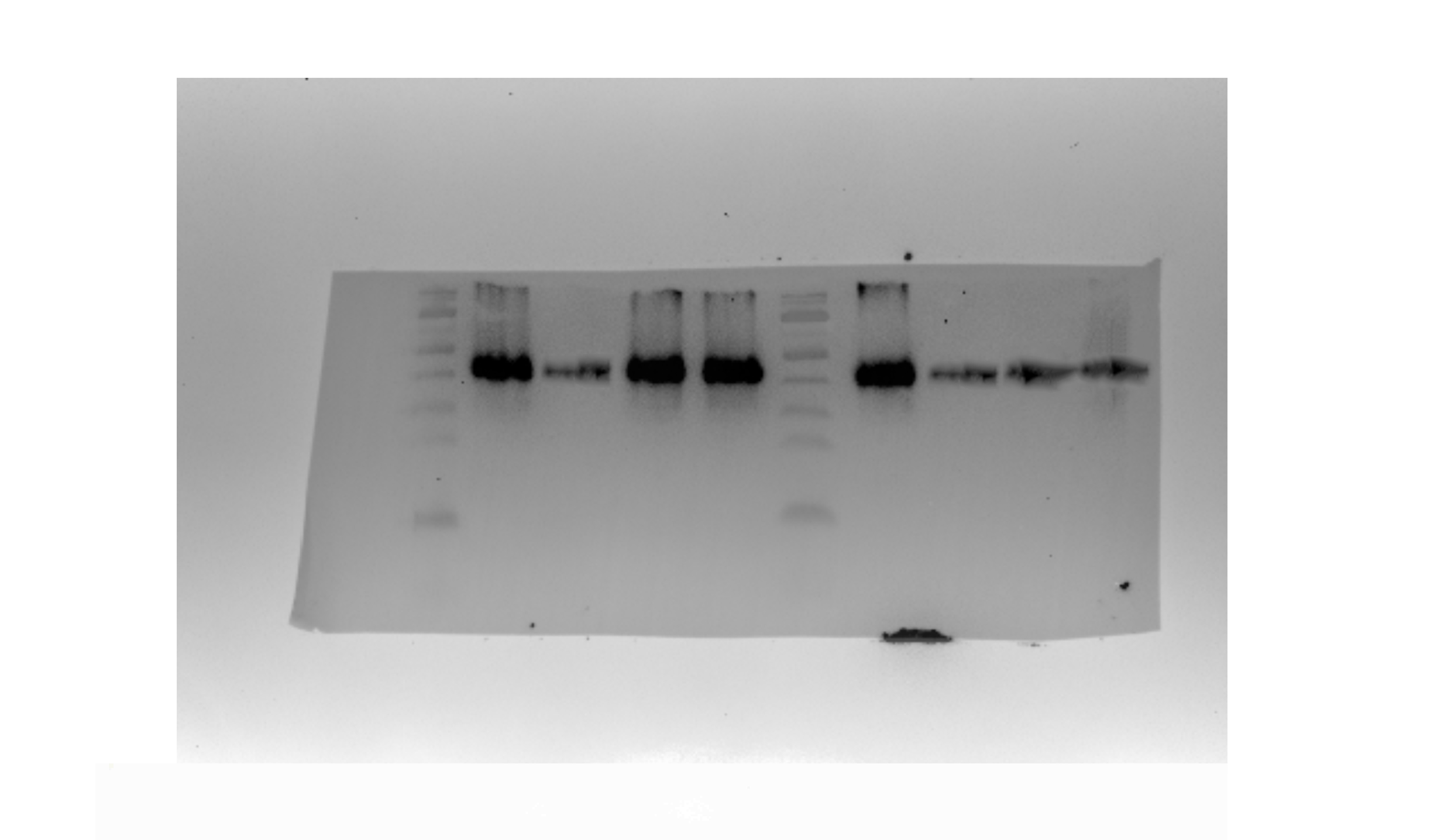

Supplement: Figure 7—source data 2. [file elife-97511-fig7-data2.zip › Figure7-source data2/WB of ENO.tif]

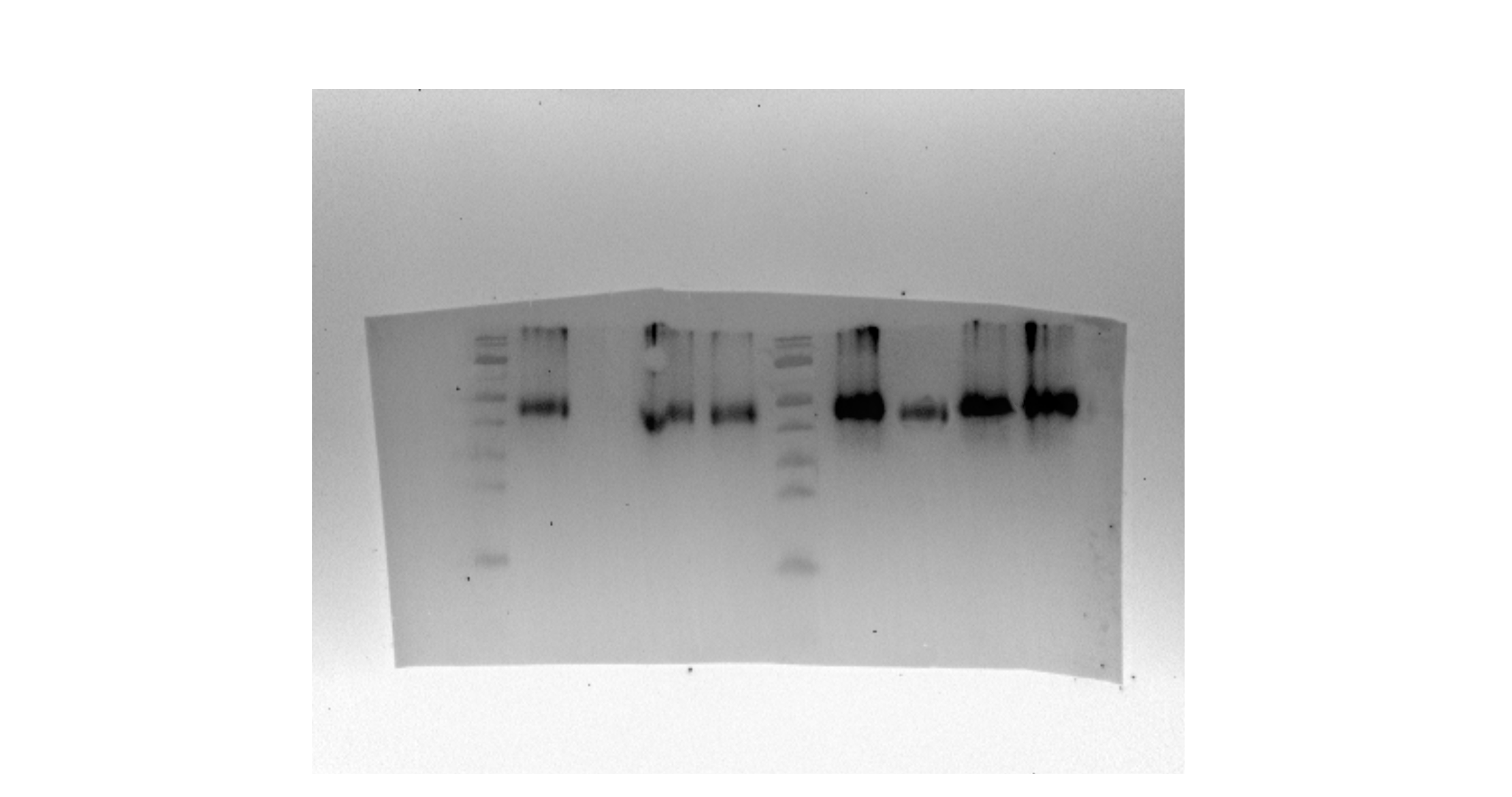

Supplement: Figure 7—source data 2. [file elife-97511-fig7-data2.zip › Figure7-source data2/WB of SUN.tif]

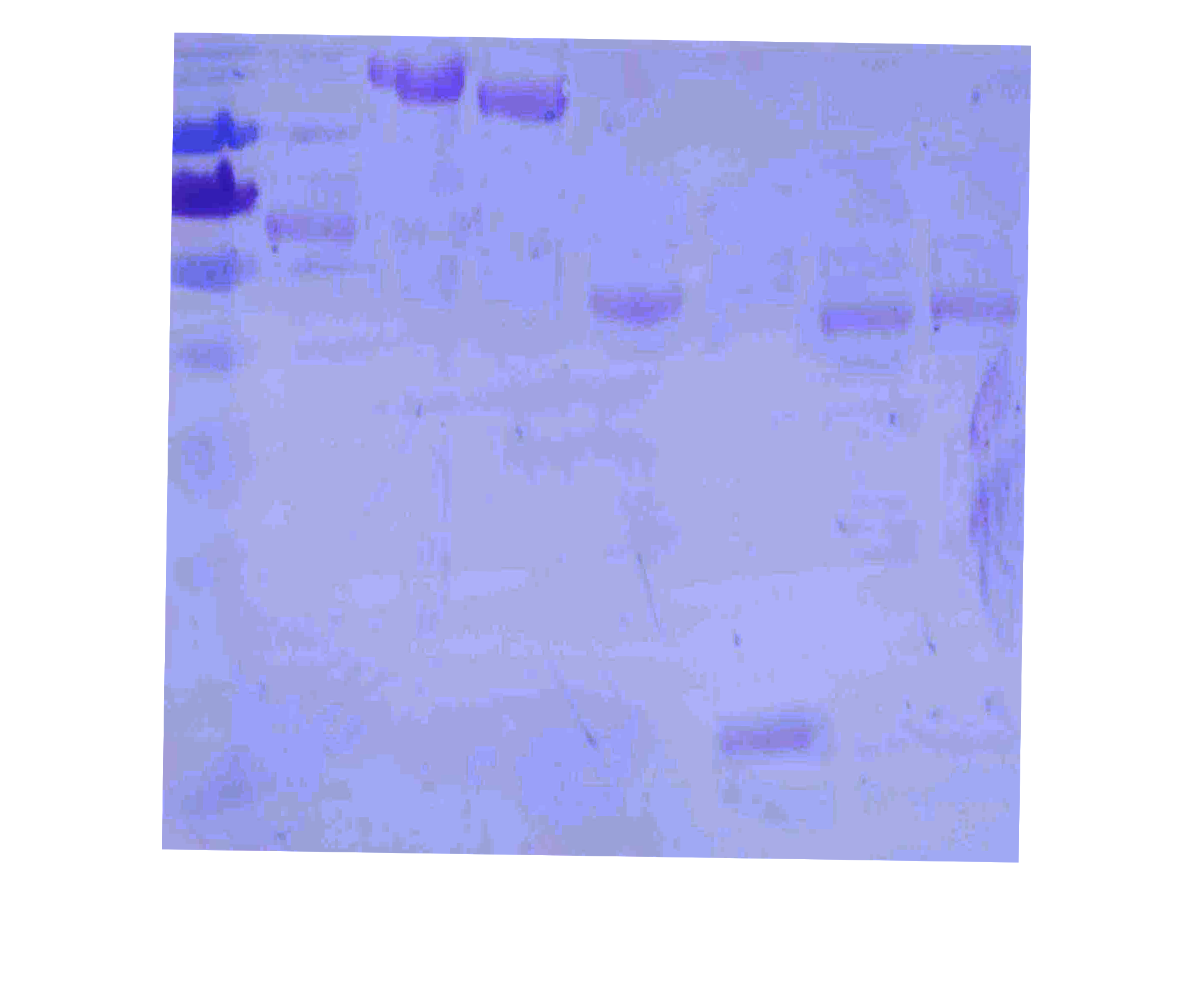

Supplement: Figure 7—figure supplement 1—source data 2. [file elife-97511-fig7-figsupp1-data2.zip › Figure 7-figure supplement 1-source data 2/loading control of Figure 7–figure supplement 1.tif]

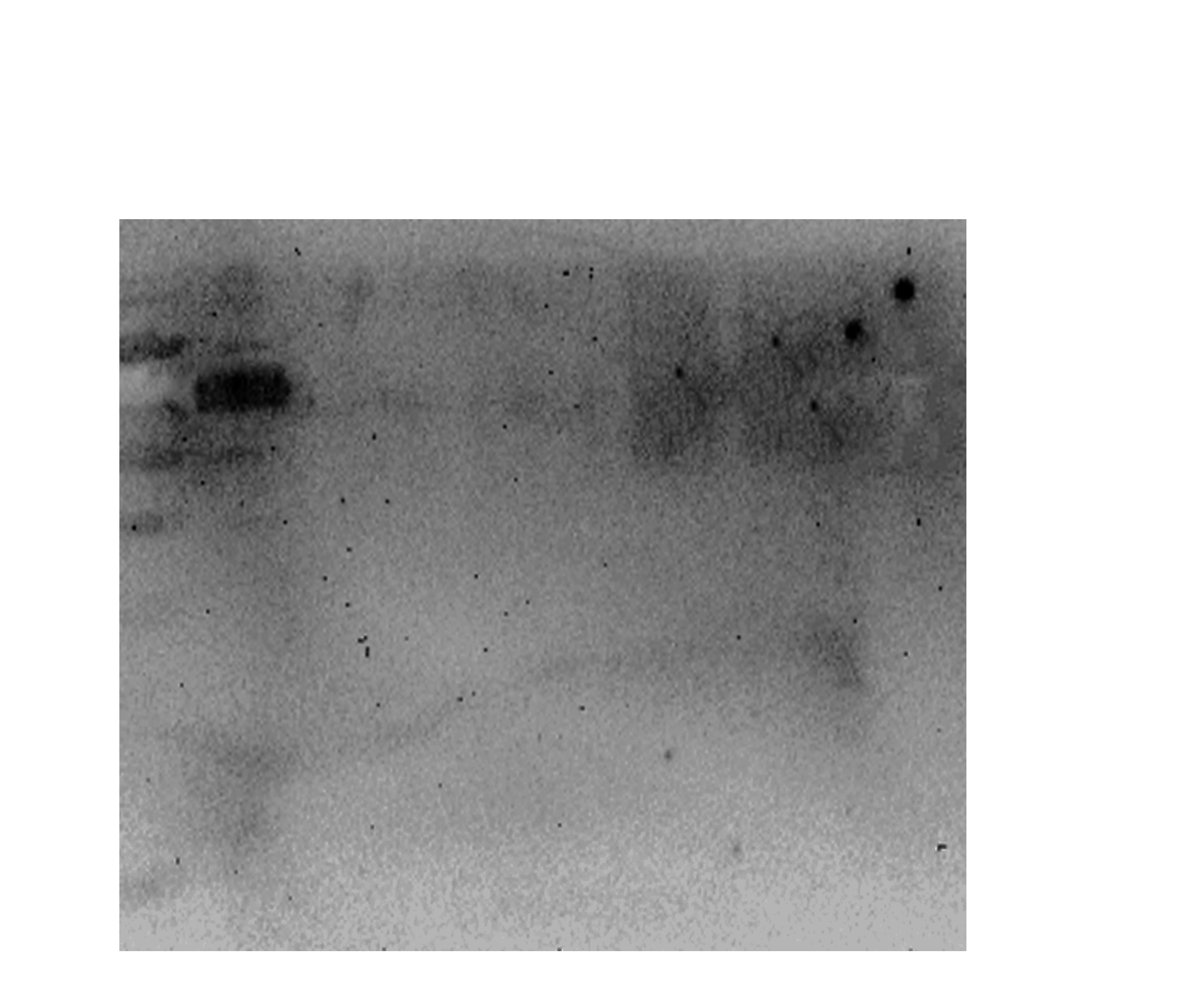

Supplement: Figure 7—figure supplement 1—source data 2. [file elife-97511-fig7-figsupp1-data2.zip › Figure 7-figure supplement 1-source data 2/WB of Figure 7–figure supplement 1.tif]

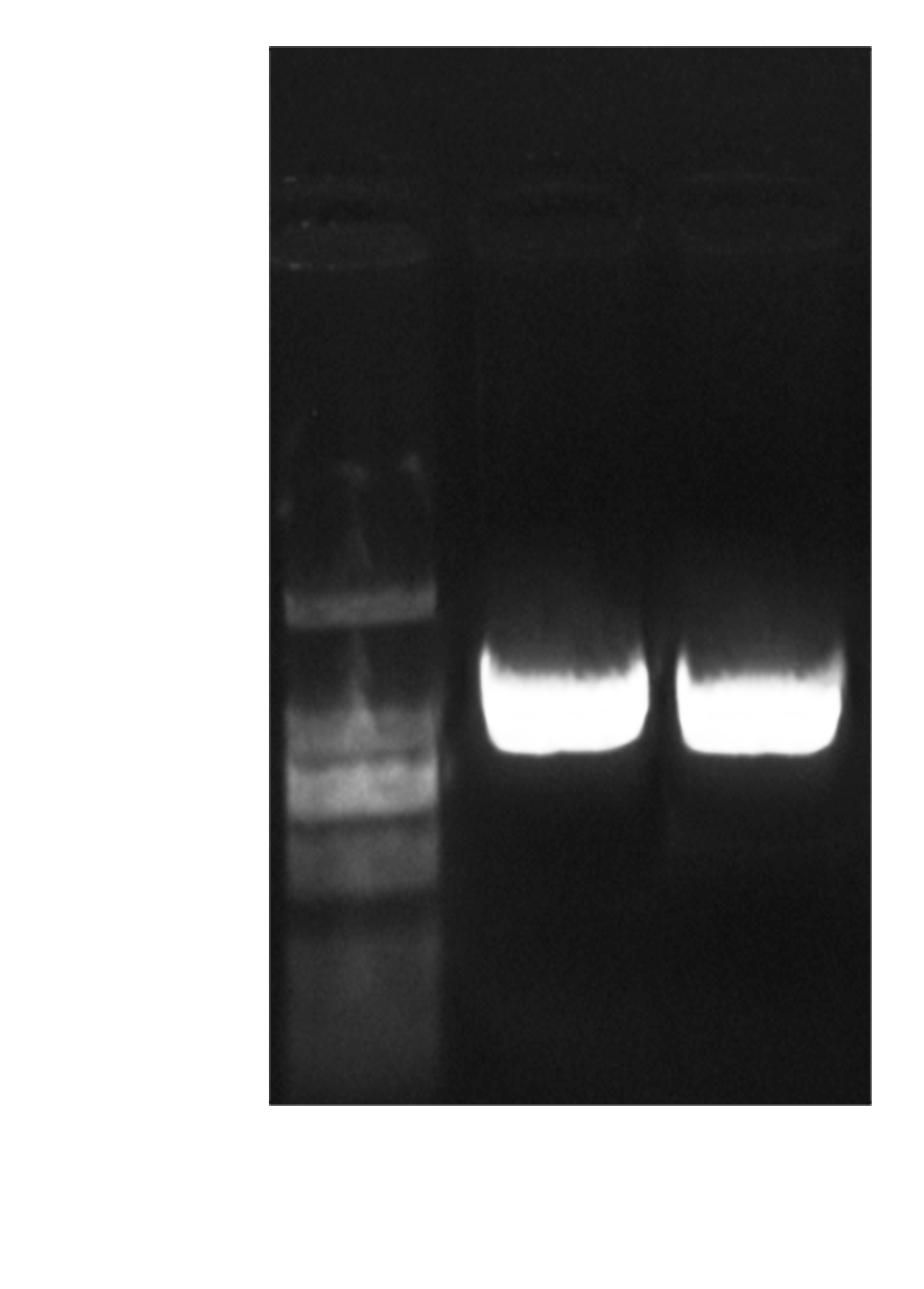

Supplement: Figure 7—figure supplement 2—source data 2. [file elife-97511-fig7-figsupp2-data2.zip › Figure 7-figure supplement 2-source data 2/PCR of ArcA-2 and ArcA-2-K26.tif]

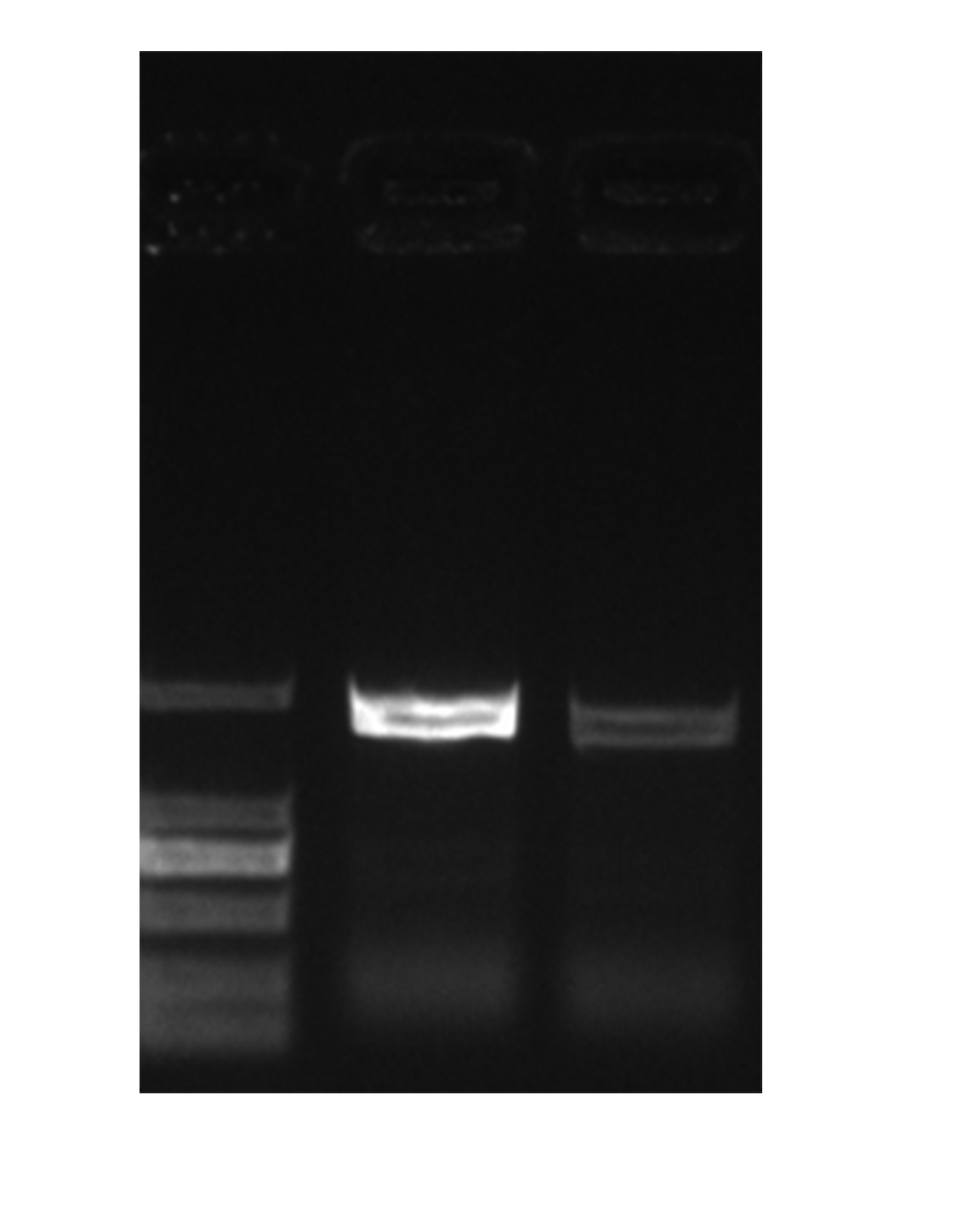

Supplement: Figure 7—figure supplement 2—source data 2. [file elife-97511-fig7-figsupp2-data2.zip › Figure 7-figure supplement 2-source data 2/PCR of ENO and ENO-K195.tif]

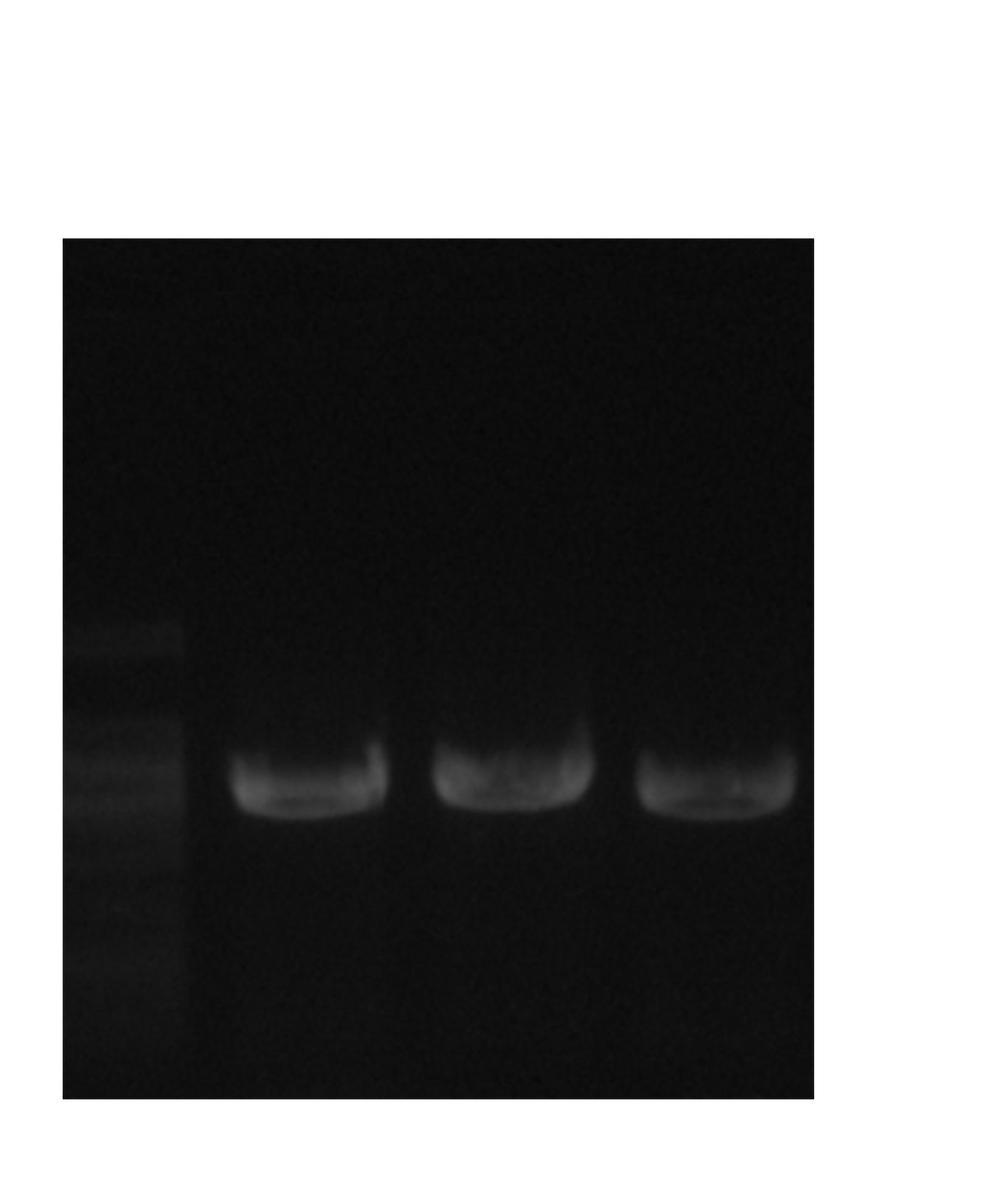

Supplement: Figure 7—figure supplement 2—source data 2. [file elife-97511-fig7-figsupp2-data2.zip › Figure 7-figure supplement 2-source data 2/PCR of SUN, SUN-K103, and SUN-K148.tif]

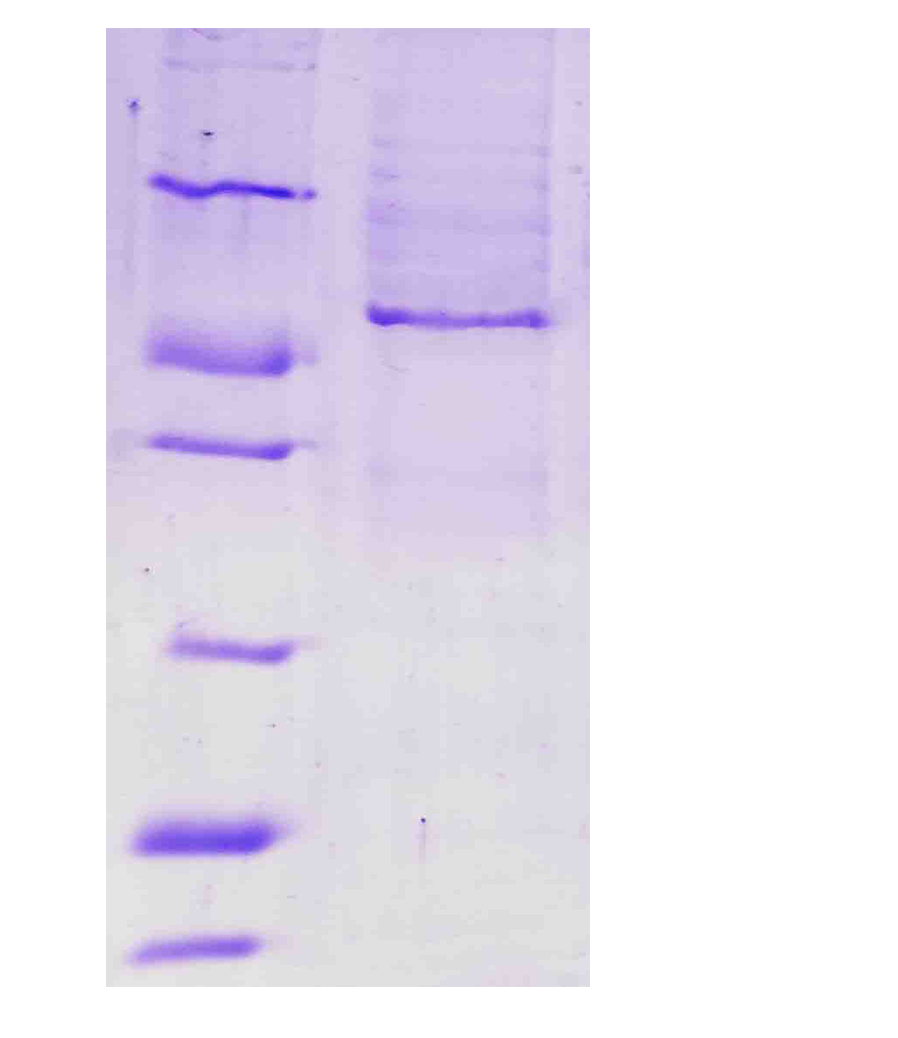

Supplement: Figure 7—figure supplement 2—source data 2. [file elife-97511-fig7-figsupp2-data2.zip › Figure 7-figure supplement 2-source data 2/SDS-PAGE of ENO-K195.tif]

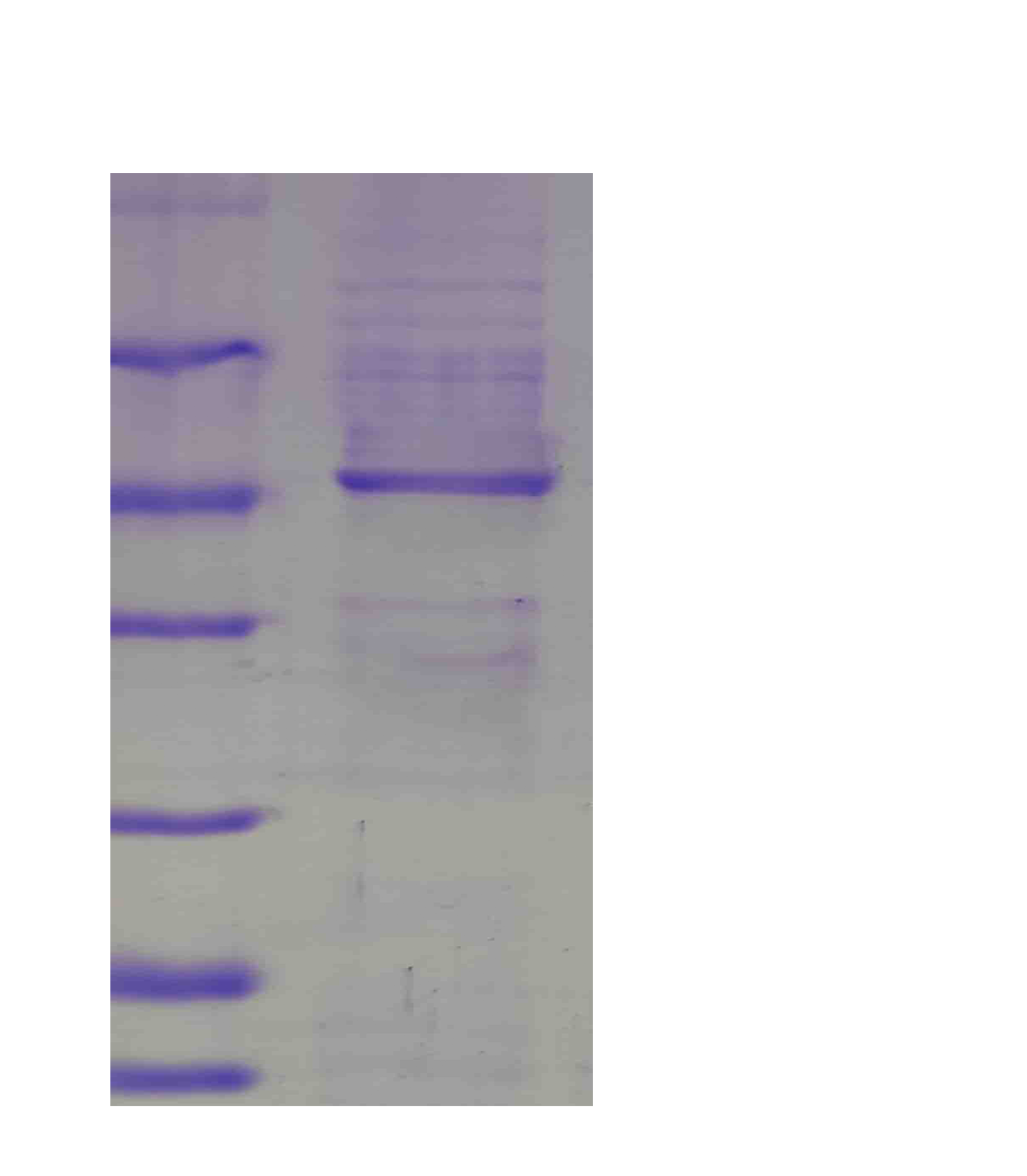

Supplement: Figure 7—figure supplement 2—source data 2. [file elife-97511-fig7-figsupp2-data2.zip › Figure 7-figure supplement 2-source data 2/SDS-PAGE of ArcA-2-K26.tif]

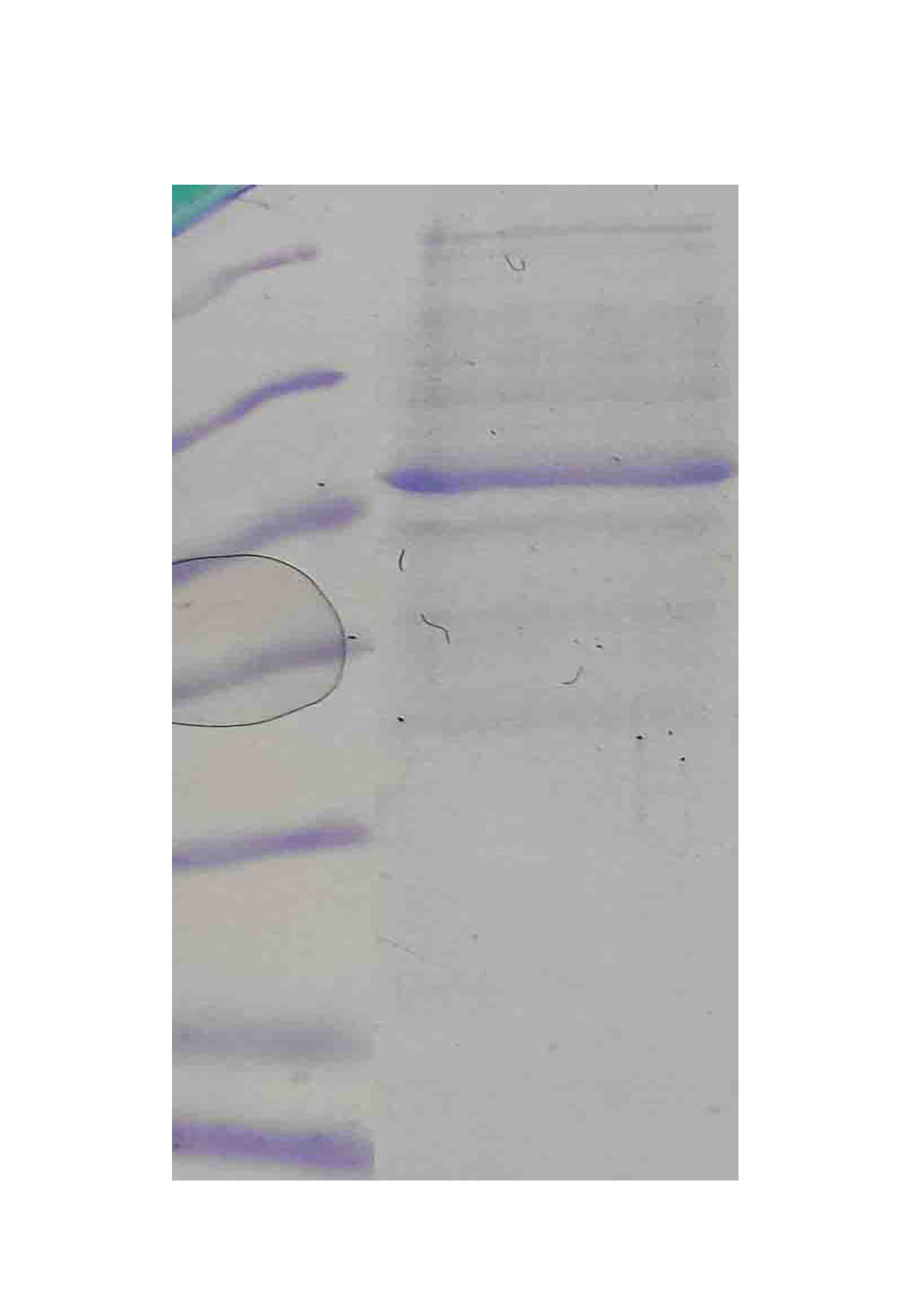

Supplement: Figure 7—figure supplement 2—source data 2. [file elife-97511-fig7-figsupp2-data2.zip › Figure 7-figure supplement 2-source data 2/SDS-PAGE of ArcA-2.tif]

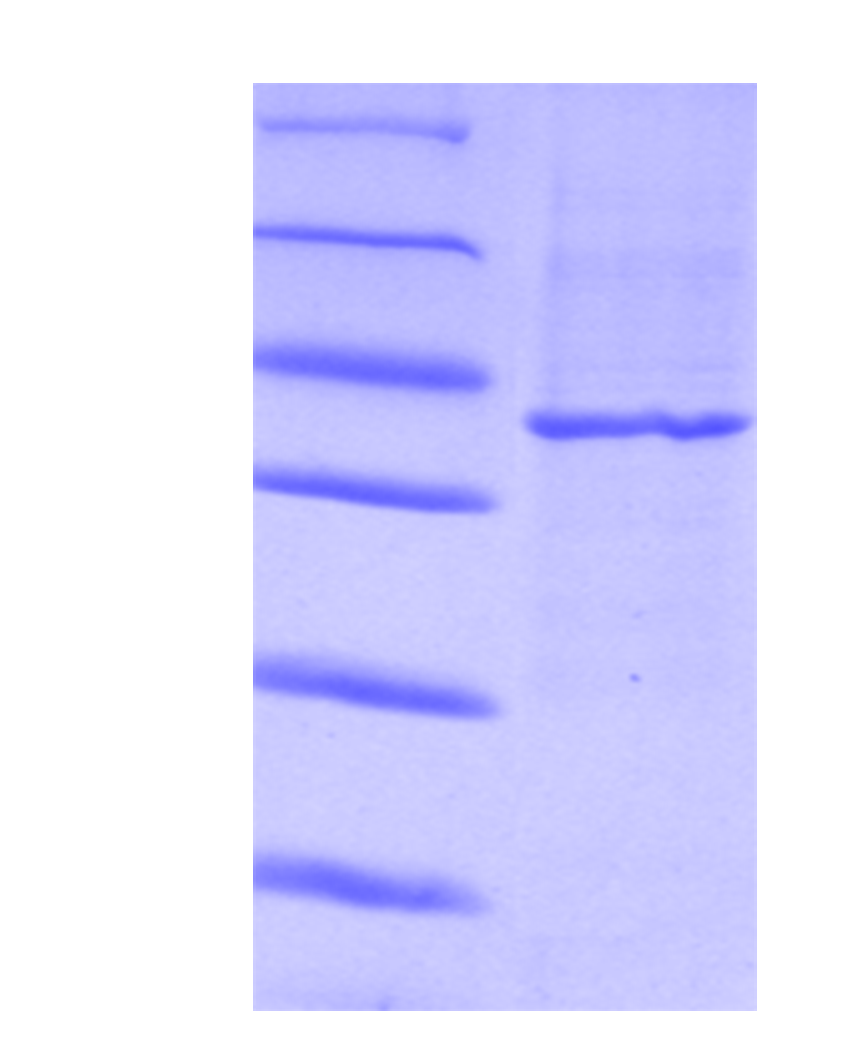

Supplement: Figure 7—figure supplement 2—source data 2. [file elife-97511-fig7-figsupp2-data2.zip › Figure 7-figure supplement 2-source data 2/SDS-PAGE of ENO.tif]

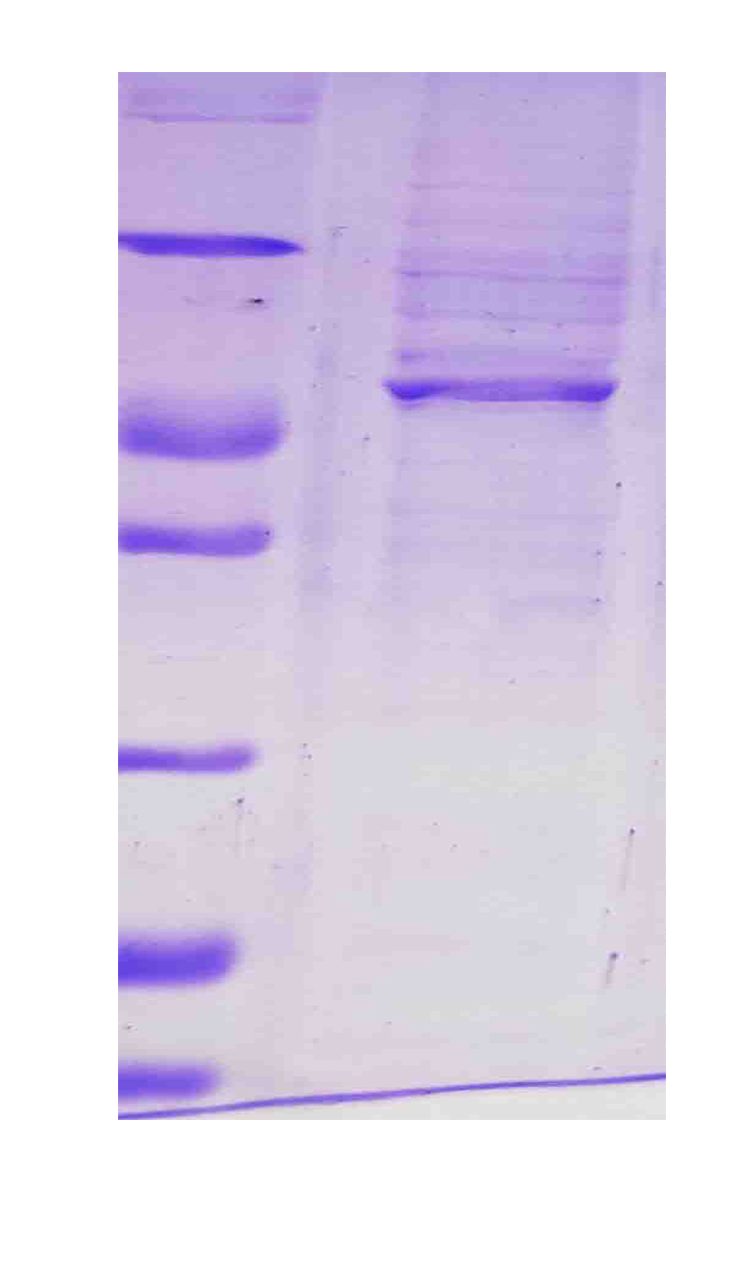

Supplement: Figure 7—figure supplement 2—source data 2. [file elife-97511-fig7-figsupp2-data2.zip › Figure 7-figure supplement 2-source data 2/SDS-PAGE of SUN-K103.tif]

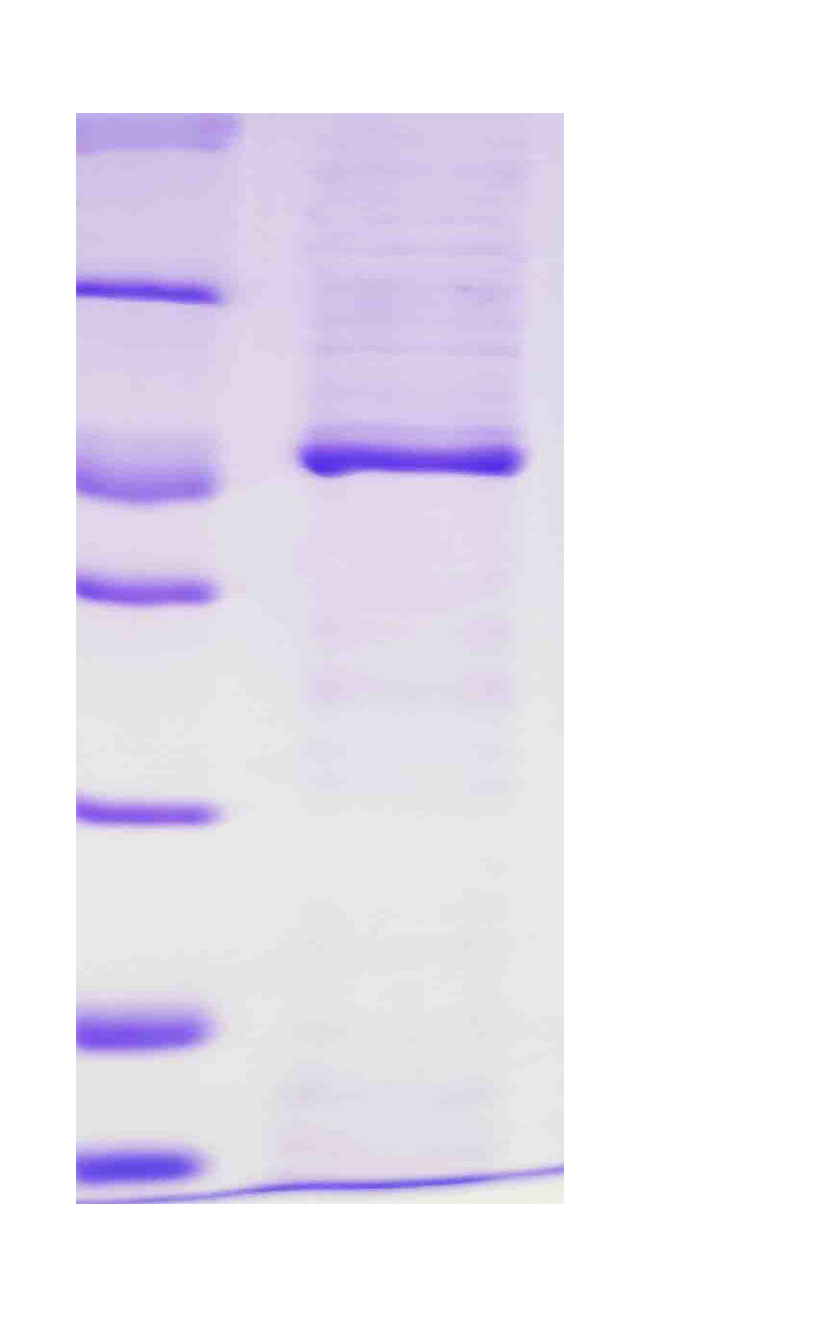

Supplement: Figure 7—figure supplement 2—source data 2. [file elife-97511-fig7-figsupp2-data2.zip › Figure 7-figure supplement 2-source data 2/SDS-PAGE of SUN-K148.tif]

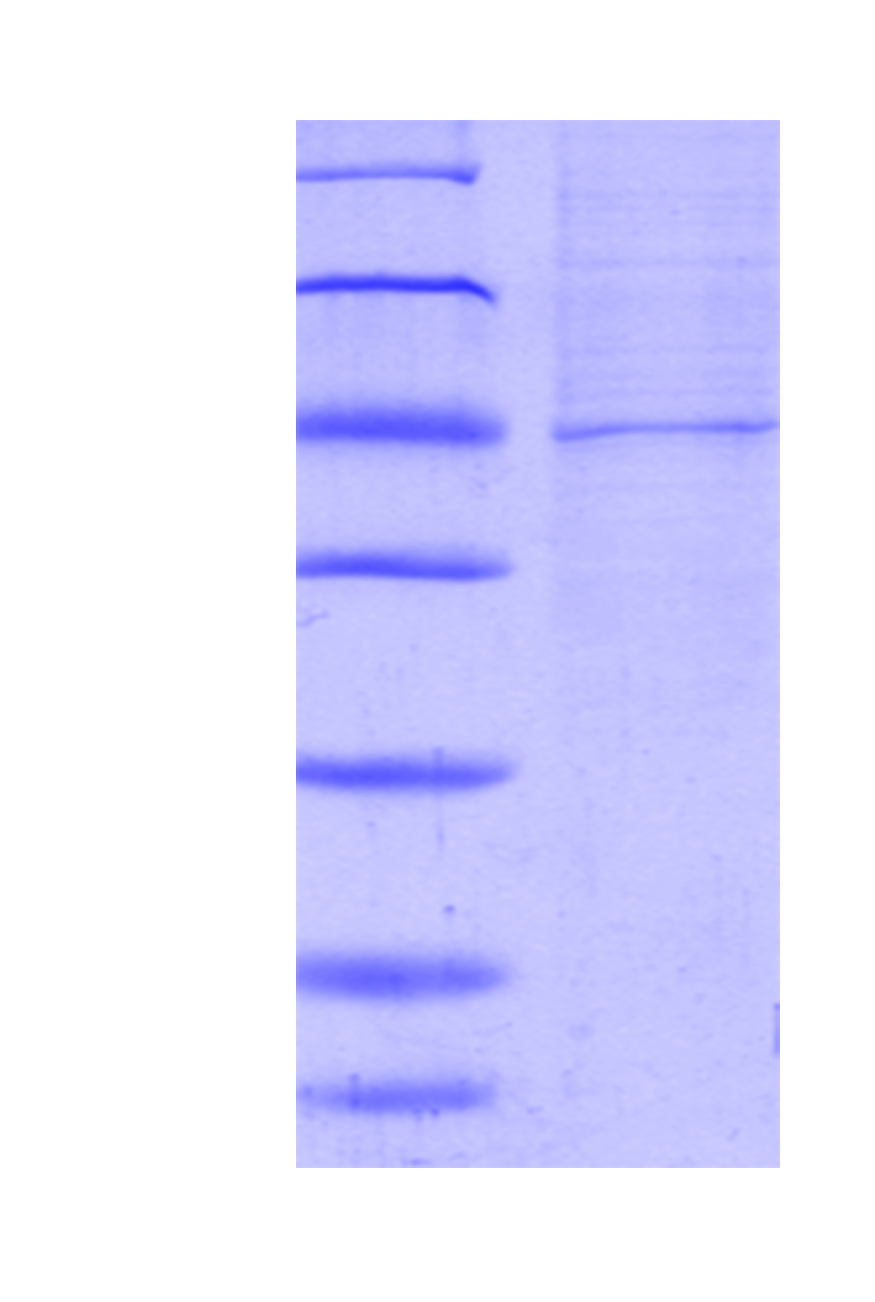

Supplement: Figure 7—figure supplement 2—source data 2. [file elife-97511-fig7-figsupp2-data2.zip › Figure 7-figure supplement 2-source data 2/SDS-PAGE of SUN.tif]

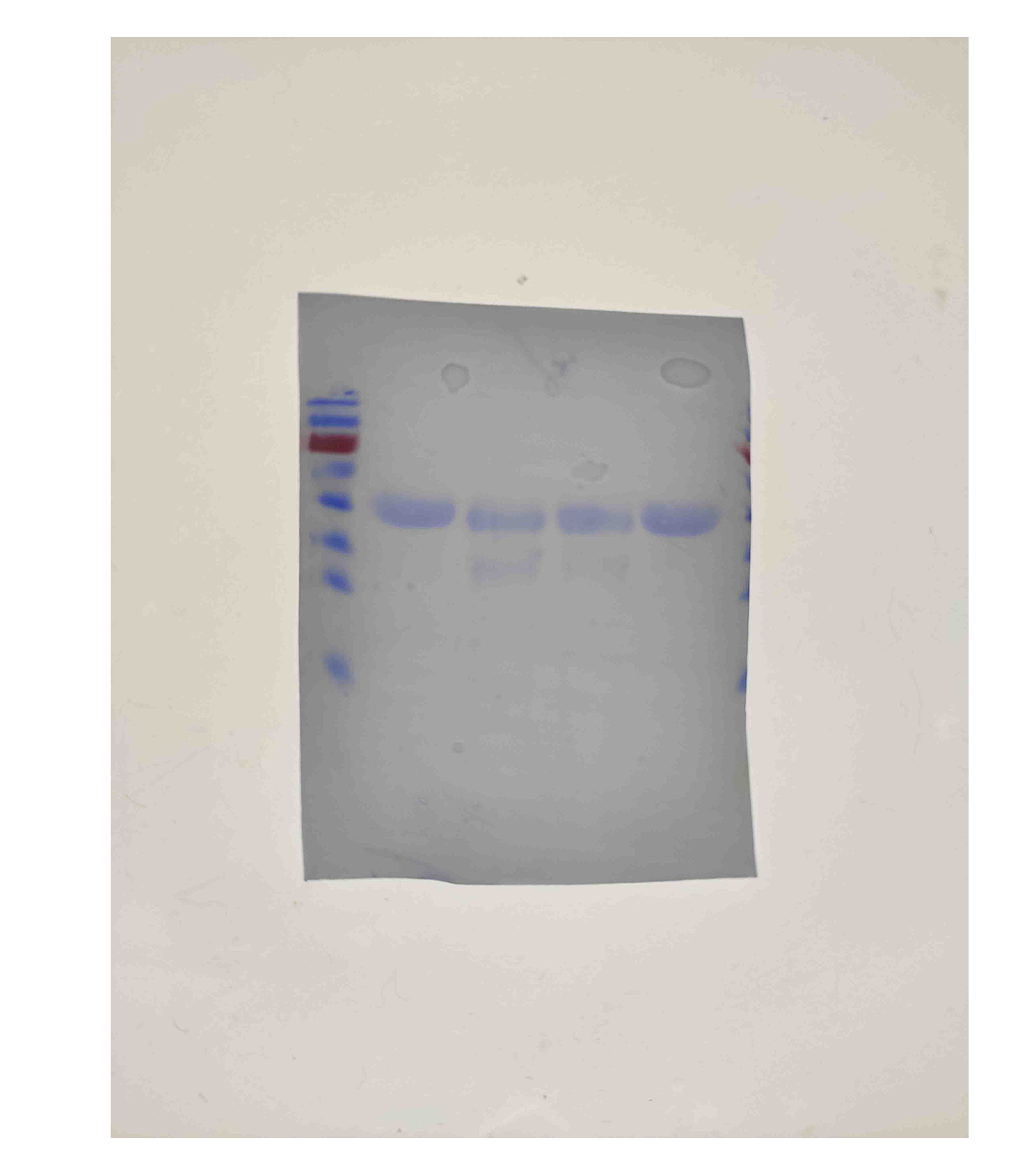

Supplement: Figure 8—source data 2. [file elife-97511-fig8-data2.zip › Figure8-source data2/loading control of Figure 8A.tif]

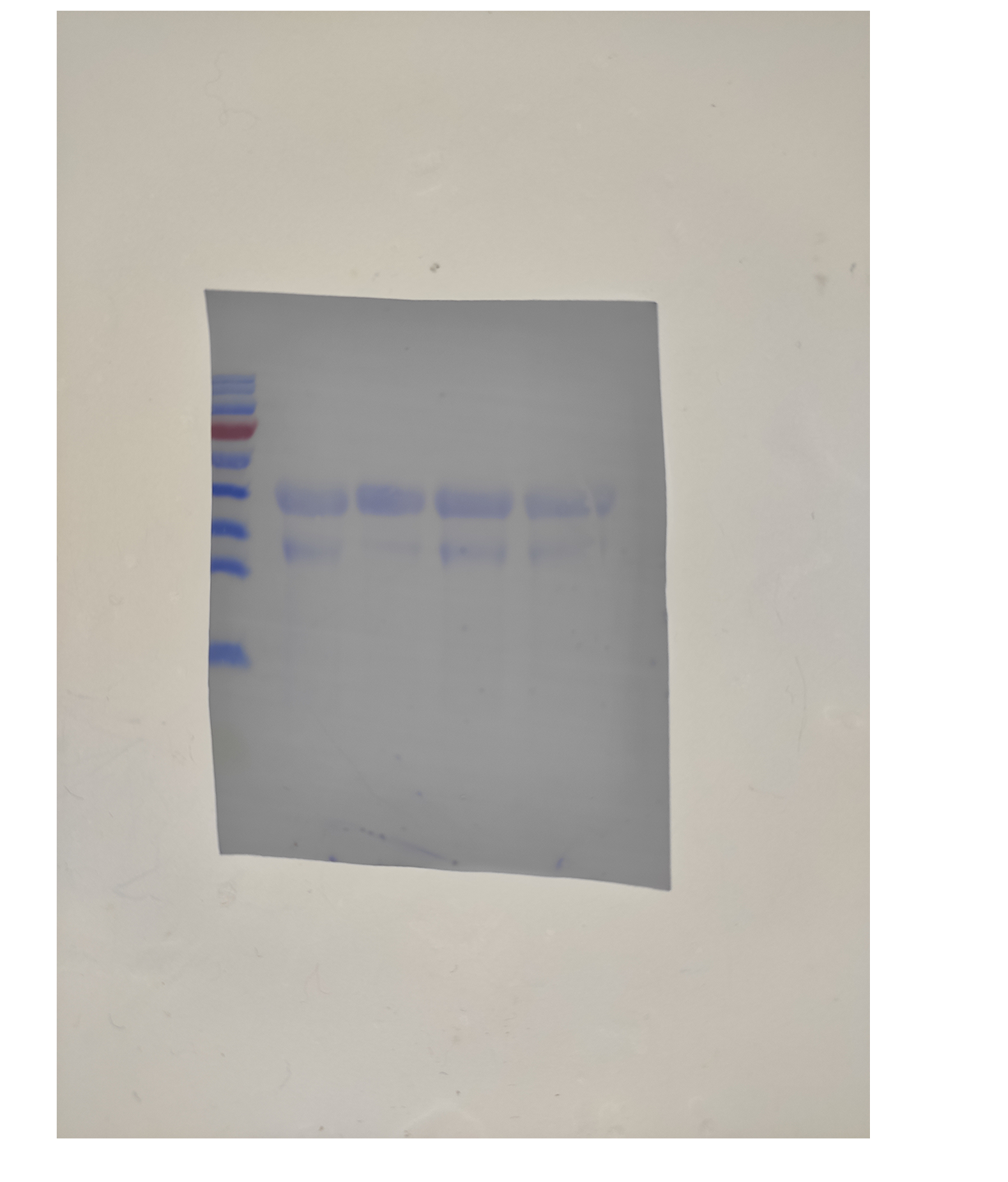

Supplement: Figure 8—source data 2. [file elife-97511-fig8-data2.zip › Figure8-source data2/loading control of Figure 8B.tif]

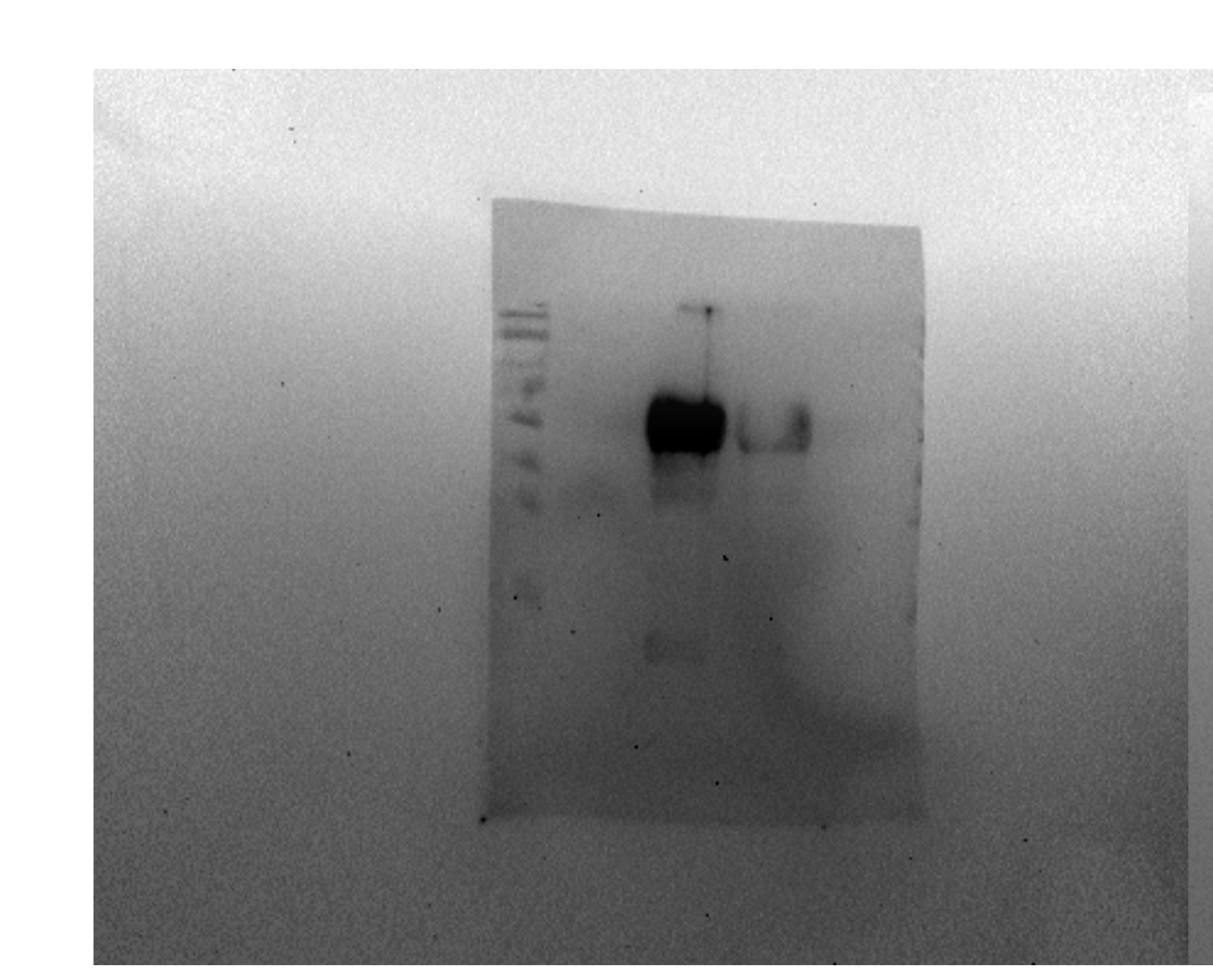

Supplement: Figure 8—source data 2. [file elife-97511-fig8-data2.zip › Figure8-source data2/WB of Figure 8A.tif]

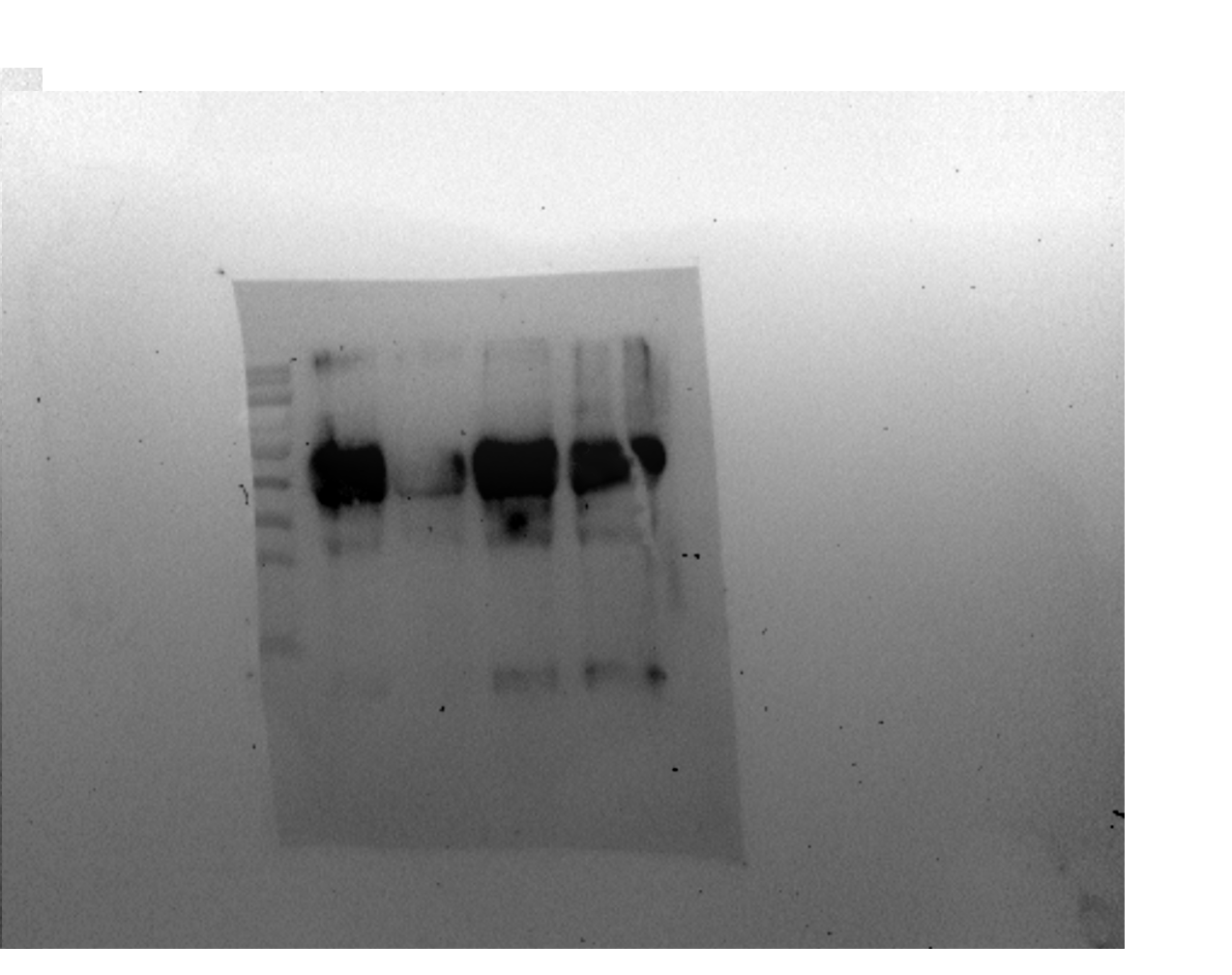

Supplement: Figure 8—source data 2. [file elife-97511-fig8-data2.zip › Figure8-source data2/WB of Figure 8B.tif]
