# Supplementary material for: Aeromonas hydrophila CobQ is a new type of NAD+- and Zn2+-independent protein lysine deacetylase
Source: eLife. 2025 Feb 25;13:RP97511. doi: 10.7554/eLife.97511 (PMC11856932; doi:10.7554/eLife.97511)
Supplement: Figure 2—source data 1. [file elife-97511-fig2-data1.zip › Figure 2—source data 1.pdf]

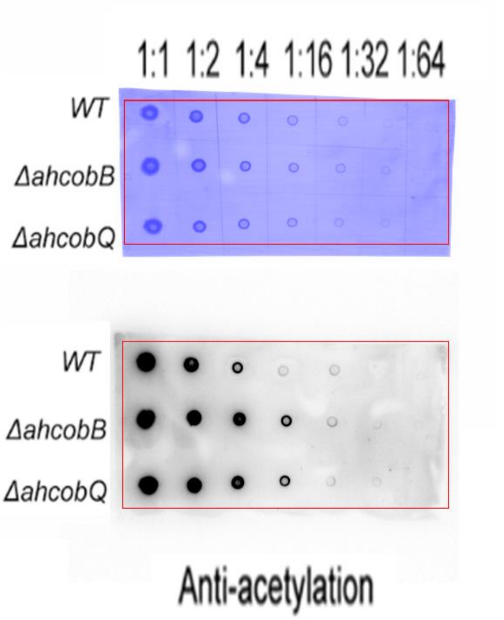
1:1 1:2 1:4 1:16 1:32 1:64
WT
ΔahcobB
ΔahcobQ
WT
ΔahcobB
ΔahcobQ
Anti-acetylation

A

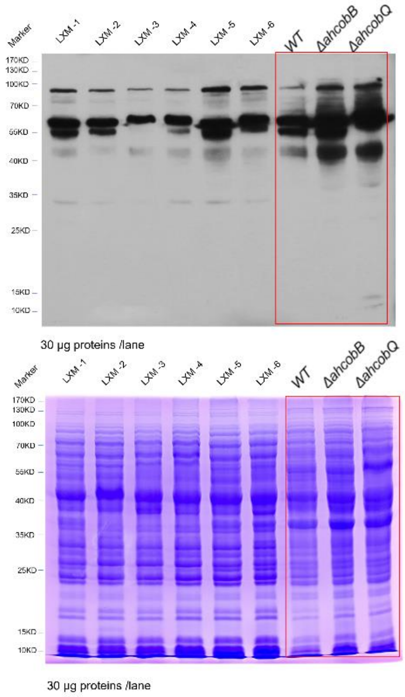
Marker
LXM-1
LXM-2
LXM-3
LXM-4
LXM-5
LXM-6
WT
ΔahcobB
ΔahcobQ
170KD
130KD
100KD
70KD
55KD
40KD
35KD
25KD
15KD
10KD
30 μg proteins /lane
Marker
LXM-1
LXM-2
LXM-3
LXM-4
LXM-5
LXM-6
WT
ΔahcobB
ΔahcobQ
170KD
130KD
100KD
70KD
55KD
40KD
35KD
25KD
15KD
10KD
30 μg proteins /lane

B

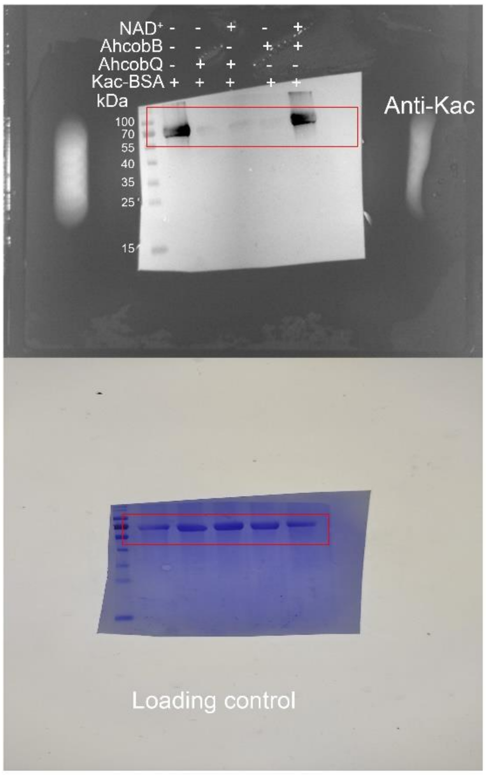
NAD+ - - + - +
AhcobB - - - + +
AhcobQ - + + - -
Kac-BSA + + + + +
kDa
100
70
55
40
35
25
15
Anti-Kac
Loading control

C

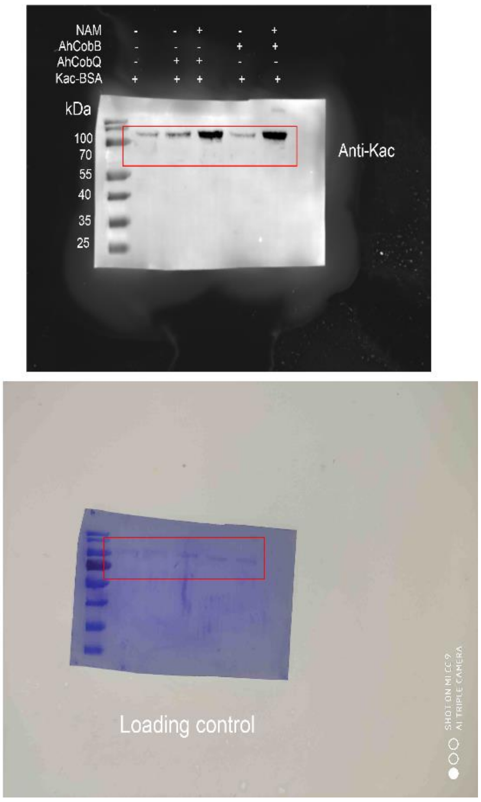
NAM - - + - +
AhCobB - - - + +
AhCobQ - + + - -
Kac-BSA + + + + +
kDa
100
70
55
40
35
25
Anti-Kac
Loading control

D

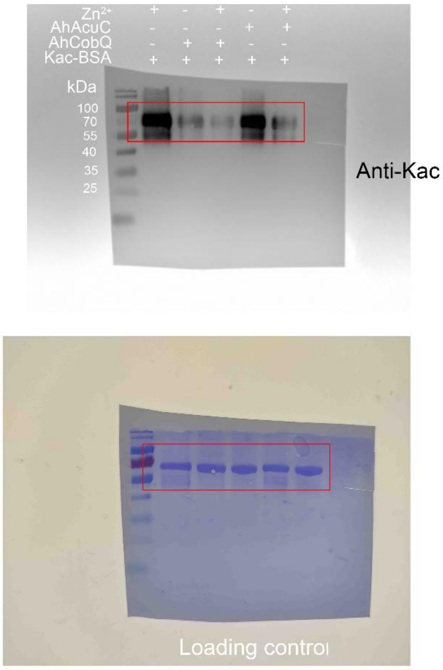

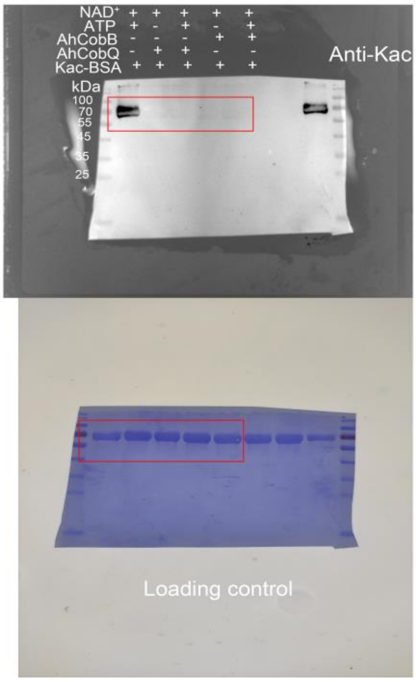

E F

Figure 2—source data 1. Original files for western blot analysis displayed in Figure 2C-H. (A) and (B) Dot blot and western blot verified the whole cell protein Kac level among WT, Δ*ahcobB*, and Δ*ahcobQ* strains. (C–F) Effect of NAD^+, NAM, Zn^{2+}, and ATP on KDAC enzymatic activity of AhCobQ.
