# Supplementary material for: Aeromonas hydrophila CobQ is a new type of NAD+- and Zn2+-independent protein lysine deacetylase
Source: eLife. 2025 Feb 25;13:RP97511. doi: 10.7554/eLife.97511 (PMC11856932; doi:10.7554/eLife.97511)
Supplement: Figure 2—figure supplement 2—source data 1. [file elife-97511-fig2-figsupp2-data1.zip › Figure 2–figure supplement 2—source data 1.pdf]

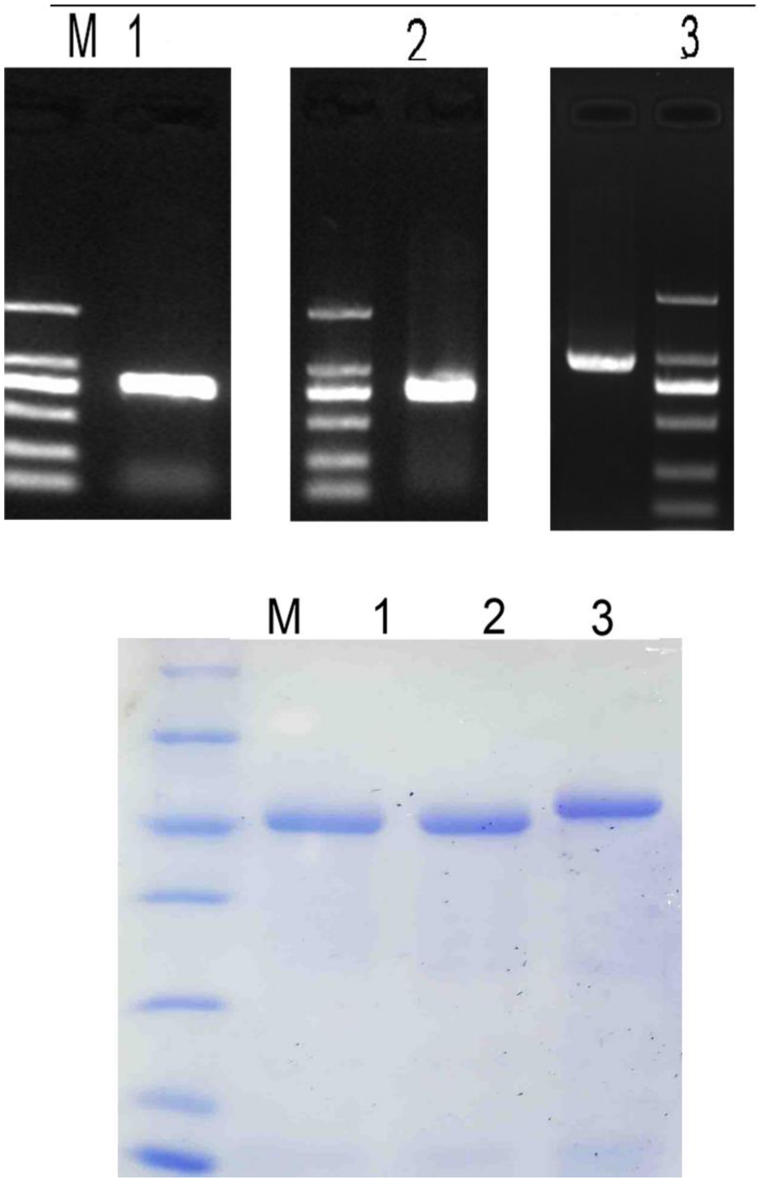

Figure 2–figure supplement 2—source data 1. Original files for western blot analysis displayed in Figure 2– figure supplement 2. Characteristics of overexpressed and purified re combinant proteins AhCobQ, AhCobB, and AhAcuC. PCR amplification results of ahcobQ, a hcobB, and ahacuC gene, respectively; SDS-PAGE gels of purified recombinant proteins Ah CobQ, AhCoB, and AhAcuC, respectively. Lane M represents the DNA or protein marker. M: Marker; Lane 1: AhCobQ; Lane 2: AhCobB; Lane 3: AhAcuC.
