# Supplementary material for: Aeromonas hydrophila CobQ is a new type of NAD+- and Zn2+-independent protein lysine deacetylase
Source: eLife. 2025 Feb 25;13:RP97511. doi: 10.7554/eLife.97511 (PMC11856932; doi:10.7554/eLife.97511)
Supplement: Figure 2—figure supplement 3—source data 1. [file elife-97511-fig2-figsupp3-data1.zip › Figure 2–figure supplement 3—source data 1.pdf]

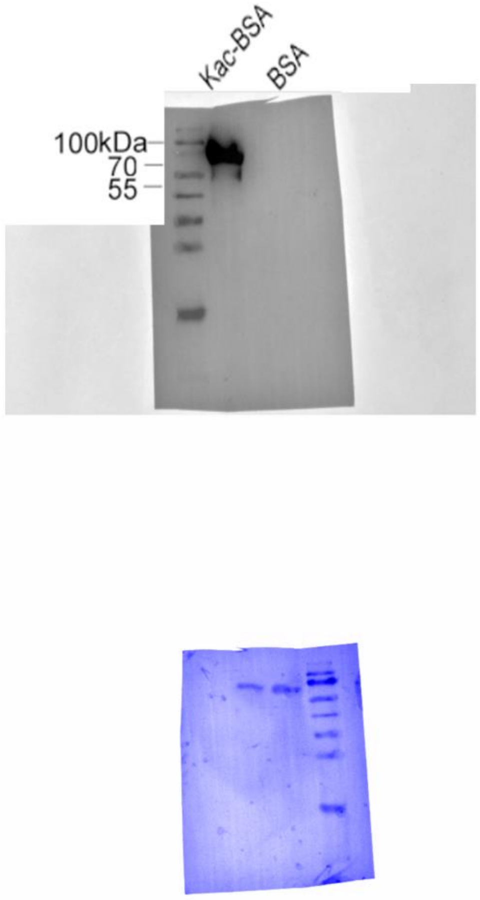

Figure 2–figure supplement 3—source data 1. Original files for western blot analysis displayed in Figure 2–figure supplement 3. In vitro acetylated BSA (Kac-BSA) was verified by western blot.
