# Supplementary material for: Aeromonas hydrophila CobQ is a new type of NAD+- and Zn2+-independent protein lysine deacetylase
Source: eLife. 2025 Feb 25;13:RP97511. doi: 10.7554/eLife.97511 (PMC11856932; doi:10.7554/eLife.97511)
Supplement: Figure 2—figure supplement 4—source data 1. [file elife-97511-fig2-figsupp4-data1.zip › Figure 2–figure supplement 4—source data 1.pdf]

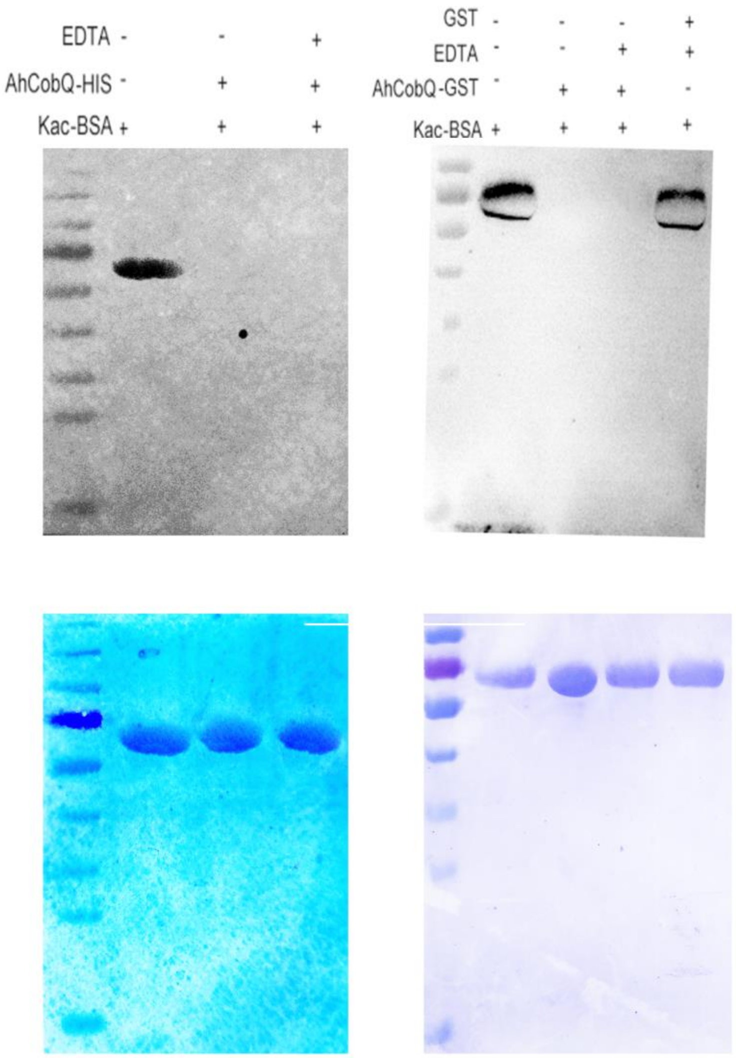

Figure 2–figure supplement 4—source data 1. Original files for western blot analysis displayed in Figure 2–figure supplement 4. In vitro deacetylase activity assay of different recombinant AhCobQ proteins in the presence and absence of EDTA utilizing Kac-BSA as substrate. Purified His-tag and GST-fused recombinant AhCobQ incubated with the substrate Kac-BSA, both in the absence and presence of 150 μM EDTA treatment, respectively.
