# Supplementary material for: Aeromonas hydrophila CobQ is a new type of NAD+- and Zn2+-independent protein lysine deacetylase
Source: eLife. 2025 Feb 25;13:RP97511. doi: 10.7554/eLife.97511 (PMC11856932; doi:10.7554/eLife.97511)
Supplement: Figure 2—figure supplement 5—source data 1. [file elife-97511-fig2-figsupp5-data1.zip › Figure 2–figure supplement 5—source data 1.pdf]

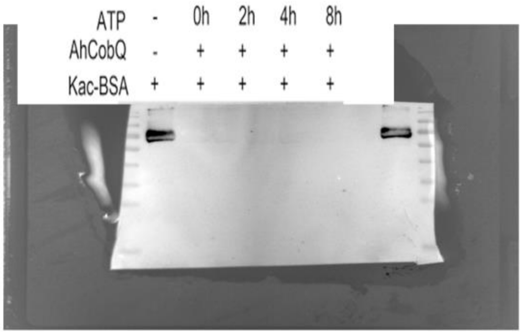

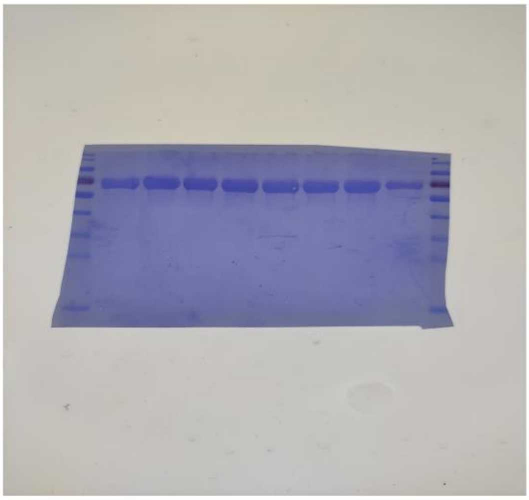

Figure 2–figure supplement 5—source data 1. Original files for western blot analysis displayed in Figure 2–figure supplement 5. The lysine deacetylase activity of AhCobQ didn't be affected by 0.5mM ATP at different incubation times.
