# Supplementary material for: Aeromonas hydrophila CobQ is a new type of NAD+- and Zn2+-independent protein lysine deacetylase
Source: eLife. 2025 Feb 25;13:RP97511. doi: 10.7554/eLife.97511 (PMC11856932; doi:10.7554/eLife.97511)
Supplement: Figure 4—source data 1. [file elife-97511-fig4-data1.zip › Figure 4—source data 1.pdf]

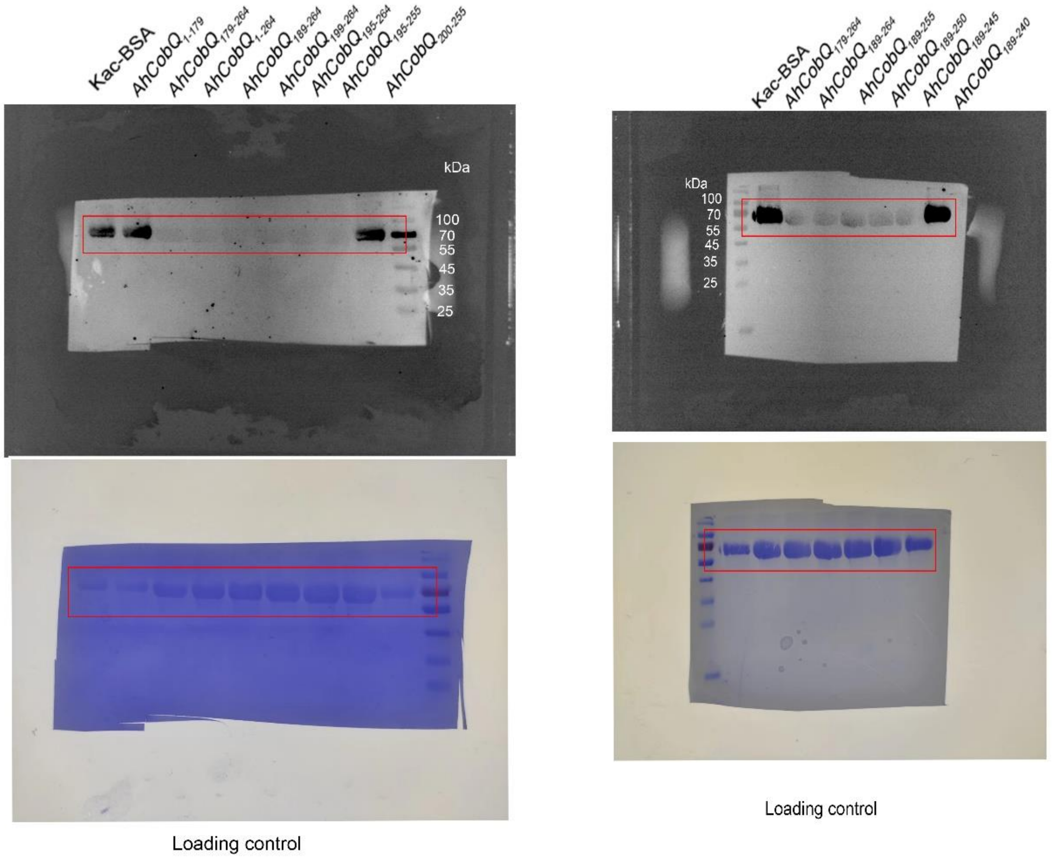

A B

Figure 4—source data 1. Original files for western blot analysis displayed in Figure 4A, C. Western blot analysis of the KDAC activity of (A) $AhCobQ_{1–179}$, $AhCobQ_{179–264}$, $AhCobQ_{1–264}$, $AhCobQ_{189–264}$, $AhCobQ_{199–264}$, $AhCobQ_{195–264}$, $AhCobQ_{195–255}$, and $AhCobQ_{200–255}$; (B) $AhCobQ_{179–264}$, $AhCobQ_{189–264}$, $AhCobQ_{189–255}$, $AhCobQ_{189–250}$, $AhCobQ_{189–245}$, and $AhCobQ_{189–240}$ truncated proteins treated with Kac-BSA. The first lane represents Kac-BSA without AhCobQ truncated proteins. The upper part of the figure shows the WB results of the Kac level of truncated proteins with Kac-BSA, and the lower part shows the PVDF membrane R350 staining for the loading amount control.
