# Supplementary material for: Aeromonas hydrophila CobQ is a new type of NAD+- and Zn2+-independent protein lysine deacetylase
Source: eLife. 2025 Feb 25;13:RP97511. doi: 10.7554/eLife.97511 (PMC11856932; doi:10.7554/eLife.97511)
Supplement: Figure 4—figure supplement 1—source data 1. [file elife-97511-fig4-figsupp1-data1.zip › Figure 4–figure supplement 1—source data 1.pdf]

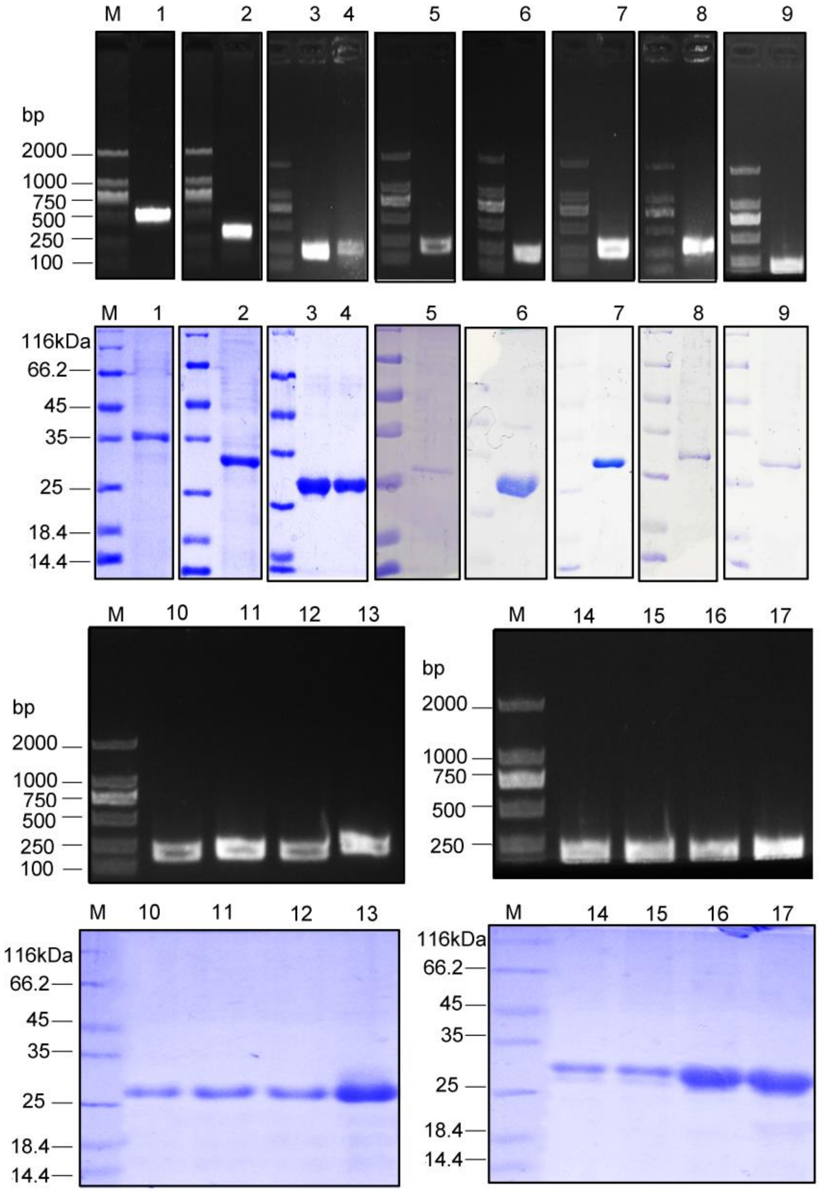

Figure 4–figure supplement 1—source data 1. Original files for PCR and SDS-PAGE analysis displayed in Figure 4–figure supplement 1. Characteristics of overexpressed and purified recombinant AhCobQ truncated proteins. The PCR amplification and SDS-PAGE results of purified recombinant truncated proteins (GST-fusion) 1: AhCobQ$_{1-179}$; 2: AhCobQ$_{179-265}$; 3: AhCobQ$_{189-265}$; 4: AhCobQ$_{189-255}$; 5: AhCobQ$_{189-250}$; 6: AhCobQ$_{189-245}$; 7: AhCobQ$_{189-240}$; 8: AhCobQ$_{195-255}$; 9: AhCobQ$_{200-255}$; 10: AhCobQ$_{179-225}$; 11: AhCobQ$_{179-235}$; 12: AhCobQ$_{179-245}$; 13: AhCobQ$_{179-255}$; 14: AhCobQ$_{189-265}$; 15: AhCobQ$_{199-265}$; 16: AhCobQ$_{209-265}$; 17: AhCobQ$_{219-265}$.
