# Supplementary material for: Aeromonas hydrophila CobQ is a new type of NAD+- and Zn2+-independent protein lysine deacetylase
Source: eLife. 2025 Feb 25;13:RP97511. doi: 10.7554/eLife.97511 (PMC11856932; doi:10.7554/eLife.97511)
Supplement: Figure 6—figure supplement 1—source data 1. [file elife-97511-fig6-figsupp1-data1.zip › Figure 6–figure supplement 1—source data 1.pdf]

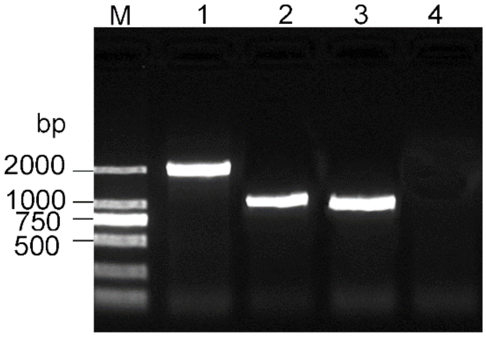

Figure 6–figure supplement 1—source data 1. Original files for PCR analysis displayed in Figure 6–figure supplement 1. Construction of *A. hydrophila ahacuC* knockout mutant strain M: Marker; Lanes 1 and 2: PCR products of WT and ∆*ahacuC* using the P7/P8 primer pairs; Lanes 3 and 4: PCR products of WT and ∆*ahacuC* using the P5/P6 primer pairs.
