# Supplementary material for: Aeromonas hydrophila CobQ is a new type of NAD+- and Zn2+-independent protein lysine deacetylase
Source: eLife. 2025 Feb 25;13:RP97511. doi: 10.7554/eLife.97511 (PMC11856932; doi:10.7554/eLife.97511)
Supplement: Figure 7—source data 1. [file elife-97511-fig7-data1.zip › Figure 7—source data 1.pdf]

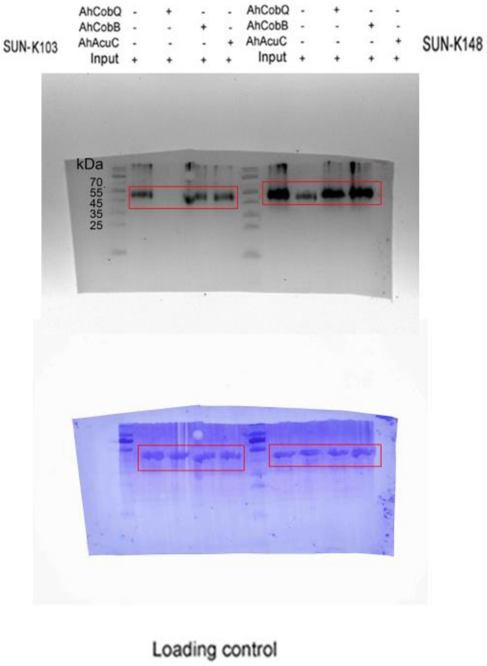


A

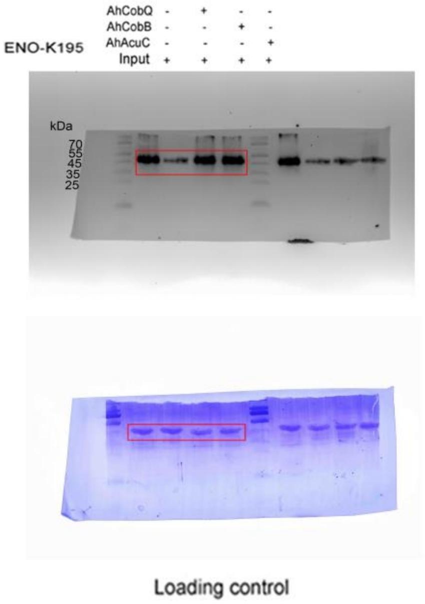


B

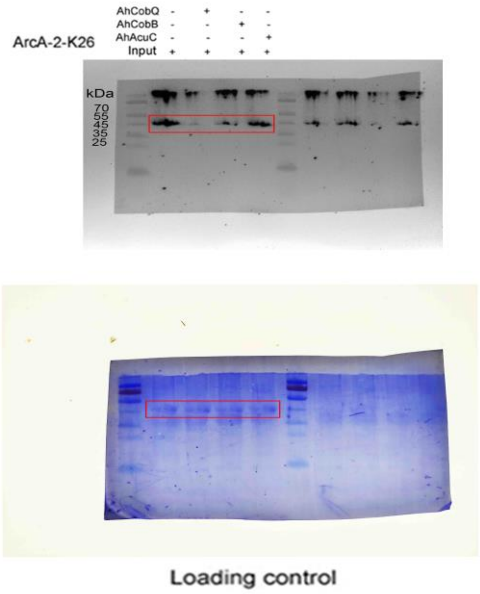


C

**Figure 7—source data 1.** Original files for western blot analysis displayed in Figure 7. Western blot validation of the site-specific Kac protein substrates regulated by the three KDACs.
