# Supplementary material for: Aeromonas hydrophila CobQ is a new type of NAD+- and Zn2+-independent protein lysine deacetylase
Source: eLife. 2025 Feb 25;13:RP97511. doi: 10.7554/eLife.97511 (PMC11856932; doi:10.7554/eLife.97511)
Supplement: Figure 7—figure supplement 1—source data 1. [file elife-97511-fig7-figsupp1-data1.zip › Figure 7–figure supplement 1—source data 1.pdf]

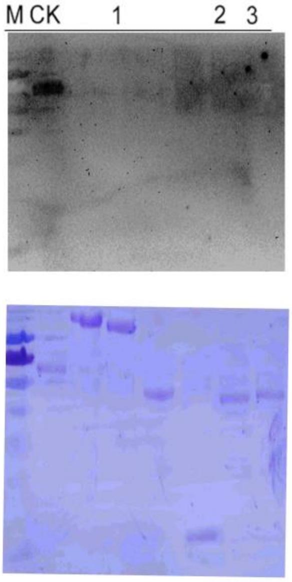

Figure 7–figure supplement 1—source data 1. Original files for western blot analysis displayed in Figure 7–figure supplement 1. Purified original recombinant proteins (without site-directed acetylation modification) without Kac modifications, validated by Western blot. 1: SUN, 2: ENO, 3 ArcA-2, CK: Kac-BSA, M: prestained protein marker. CK is Kac-BSA as a positive control. The lower section presents the PVDF membrane R350 staining for the loading amount control.
