# Supplementary material for: Aeromonas hydrophila CobQ is a new type of NAD+- and Zn2+-independent protein lysine deacetylase
Source: eLife. 2025 Feb 25;13:RP97511. doi: 10.7554/eLife.97511 (PMC11856932; doi:10.7554/eLife.97511)
Supplement: Figure 7—figure supplement 2—source data 1. [file elife-97511-fig7-figsupp2-data1.zip › Figure 7–figure supplement 2—source data 1.pdf]

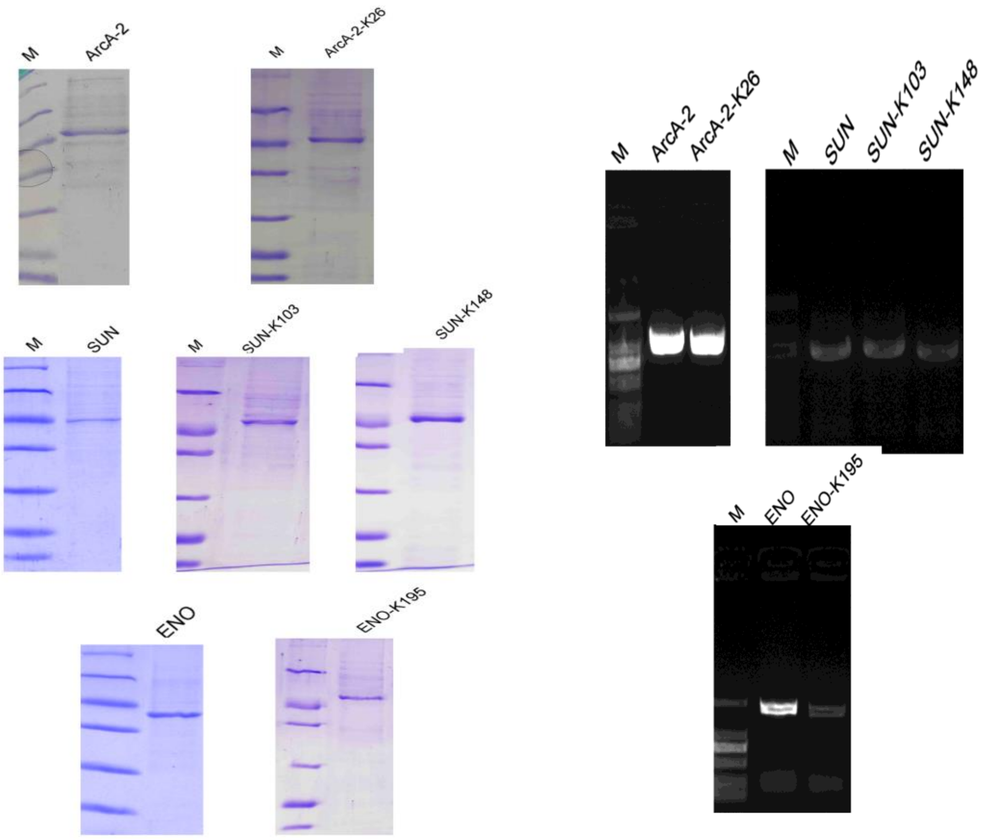

Figure 7–figure supplement 2—source data 1. Original files for PCR and SDS-PAGE analysis displayed in Figure 7–figure supplement 2. Characteristics of the overexpressed and purified recombinant target proteins. The PCR products (right) and SDS-PAGE results of the target recombinant or site-directed acetylated proteins. ArcA-2 and ArcA-2-K26; SUN, SUN-K103, and SUN-K148; ENO and ENO-K195.
