# Supplementary material for: Aeromonas hydrophila CobQ is a new type of NAD+- and Zn2+-independent protein lysine deacetylase
Source: eLife. 2025 Feb 25;13:RP97511. doi: 10.7554/eLife.97511 (PMC11856932; doi:10.7554/eLife.97511)
Supplement: Figure 8—source data 1. [file elife-97511-fig8-data1.zip › Figure 8—source data 1.pdf]

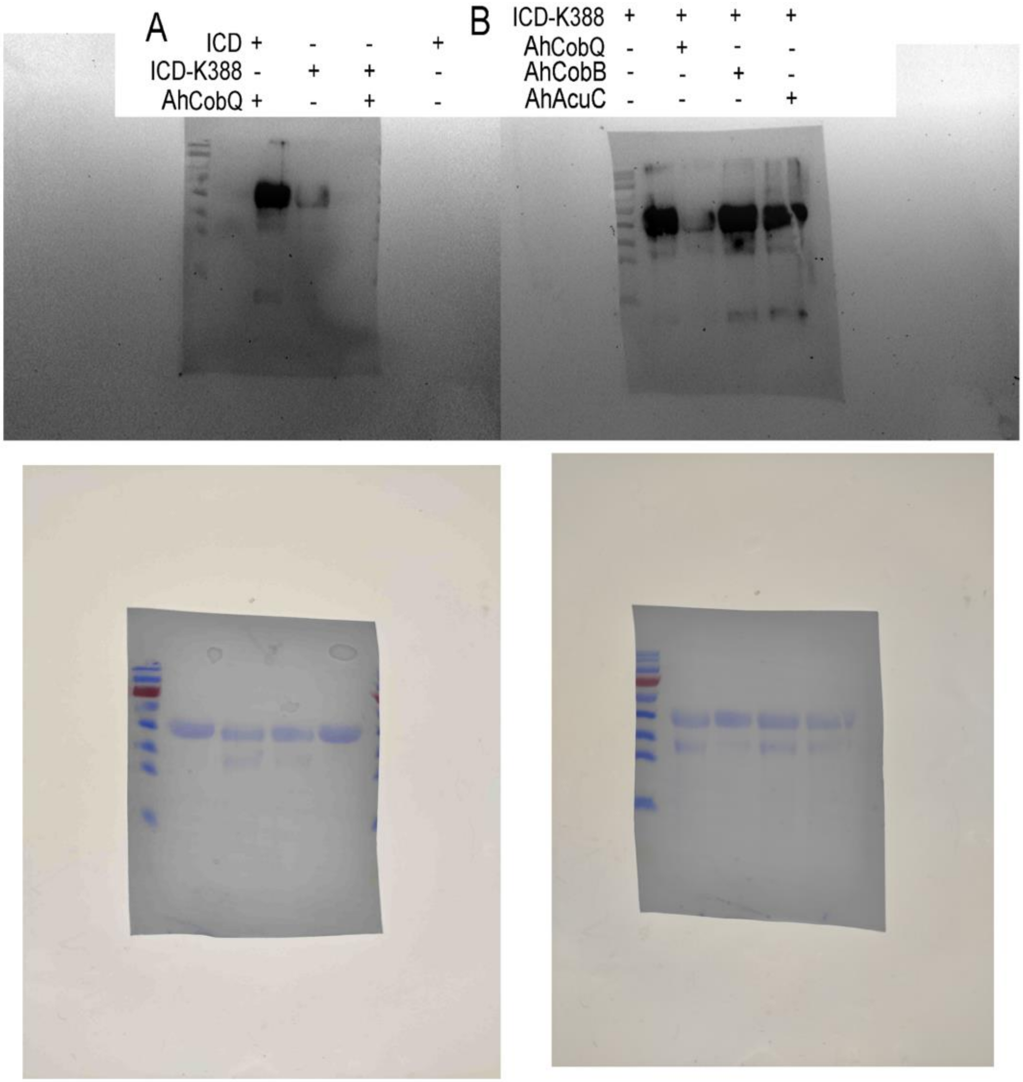

Figure 8—source data 1. Original files for western blot analysis displayed in Figure 8A, B. (A) western blot verified the deacetylation effect of AhCobQ on ICD and ICD-K388; (B) western blot verified the deacetylation effect of AhCobQ, AhCobB and AhAcuC on ICD-K388. The PVDF membrane R350 staining for the loading amount control was displayed under the WB results.
