# Supplementary material for: Aeromonas hydrophila CobQ is a new type of NAD+- and Zn2+-independent protein lysine deacetylase
Source: eLife. 2025 Feb 25;13:RP97511. doi: 10.7554/eLife.97511 (PMC11856932; doi:10.7554/eLife.97511)
Supplement: Supplementary file 1. — (a) Selected Kac peptide quantification among Kac-BSA and Kac-BSA incubated with CobQ and BSA without acetylation, by LC MS/MS. (b) Acetylation-modified upregulated proteins of AhCobQ-deleted strains and the identification of residue positions by LC MS/MS. (c) Chemicals and reagents used in this study. (d) Critical commercial assay kits used in this study. (e) Bacterial strains and plasmids used in this study. (f) Primer pairs used in this study. [file elife-97511-supp1.docx]

**Supplementary Tables**

**Supplementary file 1a. Selected Kac peptide quantification among Kac-BSA and Kac-BSA incubated with CobQ and BSA without acetylation, by LC MS/MS.**

**Supplementary file 1b. Acetylation-modified upregulated proteins of AhCobQ-deleted strains and the identification of residue positions by LC MS/MS.**

**Supplementary file 1c. Chemicals and reagents used in this study.**

**Supplementary file 1d. Critical commercial assay kits used in this study.**

**Supplementary file 1e. Bacterial strains and plasmids used in this study.**

**Supplementary file 1f. Primer pairs used in this study.**

**Table S1. Selected Kac peptide** **quantification of Kac-BSA, Kac-BSA incubated with CobQ, and BSA without acetylation by LC MS/MS**

| Kac peptide in BSA | Score | Intensity  Kac-BSA+CobQ | Intensity Kac-BSA | Intensity BSA |
| --- | --- | --- | --- | --- |
| LVNELTEFAK*TCVADESHAGCEK | 329.06 | 0 | 5.68E+07 | 0 |
| YNGVFQECCQAEDK*GACLLPK | 266.15 | 0 | 2.93E+08 | 0 |
| NYQEAK*DAFLGSFLYEYSR | 208.92 | 0 | 2.63E+08 | 0 |
| EYEATLEECCAK*DDPHACYSTVFDK | 194.62 | 0 | 1.92E+08 | 0 |
| LKPDPNTLCDEFK*ADEK | 182.52 | 0 | 2.52E+08 | 0 |
| ECCDK*PLLEK | 151.98 | 0 | 3.61E+08 | 0 |
| FWGK*YLYEIAR | 141.08 | 0 | 2.13E+08 | 0 |
| RHPYFYAPELLYYANK*YNGVFQECCQAEDK | 131.5 | 0 | 9.07E+07 | 0 |
| DAIPENLPPLTADFAEDK*DVCK | 127.75 | 0 | 3.62E+08 | 0 |
| ATEEQLK*TVMENFVAFVDK | 126.63 | 0 | 8.33E+08 | 0 |
| DVCK*NYQEAK | 124.82 | 0 | 3.38E+08 | 0 |
| K*FWGKYLYEIAR | 119.45 | 0 | 41218000 | 0 |
| AEFVEVTK*LVTDLTK | 101.53 | 0 | 1.28E+09 | 0 |
| CCAADDKEACFAVEGPK*LVVSTQTALA | 62.1 | 0 | 1.15E+08 | 0 |
| QEPERNECFLSHK*DDSPDLPK | 46.027 | 0 | 9.40E+07 | 0 |

Note: asterisk indicates Kac; best Maxquant Kac peptide score > 35 and localization probability > 0.75; BSA: bovine serum albumin.

**Table S2. Acetylation-modified upregulated proteins of AhCobQ-deleted strains and the identification of residue positions by LC MS/MS**

| Protein ID | Gene ID | Description | Positions (K) | Ratio (log2) | P-value |
| --- | --- | --- | --- | --- | --- |
| A0KGH3 | *eno* | Enolase | 195 | 11.04 | 0.004 |
| A0KIT5 | *glpT* | Glycerol-3-phosphate transporter | 152 | 6.62 | 0.001 |
| A0KEW8 | *sun* | Ribosomal RNA small subunit methyltransferase B | 148 | 5.85 | 0.016 |
| A0KJ32 | *iscS* | Cysteine desulfurase IscS | 93 | 5.41 | 0.010 |
| A0KMX2 | *tkt* | Transketolase | 302 | 5.26 | 0.022 |
| A0KPG3 | *recA* | Protein RecA | 279 | 5.18 | 0.022 |
| A0KG67 | *rpsF* | 30S ribosomal protein S6 | 104 | 5.17 | 0.017 |
| A0KF24 | *rplB* | 50S ribosomal protein L2 | 18 | 4.71 | 0.023 |
| A0KL52 | *adk* | Adenylate kinase | 48 | 4.56 | 0.003 |
| A0KMP9 | *rdgC* | Recombination-associated protein RdgC | 46 | 4.50 | 0.017 |
| A0KEW8 | *sun* | Ribosomal RNA small subunit methyltransferase B | 103 | 4.11 | 0.000 |
| A0KKE0 | *hrpA* | ATP-dependent helicase HrpA | 816 | 3.95 | 0.009 |
| A0KP36 | *cysD* | Sulfate adenylyltransferase subunit 2 | 115 | 3.60 | 0.024 |
| A0KHG1 | *glnD* | Bifunctional uridylyltransferase/uridylyl-removing enzyme | 461 | 3.51 | 0.008 |
| A0KHF8 | *ptsG* | PTS system, glucose-specific IIBC component | 380 | 3.41 | 0.006 |
| A0KKQ5 | *nrdA* | Ribonucleoside-diphosphate reductase | 718 | 3.30 | 0.034 |
| A0KKE0 | *hrpA* | ATP-dependent helicase HrpA | 1089 | 3.13 | 0.016 |
| A0KF44 | *rpsD* | 30S ribosomal protein S4 | 156 | 3.12 | 0.024 |
| A0KPV1 | *AHA_3863* | Pyruvate dehydrogenase E1 component | 507 | 3.06 | 0.018 |
| A0KNE8 | *glmM* | Phosphoglucosamine mutase | 34 | 2.98 | 0.041 |
| A0KEE9 | *gyrB* | DNA gyrase subunit B | 331 | 2.97 | 0.019 |
| A0KP36 | *cysD* | Sulfate adenylyltransferase subunit 2 | 82 | 2.96 | 0.045 |
| A0KLH8 | *AHA_2620* | CstA protein | 29 | 2.80 | 0.035 |
| A0KQH8 | *galK* | Galactokinase | 101 | 2.78 | 0.010 |
| A0KPW9 | *ftsA* | Cell division protein FtsA | 320 | 2.72 | 0.000 |
| A0KGI6 | *dnaG* | DNA primase | 241 | 2.70 | 0.029 |
| A0KQA4 | *rpoC* | DNA-directed RNA polymerase subunit beta | 74 | 2.57 | 0.041 |
| A0KJ75 | *AHA_1791* | RNA-binding protein | 81 | 2.52 | 0.021 |
| A0KKJ6 | *AHA_2274* | PhaF | 75 | 2.51 | 0.045 |
| A0KLU0 | *AHA_2733* | Phospho-2-dehydro-3-deoxyheptonate aldolase | 271 | 2.50 | 0.034 |
| A0KKH6 | *AHA_2253* | 3-Oxoacyl-[acyl-carrier-protein] synthase 2 | 329 | 2.49 | 0.025 |
| A0KM13 | *AHA_2809* | Putative transporter | 403 | 2.47 | 0.046 |
| A0KEJ9 | *glyS* | Glycine--tRNA ligase beta subunit | 112 | 2.47 | 0.029 |
| A0KK84 | *AHA_2161* | Aspartate/ornithine carbamoyltransferase, Asp/Orn binding domain family | 307 | 2.36 | 0.023 |
| A0KEL7 | *AHA_0149* | Acyl-CoA synthetase | 797 | 2.29 | 0.002 |
| A0KIV6 | *AHA_1671* | Polyphosphate kinase 2 | 172 | 2.27 | 0.034 |
| A0KJR9 | *guaB* | Inosine-5'-monophosphate dehydrogenase | 110 | 2.15 | 0.050 |
| A0KI76 | *dsdA* | Probable D-serine dehydratase | 118 | 2.13 | 0.044 |
| A0KN87 | *leuS* | Leucine--tRNA ligase | 561 | 2.12 | 0.010 |
| A0KI74 | *icd* | Isocitrate dehydrogenase [NADP] | 388 | 2.07 | 0.039 |
| A0KND9 | *pnp* | Polyribonucleotide nucleotidyltransferase | 523 | 2.03 | 0.003 |
| A0KHG1 | *glnD* | Bifunctional uridylyltransferase/uridylyl-removing enzyme | 205 | 1.92 | 0.001 |
| A0KN82 | *AHA_3242* | PhoH family protein | 219 | 1.83 | 0.025 |
| A0KN37 | *AHA_3197* | Methyl-accepting chemotaxis protein | 262 | 1.81 | 0.029 |
| A0KGR9 | *mutL* | DNA mismatch repair protein MutL | 200 | 1.73 | 0.000 |
| A0KNL1 | *cysH* | Phosphoadenosine phosphosulfate reductase | 193 | 1.60 | 0.026 |
| A0KHG1 | *glnD* | Bifunctional uridylyltransferase/uridylyl-removing enzyme | 686 | 1.54 | 0.049 |
| A0KQG4 | *argF* | Ornithine carbamoyltransferase | 293 | 1.39 | 0.005 |
| A0KJB6 | *AHA_1833* | Uncharacterized protein | 401 | 1.33 | 0.021 |
| A0KQG6 | *arcA-2* | Arginine deiminase | 26 | 1.02 | 0.047 |
| A0KES5 | *AHA_0213* | S-formylglutathione hydrolase | 17 | 0.76 | 0.042 |
| A0KQS4 | *ilvD* | Dihydroxy-acid dehydratase | 320 | 0.73 | 0.009 |

**Table S3. Chemicals and reagents used in this study.**

| Reagent | Company | Lot number |
| --- | --- | --- |
| Yeast extract | Oxoid | Cat# 4304391-02 |
| Tryptone | Oxoid | Cat# 3153896 |
| Sodium chloride | Sinophem | Cat# 10019318 |
| Agar powder | Solarbio | Cat# A8190 |
| Ligase | Vazyme | Cat# C113 |
| 2 x Rapid Taq Master Mix | Vazyme | Cat# P222 |
| 2 x Phanta Max Master Mix | Vazyme | Cat# P515-03 |
| Ampicillin | Aladdin | Cat# 69-52-3 |
| Chloramphenicol | Solarbio | Cat# A8193 |
| Nicotinamide | Sigma Aldrich |  |
| BSA | Sigma Aldrich |  |
| IPTG | Solarbio |  |
| N-acetyllysine | Sigma Aldrich |  |
| NAD^+^ | Sigma Aldrich |  |
| Isocitric acid | Sigma Aldrich |  |
| NADPH | Sigma Aldrich |  |
| NADP^+^ | Sigma Aldrich |  |
| PEP/2-PGE | Sigma Aldrich |  |
| Acetylated modified antibody | Jingjie PTM | PTM-101 |
| HRP Goat-mouse IgG | CWBIO | HA1006 |
| ATP | Sigma Aldrich |  |
| Ni-NTA agarose beads | Yeasen |  |
| C18 ZipTips | Millipore |  |
| Acetonitrile (ACN) | Sigma Aldrich |  |
| Formic acid (FA) | Sigma Aldrich |  |
| Sheep blood | Solarbio |  |
| Skim milk | Wako |  |
| Crystal Violet | Sigma Aldrich |  |
| Tris (pH 8.0) | Sangon |  |
| Dithiothreitol (DTT) | Sigma Aldrich |  |
| Iodoacetamide (IAA) | Sigma Aldrich |  |
| Trypsin | Promega |  |
| PVDF | Bio-RAD |  |
| hPLG | Hepeng |  |

**Table S4. Critical commercial assays kits used in this study.**

| Kits | Company | Lot number |
| --- | --- | --- |
| Plasmid extraction kit | Magen | Cat# P1001-03C |
| Gel recovery kit | Magen | Cat# D2111-03 |
| DNA extraction kit | Magen | Cat# D3146-02 |
| Mut Express II Fast Mutagenesis kit | [TransGen Biotech](https://www.so.com/link?m=bsNdbBdOFsB2/NKScvb0A/QanmX3LKNtx0r2RmBqaUeQg/o4ldsJyT0pBfdlVO6pVusGZcRCtrcMsTqnF1As/QyhwXJN51f+gVZL2Vxc8ZXUd8cjtG9lCe+eKS274lT+kqIUfSI5lVX4=) | Cat# FM111-01 |
| ClonExpress MultiS One Step Cloning kit | Vazyme | C113 |
| ClonExpress II One Step Cloning kit | Vazyme | C112 |

**Table S5. Bacterial strains and plasmids used in this study**

| Strain name | Strain type | | Other information |
| --- | --- | --- | --- |
| A. *hydrophila* ATCC 7966 | Wild type strain (WT) |  | |
| *A. hydrophila* LP-2 | Virulent strain |  | |
| *E.coli* BL21 (DE3) | Transformed host (Overexpression) |  | |
| *E.coli* DH5α | Transformed host (Site-directed mutagenesis) |  | |
| *E.coli* MC1061 | Transformed host (Knockout) |  | |
| *E.coli* S17-1λpir | Transformed host (Knockout) |  | |
| *ΔahcobQ* | Knockout strain |  | |
| *ΔahcobB* | Knockout strain |  | |
| *ΔahacuC* | Knockout strain |  | |
| AhCobQ-6His tag | Overexpression strain | pET-32a | |
| AhCobQ-GST tag | Overexpression strain | pGEX-KG | |
| AhCobB-32a | Overexpression strain | pET-32a | |
| AhAcuC-32a | Overexpression strain | pET-32a | |
| AhCobQ_1–179_ | Overexpression strain | pET-32a | |
| AhCobQ_179–265_ | Overexpression strain | pET-32a | |
| AhCobQ_189–265_ | Overexpression strain | pET-32a | |
| AhCobQ_189–255_ | Overexpression strain | pET-32a | |
| AhCobQ_189–250_ | Overexpression strain | pET-32a | |
| AhCobQ_189–245_ | Overexpression strain | pET-32a | |
| AhCobQ_189–240_ | Overexpression strain | pET-32a | |
| AhCobQ_195–255_ | Overexpression strain | pET-32a | |
| AhCobQ_200–255_ | Overexpression strain | pET-32a | |
| AhCobQ_179–255_ | Overexpression strain | pET-32a | |
| AhCobQ_179–245_ | Overexpression strain | pET-32a | |
| AhCobQ_179–235_ | Overexpression strain | pET-32a | |
| AhCobQ_179–225_ | Overexpression strain | pET-32a | |
| AhCobQ_199–265_ | Overexpression strain | pET-32a | |
| AhCobQ_209–265_ | Overexpression strain | pET-32a | |
| AhCobQ_219–265_ | Overexpression strain | pET-32a | |
| SUN-K103 | Site-directed acetylation strain | pET-21b | |
| SUN-K148 | Site-directed acetylation strain | pET-21b | |
| ENO-K195 | Site-directed acetylation strain | pET-21b | |
| ArcA-2-K26 | Site-directed acetylation strain | pET-21b | |
| ICD-K388 | Site-directed acetylation strain | pET-21b | |
| pET-32a | Expression vector, HIS-tag, Amp^R^ |  | |
| pET-21b | Expression vector, HIS-tag, Amp^R^ |  | |
| pGEX-KG | Expression vector, GST-tag, Amp^R^ |  | |
| pRE112 | Suicide vector, oriVR6Kγ, Cm^R^, sacB |  | |
| pTECH-MbAcK3RS (IPYE) | Site-directed acetylation co-transformation vector |  | |

**Table S6. Primer pairs used in this study**

| Primer name | Oligonucleotide sequence (5′ → 3′) |
| --- | --- |
| Δ*ahcobB*-P1 | cgatcccaagcttcttctagaCTTGGGATAGGTGGTGAACGG |
| Δ*ahcobB*-P2 | ggaatcagtcGCTATTGTGCGACTGGACAGTTG |
| Δ*ahcobB*-P3 | gcacaatagcGACTGATTCCCTCTGGCTACTTCT |
| Δ*ahcobB*-P4 | catgaattcccgggagagctcGCGAGCAGAGCGTCTACCTG |
| Δ*ahcobB*-P5 | TCAGGGACCGCGCATCTCCATCCAG |
| Δ*ahcobB*-P6 | ATGGTGCAGTCAGCGAAACACATCG |
| Δ*ahcobB*-P7 | GCAACCAACAGCAGTTTGT |
| Δ*ahcobB*-P8 | GCCCTGCCGCTGA |
| Δ*ahcobQ*-P1 | catgaattcccgggagagctcCGAGCTGAGCTCAGGTCTGTTG |
| Δ*ahcobQ*-P2 | ctcTGTATCAATCCTGCCTCGTTGTT |
| Δ*ahcobQ*-P3 | gaggcaggattgatacaGAGATACCACCCAAGTTCGGG |
| Δ*ahcobQ*-P4 | cgatcccaagcttcttctagaCTCGGTCAACGGCACCGC |
| Δ*ahcobQ*-P5 | GTGATTGTTTGGACGGTTGCC |
| Δ*ahcobQ*-P6 | GATGTTACCTCATGAGCACGCTG |
| Δ*ahcobQ*-P7 | TGCAGCCACGCAGCGACGGTTTGCT |
| Δ*ahcobQ*-P8 | GGCGCCATTTGACCTGGTCACGATG |
| Δ*ahacuC*-P1 | catgaattcccgggagagctcTGGTTGTCGCCTTTGAAGGC |
| Δ*ahacuC*-P2 | cgataaacggGAAACGAGTGCTCGCGCG |
| Δ*ahacuC*-P3 | cactcgtttcCCGTTTATCGCTTCGTCAATC |
| Δ*ahacuC*-P4 | cgatcccaagcttcttctagaCTGTTTGTGCTGGGCGCC |
| Δ*ahacuC*-P5 | GTGCGACAAGGGGAGCCTG |
| Δ*ahacuC*-P6 | CTAGCCGTAGCGCTTTTTCGCC |
| Δ*ahacuC*-P7 | ACCAACCCCTACGTCGTACAGC |
| Δ*ahacuC*-P8 | GTTCGGCCGCTTACCGGTG |
| AhCobQ-32a-F | gctgatatcggatccgaattcGTGATTGTTTGGACGGTTGCC |
| AhCobQ-32a-R | ctcgagtgcggccgcaagcttTCATGATGTTACCTCATGAGCACG |
| AhCobB-32a-F | gctgatatcggatccgaattcATGGTGCAGTCAGCGAAACAC |
| AhCobB-32a-R | ctcgagtgcggccgcaagcttTCAGGGACCGCGCATCTC |
| AhAcuC-32a-F | gctgatatcggatccgaattcGTGCGACAAGGGGAGCCT |
| AhAcuC-32a-R | ctcgagtgcggccgcaagcttCTAGCCGTAGCGCTTTTTCG |
| AhCobQ_1–179_-F | gctgatatcggatccgaattcGTGATTGTTTGGACGGTTGCC |
| AhCobQ_1–179_-R | ctcgagtgcggccgcaagcttTCAGAACTTCTCCCGCTTGG |
| AhCobQ_179–265_-F | gctgatatcggatccgaattcATGCGTTATACCGTCATTCCCA |
| AhCobQ_179–265_-R | ctcgagtgcggccgcaagcttTCATGATGTTACCTCATGAGCACG |
| AhCobQ_189–265_-F | gctgatatcggatccgaattcATGGACAAGCGAACCCGTGCC |
| AhCobQ_189–265_-R | ctcgagtgcggccgcaagcttTCATGATGTTACCTCATGAGCACG |
| AhCobQ_189–255_-F | gctgatatcggatccgaattcATGGACAAGCGAACCCGTGCC |
| AhCobQ_189–255_-R | ctcgagtgcggccgcaagcttTCACTCTTGCGCATCAAGATAGTTGA |
| AhCobQ_189–250_-F | gctgatatcggatccgaattcATGGACAAGCGAACCCGTGCC |
| AhCobQ_189–250_-R | ctcgagtgcggccgcaagcttTCAATAGTTGAGCAGGGTCTCATAGGC |
| AhCobQ_189–245_-F | gctgatatcggatccgaattcATGGACAAGCGAACCCGTGCC |
| AhCobQ_189–245_-R | ctcgagtgcggccgcaagcttTCACTCATAGGCATAGGTGCCACG |
| AhCobQ_189–240_-F | gctgatatcggatccgaattcATGGACAAGCGAACCCGTGCC |
| AhCobQ_189–240_-R | ctcgagtgcggccgcaagcttTCAGCCACGGCTGCTCGGCGA |
| AhCobQ_195–255_-F | gctgatatcggatccgaattcATGTCGCTGATGACTCTGCAGTCC |
| AhCobQ_195–255_-R | ctcgagtgcggccgcaagcttTCACTCTTGCGCATCAAGATAGTTGA |
| AhCobQ_200–255_-F | gctgatatcggatccgaattcATGCAGTCCATCAAGGAGCAGCAC |
| AhCobQ_200–255_-R | ctcgagtgcggccgcaagcttTCACTCTTGCGCATCAAGATAGTTGA |
| SUN-K103-F | gagaccgtcaacgccgtcTAGctgctcaagggcacctccttgcgc |
| SUN-K103-R | CTAgacggcgttgacggtctcggccaccgcggcgtgagccggaat |
| SUN-K148-F | ccggagtggctcaccTAGcggctgcgccaggcctatccggatgag |
| SUN-K148-R | CTAggtgagccactccgggtggccgagacgaatgctggggacacggt |
| ENO-K195-F | gtgttccacaacctggccTAGgtgctgaagtccaagggctacaac |
| ENO-K195-R | CTAggccaggttgtggaacacttcagcgcccatgcggacagcttctttc |
| ArcA-2-K26-F | cccaacctcagtctgTAGcgtctgactccttccaactgccaggat |
| ArcA-2-K26-R | CTAcagactgaggttggggcggtgcaacatgacacggcgcaatt |
| ICD-K388-F | gcggcgatccgcaacTAGaccgtcacttatgacttcgagcgtctg |
| ICD-K388-R | CTAgttgcggatcgccgcttccatccccttgatgatgagatcggc |
| SUN-F | gctgatatcggatccgaattcATGAAAACACGCGCACAGG |
| SUN-R | ctcgagtgcggccgcaagcttTTACCGCTTGATCAGCTTGGC |
| ENO-F | gctgatatcggatccgaattcATGTCCAAGATCGTTAAAGTGATCG |
| ENO-R | ctcgagtgcggccgcaagcttTTAAGCCTGGTTCTTCACTTCTTTC |
| ArcA-2-F | aatgggtcgggatccgaattcATGAGCAAATTTTATGTAGGTTCTGAA |
| ArcA-2-R | ctcgagtgcggccgcaagcttTTAGATGCCGTCGCGTTCC |
| ICD-F | gctgatatcggatccgaattcATGGAAAGCAAAGTAGTTATCCCG |
| ICD-R | ctcgagtgcggccgcaagcttTTACATCTGGTCGACCATGTCCT |
